# The use of digital outcome measures in clinical trials in rare neurological diseases: a systematic literature review

**DOI:** 10.1186/s13023-023-02813-3

**Published:** 2023-08-02

**Authors:** Margaux Poleur, Theodora Markati, Laurent Servais

**Affiliations:** 1 Department of Neurology, Liege University Hospital Center, Liège, Belgium; 2 MDUK Oxford Neuromuscular Centre and NIHR Oxford Biomedical Research Centre, University of Oxford, Oxford, UK; 3 Neuromuscular Reference Center, Division of Paediatrics University, Hospital University of Liège, Liège, Belgium; 4 Centre de Référence des Maladies Neuromusculaires, Centre Hospitalier Régional de la Citadelle, Boulevard du 12eme de Ligne 1, 4000 Liège, Belgium

## Abstract

Developing drugs for rare diseases is challenging, and the precision and objectivity of outcome measures is critical to this process. In recent years, a number of technologies have increasingly been used for remote monitoring of patient health. We report a systematic literature review that aims to summarize the current state of progress with regard to the use of digital outcome measures for real-life motor function assessment of patients with rare neurological diseases. Our search of published literature identified 3826 records, of which 139 were included across 27 different diseases. This review shows that use of digital outcome measures for motor function outside a clinical setting is feasible and employed in a broad range of diseases, although we found few outcome measures that have been robustly validated and adopted as endpoints in clinical trials. Future research should focus on validation of devices, variables, and algorithms to allow for regulatory qualification and widespread adoption.

**Supplementary Information:**

The online version contains supplementary material available at 10.1186/s13023-023-02813-3.

## Introduction

In the European Union (EU), a disease is defined as rare if it affects fewer than five in 10,000 people across the EU [1]. In the United States (US), the Orphan Drug Act defines a rare disease as a disease or condition that affects less than 200,000 people in the US [2]. Around 10,000 rare diseases have been identified according to these different definitions and about 5% of the European population is thought to be affected by one of them, many of which have neurological manifestations. These diseases are often associated with high mortality and disability, which result in large societal costs [3]. Drug development for rare diseases is especially challenging due to the small number of subjects eligible for inclusion in clinical trials. Trials in this field tend to be small, single-arm, non-randomized, and open label [4]. As define by the US Food and Drug Administration [5], a clinical outcome assessment is a measure that describes or reflects how a patient feels, functions, or survives. In clinical trials for rare diseases, outcome measures are often subjective, depending on both patient motivation and rater [6]. Moreover, test results only reflect the patient’s condition at a specific timepoint, and severity of symptoms often varies with time.

Nevertheless, the precision and the objectivity of the outcome measures are critical [7]. Digital biomarker could to a certain extend fill this unmet need. Digital biomarkers have been defined by the European Medicines Agency (EMA) as an objective, quantifiable measure of physiology or behavior used as an indicator of biological, pathological process or response to an exposure or an intervention that is derived from a digital measure [8] (e.g., step length). Outcomes are defined as measures chosen to assess the impact of an intervention [9]. For most rare diseases, clinical outcomes have not been qualified or validated to the same rigor, or methodological approach and in large cohorts as they have in more common diseases. We define an outcome as validated or partially validated if it has been studied in adequate and well-controlled studies with full characterization of its psychometric properties after original discovery studies. Outcome qualification is a longer and codified process to gather regulatory authorities qualification of this outcome within a well-defined context of use [5]. Thus, in clinical trials for rare diseases, outcome measures are often subjective, depending on both patient motivation and rater [6]. Moreover, test results only reflect the patient’s condition at a specific timepoint, and severity of symptoms often varies with time.

In recent years, an increasing number of technologies have been used for remote monitoring of health [10]. Remote monitoring provides the opportunity to reduce cohort sizes and time-to-endpoint for clinical trials [11] while providing a more accurate representation of the patient’s condition than evaluations conducted in clinic facilities at a few time points.

Efforts to develop digital outcome measures have focused mostly on neurological disorders that are not classified as rare [12, 13], but these measures are increasingly used to evaluate patients who have rare diseases. The use of digital outcome measures is expected to increase with the development of innovative technologies and artificial intelligence [14].

In neuromuscular disorders, a recent literature review highlighted the increasing use of sensors to assess motor activity in the real-life and the ability of these sensors to overcome many issues of traditional evaluations [15]. Nevertheless, only one digital outcome measure, the 95th centile of stride velocity, is currently qualified by a regulatory agency for the use in Duchenne muscular dystrophy [16].

The purpose of this systematic review is to summarize the current state of progress with regard to the use of digital outcome measures for real-life motor function assessment of patients affected by rare neurological diseases. We also summarize the psychometric properties that have been assessed for each outcome providing a snapshot of where we stand currently in the process of developing and qualifying digital outcomes in rare diseases.

## Methods

Methods for identification of relevant clinical studies are described in Additional file 1. Data extracted from individual studies is presented in Additional file 2.

## Results

### Selection of clinical studies

**Fig. 1:**
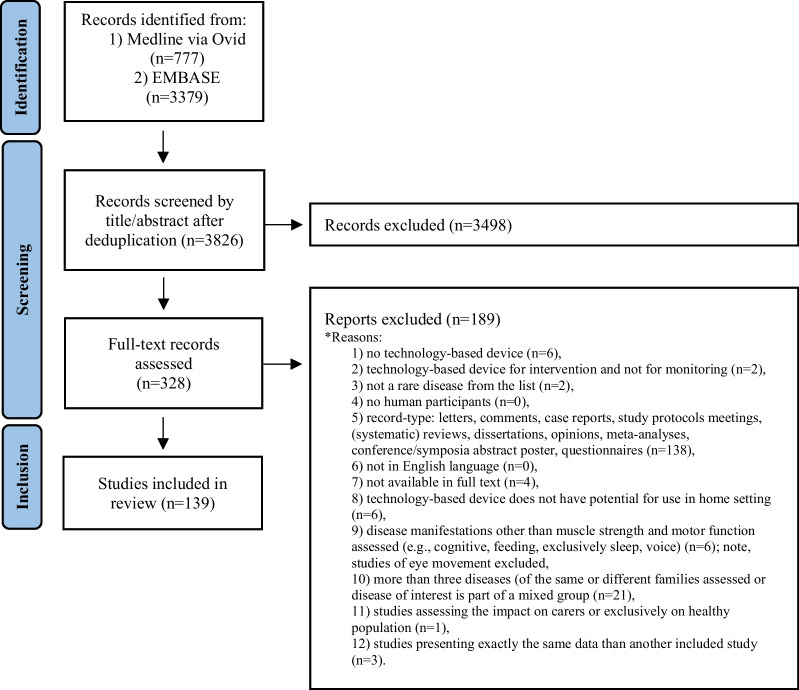
Flow-chart of selection process

This review provides a summary of the use of digital outcome measures in clinical trials listed in Medline and Embase. Figure 1 is a flow-chart of the process used for inclusion of clinical studies in our analysis. Our search of published literature identified 3826 records, of which 139 were included. Amongst these studies, 51 focused on neuromuscular disorders, 42 on movement disorders, 16 on genetic ataxias, nine on multisystemic rheumatological diseases, and 21 miscellaneous neurodevelopmental disorders. Information on study designs and collected data are summarized in Table 1.

**Table 1:** Summary by disease of number of studies and their design, population and main findings

Disease	Population		Study design			Type of technology				Setting		Parameters assessed				
	Number of studies	Number of patients	Interventional	With controls	Longitudinal	Accelerometer/gyroscopy	EMG	App/software	Other	Home	Controlled environment	Physical activity	Gait analysis	Upper limb	Tremor	Other
DMD	18	550	2	7	6	17		2		13	5	10	2	5		5
ALS	15	2323	2	4	8	10	2	4	1	8	8	5	1	3	1	7
CMT	6	392		2	1	6				4	2	4	1		1	
DM	4	142		3		3			1	2	2	1	3	1		2
FSHD	4	70	1	1	2	4		1		2	2		3	3		
MG	2	60		1		2				2		2				
SMA	1	81			1	1				1				1		
SBMA	1	54	1		1	1				1		1				
HD	26	738 (+ 98 premani-fest)	3	23	6	23		3		10	19	8	10	4		14
PSP	7	112	1	5	1	7					7		4			3
FD	9	414	1	3		7	2		4		9			1	5	3
SCA	9	322 (+ 80 premani-fest)		8	1	9				2	8	1	6	1		4
HSP	3	143		1	1	3					3		3			
FXS	2	38 (+ 15 premani-fest)		2		2					2		2			
FRDA	2	43		2	1	2		1	1	1	2		1	2		1
Sarcoido-sis	5	793		1		5				5		5				
Dermato- myositis	3	79		2		3				3		3				
Scleroder-ma	1	27		1		1				1		1				
PWS	8	143	2	5	2	8				5	3	5	3			2
Pompe disease	2	54			1	1	1		1	2	1	1				1
MPS	1	8		1		1				1		1				
Fabry	1	17		1		1				1		1				
GM2	1	8			1			1	1	1		1				
NP-C	2	22				2				2		1				1
Rett syndrome	5	150	1		1	5				4	2	5				
TSC	1	30				1					1	1				1
Narcolepsy	1	56				1				1		1				
Number of studies	139	6590	14	73	34	126	5	12	7	72	76	58	39	20	7	43

*DMD* Duchenne muscular dystrophy, *ALS* amyotrophic lateral sclerosis, *CMT* Charcot-Marie-Tooth, *DM* myotonic dystrophic, *FSHD* facioscapulohumeral dystrophy, *MG* myasthenia gravis, *SMA* spinal muscular atrophy, *SBMA* spinal and bulbar muscular atrophy, *HD* huntington disease, *PSP* progressive supranuclear palsy, *FD* focal dystonia, *SCA* spinocerebellar ataxias, *HSP* hereditary spastic paraplegia, *FXS* Fragile X Syndrome, *FRDA* Friedreich’s ataxia, *PWS* Prader–Willi syndrome, *MPS* mucopolysaccharidosis, *GM2* GM2 gangliosidosis, *NP-C* Niemann–Pick type C, *TSC* tuberous sclerosis complex

### Types of outcomes

In the studies evaluated, very few outcomes were precisely defined and even fewer were completely validated and ready for qualification. Event detection or frequency directly derived from sequential detection of an event was often measured. Physical activity was measured in various ways including number of steps and time spent in different levels of physical activity. Outcomes related to gait ranged from time-distance variables (i.e., stride length, velocity) to more elaborated variables resulting from machine-learning approaches. Methods for upper limb assessments were also heterogeneous. Other functions monitored included balance and body sway.

### Choice and position of the sensors

**Fig. 2:**
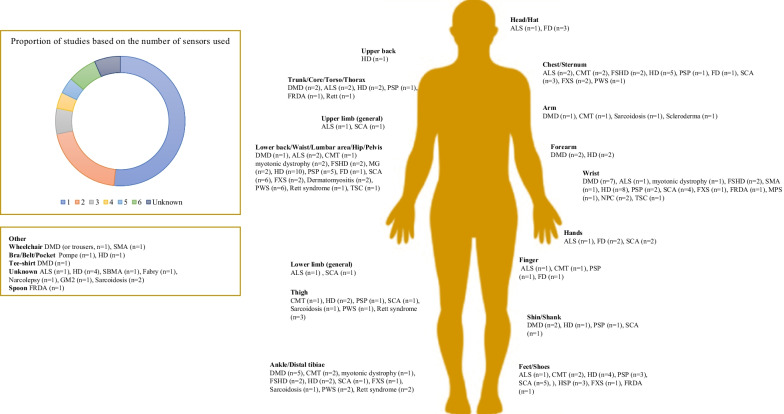
Numbers and placements of wearable and portable inertial sensors. Abbreviation: Duchenne Muscular Dystrophy (DMD), Amyotrophic Lateral Sclerosis (ALS), Charcot-Marie-Tooth (CMT), Myotonic Dystrophic (DM), Facioscapulohumeral dystrophy (FSHD), Myasthenia Gravis (MG), Spinal Muscular Atrophy (SMA), Spinal and bulbar muscular atrophy (SBMA), Huntington Disease (HD), Progressive supranuclear palsy (PSP), focal dystonia (FD), Spinocerebellar ataxias (SCA), Hereditary spastic paraplegia (HSP), Fragile X Syndrome (FXS), Friedreich’s ataxia (FRDA), Prader–Willi syndrome (PWS), mucopolysaccharidosis (MPS), GM2 gangliosidosis (GM2), Niemann–Pick type C (NP-C)**,** Tuberous sclerosis complex (TSC)

Wearable or portable devices based on inertial technology were by far the most frequently used sensors, although pressure or surface electromyographic sensors were also employed. Technologies included applications and software. Critical to effective motor assessment is the number and position of the sensors as sensors only assess the movement of the segment where they are placed, and sensor number and position may influence patient compliance. There was large variability in the numbers and positions of sensors in the same patient populations. The locations of the sensors and the numbers of relevant studies are presented in Fig. 2.

### Psychometric properties

Psychometric properties that were evaluated in this review are summarized in Table 2. We found 92 studies that described use of psychometric evaluation. Most focus on validity assessment through correlation with standard outcomes and distinction between normal and pathological while reliability and sensitivity to change were assessed in only 16 and 21 studies, respectively.

**Table 2:** Psychometric properties

Disease	Validity	Reliability	Sensitivity to negative change	Sensitivity to positive change
*Actigraph, Motionlogger Watch; Ambulatory Monitoring, Ardsley, NY, USA*				
DMD	PIM score correlated with six min walk distance and knee extension strength [138]			
*Actimyo, Sysnav, Vernon*				
DMD	For the MoviPlate and BBT, all variables correlated with the functional scores. Norm of the angular velocity, power, elevation rat correlated with the Minnesota scores and the writing task. The mean of the rotation rate and mean of the elevation rate had the best correlations with task scores [43]	All ActiMyo variables showed high to very high reliability as assessed using ICC values. The mean of the rotation rate and mean of the elevation rate had the best reliability scores [43]		
SMA	MFM 32 and grip strength were found to have high correlation with wrist acceleration as measure by ActiMyo [42]		Wrist angular velocity, the wrist acceleration, the wrist vertical acceleration and power decreased significantly over six and 12 months as measures in patients with Type 2 SMA and non-ambulant with Type 3 [42]	
FSHD	Strong correlations between representative (stride lenght and velocity) gait variables and MMT variables [44]	All the measurements showed high reliability according to ICC values (all > 0.9). Representative variables showed lower SEMs (all < 0.03) compared to cumulative variables (40.2 and 28.5) [44]	Significant decline for median speed, SV95C and SL95C at 3 months; The SRM of median speed, SV95C and 95th centile length (was significantly elevated at 3 months [44]	
*ActivPal, PAL Technologies Ltd, Glasgow, UK*				
Sarcoidosis	Patients with sarcoidosis had s lower daily step counts than controls and a trend towards fewer sit-to-stand transitions each day. Correlation between 6MWD and the daily step count [102]			
Dermatomyositis	Patients with inactive disease had lower physical activity levels compared with controls [139]			
Rett syndrome				The sedentary time was decreased after intervention. Positive effects with small to medium effect sizes were seen in sedentary time [122]
*Actiwatch 2, AW2; Philips Respironics, Bend, Oregon*				
DMD	Habitual daytime activity level was associated with 6MWT performance [140]			
*AD_BRC sensor*				
HD	Sensor derived velocity was significantly higher in healthy controls and premanifest HD when compared to HD. Step and stride length was significantly longer in controls and premanifest HD when compared to HD. Significant diffences between subject groups across all four balance tasks [58]			
*AIM- system*				
FRDA	Smoothness, trajectory length, duration, and range of motion were the most effective to distinguish individuals with FRDA from controls. Strong correlation between the AIM-S score and the mFARS score and the NeuroUL score [130]	The AIM-S score showed good to excellent test re-test reliability [130]	The sensitivity of the AIM-S to detect deterioration in upper limb function was greater than other measures [130]	The AIM-S score showed minimal variability [130]
*ASUR-Autonomous Sensing Unit Recorder + Physilog*				
DMD	Except cadence, all gait parameters showed significant differences between patients and controls. All gait parameters were more affected in the moderate group compared to the mild group. Moderate correlations between the MFM and: stride velocity, cadence and spectral entropy [21]			
				Duration of the walking episodes or the succession of two or three walking episodes lasting more than 30 s were the most improved after prednisolone treatment [24]
*Biopack*				
FD	The incidence of tremor was significantly higher in dystonic patients as compared to controls [80]			
*BioStampRC® wearable sensors developed by MC10 Inc*				
HD	The average truncal Chorea Index was higher in individuals with HD than in controls. Individuals with HD walked less and took longer duration steps than the other groups. Correlation between the UHDRS maximal truncal chorea score and the average truncal Chorea Index [49]		Walk speed of individuals with HD showed a significant decrease over 12 months but not time spent lying, sitting, standing, and walking, truncal Chorea Index, step count and duration.[49]	
	Individuals with HD spent over 50% of the total time lying down, more than individuals with prodromal HD, PD, and controls [47]			
*Cambridge Neurotechnology AW4 or Respironics Actiwatch 2 actigraph*				
MPS	Children with MPS III had significantly higher activity levels during the early morning hours compared to controls [117]			
*CHDR Monitoring Remotely (CHDR MORE) platform*				
FSHD	The classification between patients with FSHD and controls with 93% accuracy, 100% sensitivity, and 80% specificity. Features relating to smartphone acceleration, app use, location, physical activity, sleep, and call behavior were the most salient features for the classification [39]			
*Computational Motor Objective Rater (CMOR)*				
FD	Head posture severity correlated with severity ratings from movement disorders neurologists using both the TWSTRS-2 and an adapted version of the Global Dystonia Rating Scale [85]			
*DynaPort Move Monitor, McRoberts, The Hague, The Netherlands*				
MG	Patients perform less vigorous PA, spend more time sedentary and engage in less and shorter durations of MVPA than controls. Habitual PA correlated positively with 6 min walking distance [41]			
	PAL was lower in patients than in controls. No correlation between disease severity and number of steps/day nor between disease severity and PAL [40]			
*EBIMU 9DOF*				
FD	All parameters were validated by comparing with the TSWTRS-total score and the TWSTRS-severity score. The MAV parameters showed a higher correlation with clinical severity than RA parameters [84]	The ICCs were all more than 0.9 for the four parameters, which showed good agreement for all parameters [84]		
*eMotion Faros 180*				
ALS	Average daytime active; percentage of daytime active; total daytime activity score; total 24-h activity score showed correlations with ALSFRS-R total and gross motor domain scores [141]		All the activity endpoints investigated changed from baseline over the course of the study (48 weeks) to indicate a decline in physical activity over time [141]	
*Fitbit One TM*				
Pompe disease	Mean step count differed by age (*p* < 0.01), diagnostic delay (*p* < 0.05), disease duration (*p* < 0.05), and ambulatory status (*p* < 0.05) Patient-reported “fatigue and pain” score was inversely correlated with step count (*p* < 0.05) and peak 1-min activity (*p* < 0.01) [115]			
*GaitUp Physilog 5, Lausanne, Switzerland*				
FRDA	Controls were significantly more active than the FRDA group. Peak swing and stance period were the most discriminatory parameters. Correlation between the 25-foot walk test and the cadence. Digitally derived stride width strongly reflected the risk of falling. Mean stride width from the real-world gait analysis dropped with GAA repeat length of the short allele [99]			
*GENEActiv, Kimbolton, Cambs, United Kingdom*				
HD	The developed system achieved 98.78% accuracy in discriminating between healthy and HD participants. Correlation between MIS and mULMS [67]			
	HD participants had a greater percentage of walking bouts with irregular movements, lower walking consistency and higher across-bout variability compared to controls. Negative correlation between UHDRS-TMS scores and walking time and steps per day. Moderate correlation within-bout and across-bout gait consistency and the clinical measures of upper extremity chorea and total chorea [56]			
DM1	A significant difference between the myotonic dystrophy group and the controls was detectable at each test. Stronger correlation values between the 6MWT distance and the ankle-worn accelerometry units. No correlation identified in the DM1 group for the wrist-worn devices [142]	High intra-accelerometer reliability (p < 0.001). There was no inter-accelerometer reliability between wrist-worn devices and ankle-worn [142]		
*G-SensorVR, BTS Bioengineering, Italy*				
FD	All the spatio-temporal parameters of the sub-phases of the Timed up and go test (turning, standing-up and sitting-down from a chair) had a significantly higher duration in cervical dystonia patients compared to the controls [83]			
PWS	PWS exhibited significantly reduced values of HR in the antero-posterior and vertical directions comparing to controls [109]			
*GT3X Actigraph, Manufacturing Technology, Inc./GT9X*				
HD	PA was lower in patients compared to controls [48]			
Dermatomyositis	Patients with inactive disease had lower physical activity levels compared with controls [139] Sedentary time was positively correlated with disease duration and negatively with VO2 at RCP and VO2peak. Moreover, MVPA time was negatively associated with disease duration and timed-up-and-go score and positively associated with time-to-RCP, VO2 at RCP, time-to-exhaustion, VO2peak and current use of glucocorticoid [143]			
PWS	Youth with PWS spent 19.4% less time in weekly LPA and 29.8% less time in weekly VPA compared to controls [110]**.** Physical activity across intensity categories differed between study groups. Amount of activity was lower in all patients than controls and in non-ambulatory than ambulatory patients and controls, but similar between ambulatory patients and control [144]. The PWS group displayed lower PA and higher sedentary time compared to the control group [113]			MVPA and walking capacity increased after the programme without significant effect on body composition [113]
DMD	Strongest relationship between step activity and timed functional tests (particularly the 10-m walk/run) and moderate correlations with the 6 min walking distance, knee extensor peak torque, and plantar flexor peak torque. Moderate correlations between step activity and 10 m walk/run test, supine up, four stairs. Patients who were still ambulatory after 2 years demonstrated baseline step activity nearly double that of those who were no longer walking 2 years later [19]			
	QMT and accelerometry measures had a moderate or strong correlation, particularly indexed arm QMT with total wrist vector magnitude, total indexed QMT with total wrist vector magnitude and indexed leg QMT with total ankle vector magnitude [145]		DMD participants also demonstrated a progressive decrease in physical activity at 1- and 2-years for wrist accelerometry and at 2-years for ankle accelerometry (Killian et al. 2020)	
ALS	Results were associated with ALSFRS-R. The variation in vertical axis showed the strongest correlation [28]	Less variability comparing to the ALSFRS-R (co-efficient of variation 0.64–0.81 for inertial outcomes) [28]	Activity declined by 0.64% per month [28]	
	The accelerometer models achieved a median multiclass AUC of 0.73 on six limb-related functions. The correlations across functions observed in self-reported ALSFRS-R scores were preserved in ML-derived scores [146]			In the cohort of 54 test participants who received edaravone as part of their usual care, the ML-derived scores were consistent with the self-reported ALSFRS-R scores. At the individual level, the continuous ML-derived score can capture gradual changes that are absent in the integer ALSFRS-R scores [146]
Sarcoidosis	Daily PA and VO2max were lower in sarcoidosis patients than the known predicted values in healthy age-matched individuals. Sedentary time was positively correlated with disease duration and negatively with VO2peak. MVPA was negatively associated with disease duration, and positively associated with VO2peak, and current use of corticoids [101]			
*IDEEA, Intelligent Device for Energy Expenditure and Activity; MinisunLLC, Fresno, CA*				
CMT	Count of step climbing and sit to stand were lower in patients than in controls as well as mean daily step-climbing and walking velocities. Positive correlation between strength of the knee extensor muscles and both count of steps climbed and sit to stand [147]			
*Ipod*				
HD	Amplitude of thoracic and pelvic trunk movements was significantly greater in participants with HD. Individuals with HD demonstrated rapid movements with varying amplitudes that continuously increased without stabilizing [66]			
*Jamar Plus Digital; JLW Instruments*				
DM1	Grip strength demonstrated strong correlations with self-reported inventories of upper [38]			
*JiBuEn gait analysis system*				
SCA	There were significant differences in stride length, velocity, supporting-phase percentage, and swinging- phase percentage between the SCA group and the gait control group. Negative correlation between Velocity and ICARS and SARA scores. Correlation between Midsagittal relative vermis diameter and ICARS and SARA scores, as well as stride velocity variability [90]			
*Kinesia motion sensor*				
FD	Measures of head tremor are logarithmically related to Tremor Rating Assessment Scale [82]			Minimum detectable change (percent reduction) was approximately 66% of the baseline geometric mean. That is comparable to those previously reported for hand tremor [82]
*LabVIEW2011, National Instruments, Ireland*				
HD	Step time CoV was greater in manifest HD than controls, as was stride length CoV for late HD. Phase plot analysis identified differences between manifest HD and controls for SDB, Ratio∡ and Δangleβ DBS score was significantly associated with Ratio∡, SDB. UHDRS-TMS was significantly associated with Ratio ∡, SDB, Δangleβ and step time CoV, cadence CoV, stride length, and stride length CoV. Ratio∡ produced the strongest correlation with UHDRS-TMS [60]	There was no significant difference between tests for any measure (ICCs: speed 0.94, step time 0.89, step time CoV 0.67, cadence 0.72, cadence CoV 0.59, stride length 0.83 and stride length CoV 0.57) [60]		
*LEGSysTM, BioSensics, Newton, MA*				
SCA	Stride length variability, stride duration, cadence, stance phase, pelvis sway, and turn duration were different between SCA and controls. Sway and sway velocity of the ankle, hip, and center of mass differentiated SCA and controls. Stride length variability, stride duration, cadence, stance phase, pelvis sway, turn duration, sway and sway velocity of the ankle, hip, and center of mass showed moderate-to-strong correlation with SARA assessments of gait and stance and the BARS2 gait assessment [92]			
	The cycle detection technique showed an accuracy of 97.6% in a Bland–Altman analysis and a 94% accuracy in predicting the severity of the finger-to-nose test. Among the exctrated features, 22 showed an excellent correlation with finger-to-nose test and discriminated between SCA and control participants [148]	The results showed excellent intra-rater reliability as the ICC was in the range of 0.94–0.99 [148]		
*Locometrix®, Centaure Metrix, Evry, France*				
DM1	Patients displayed lower walking speed, stride frequency, stride length, gait regularity, and gait symmetry than controls. Strength of ankle plantar flexors, ankle dorsal flexors and neck flexors correlated with interstride regularity in the vertical direction. Knee extension strength correlated with gait symmetry in the anteroposterior direction. Center of pressure velocity was greater in patients and correlated with neck flexion and ankle plantar flexion weakness and with interstride regularity in the vertical direction [149]			
*MetaMotionR, mbientlabs, San Francisco, CA*				
ALS	Decreased stride length, increased stride duration and decreased walking speed were associated with lower functional walking scores, and the presence of a cane or walker [150]			
*Mimamori- GaitTM System; LSI Medience, Tokyo, Japan*				
PSP	Both PSP and PD patients shared the following similar hypokinetic gait characteristics: decreased velocity, step length, cadence and mean acceleration. Step time and variability in step time were mutually related. PSP patients showed characteristically low vertical displacement and a higher acceleration than PD patients at the same cadence [71]			
*Move 3 actigraphs (movisens GmbH; Karlsruhe, Germany)*				
TSC	Actigraph-measured movement was positively associated with ADHD and ASD symptoms. Higher ADHD symptoms and actigraph-measured movement levels were positively associated with ASD symptoms and negatively associated with IQ [126]			
*MOX Accelerometry; Maastricht Instruments BV, Maastricht, the Netherlands*				
DMD	Correlations with the PUL scale score were high for intensity and the total frequency of arm elevations per hour. Loderate correlation between number of transfers per hour and PUL scale score from low-middle and from middle-high. High correlation between the total number of transfers per hour and the PUL scale score [151]			
*Opal sensors, Mobility Lab, APDM, Portland, Oregon*				
PSP	The RF classifier allowed discrimination of PSP from PD with 86% sensitivity and 90% specificity, and PSP from HC with 90% sensitivity and 97% specificity [76]			
	Control subjects were able to change their postural strategy, whilst PSP and PD subjects persisted in use of an ankle strategy in all conditions. PD subjects had root mean square values similar to control subjects even without changing postural strategy appropriately, whereas PSP subjects showed much larger root mean square values than controls, resulting in several falls during the most challenging sensory organization test conditions [73]			
			A simple linear regression model incorporating the three features with the clearest progression pattern was able to detect statistically significant progression 3 months in advance of the clinical scores [77]	
DMD	T-test results show that, for all age groups, children of the same age with DMD and controls show significant differences in RCC [20]	ICC of all groups exceeded 0.8 [20]		
PWS	Children with Down syndrom and PWS exhibit reduced gait symmetry and higher accelerations at pelvis level than controls. While these accelerations are attenuated by about 40% at sternum level in controls and down syndrom, PWS children display significant smaller attenuations meaning reduced gait stability. Significant correlations between the estimated parameters and the GMFM-88 scale when considering the PWS group [152]			
ALS	The ability to extend the head backward and flex it laterally were the most compromised, with significantly lower angular velocity, reduced smoothness and greater presence of coupled movements with respect to the controls [32]	ICC was moderate to good in all movements and for most parameters [32]		
FSHD			For an average of 20.6 months, the iTUG duration stayed constant, whereas stride length, stride velocity, and trunk sagittal range of motion changed, indicating poorer performance. Arm swing changed in a compensatory direction toward the normative mean [153]	
	Gait parameters in FSHD participants were significantly altered compared with normative values. Stride velocity and trunk sagittal range of motion had moderate to strong correlations to other FSHD disease measures [154]	Reliability was excellent (ICC 0.84–0.99) [154]		
HD	Gait speed, stride length, lateral step variability, and stride length variability were consistently observed to be significantly different between the HD and control. HD participants demonstrated significantly greater dual-task cost for turning. Poorer performance on the SDMT and animal naming was significantly associated with increased gait variability. UHDRS-TMS were correlated with percent of time spent in swing phase, stride length CoV and percent of time spent in double support for all three conditions [57]			
	Postural sway and control differed between patients with HD and patients with premanifest HD. Selected postural measures had positive correlations with CAP scores and TMS [65]	All measures of interesthad good reliability (ICC 0.764–0.887), except Total Power AP for patients with premanifest HD that had moderate reliability (ICC: 0.644) [65]		
	Individuals with HD had greater APA acceleration amplitudes, smaller first step ROM and longer first step durations compared to controls. APA ML amplitude and duration under the no-load condition were significantly correlated. APA ML amplitudes under cognitive-load condition were significantly correlated with TFC and TMC. APA ML amplitudes under no-load condition were significantly correlated with TMS and SDMT. Anticipatory postural adjustment evaluation could predict gait speed [61]			
	The 90.5% of subjects was assigned to the right group after leave-one-subject–out cross validation and majority voting [68]			
	Individuals with HD had a greater increase in standing postural sway compared to controls from single to dual-tasks and with changes to support surface. Patients with HD showed a greater dual-task motor cost compared to controls [66]. Total sway, root mean square and mean velocity during sitting, as well as gait speed had the greatest importance in classifying disease status. Stepwise regression showed that root mean square during standing with feet apart significantly predicted clinical measure of chorea, and ordinal regression model showed that root mean square and total sway standing feet together significantly predicted clinical measure of tandem walking [61]			
FXS	FXS participants had reduced stride length and velocity, swing time, and peak turn velocity and greater double limb support time and number of steps to turn compared to controls under all conditions. Stride length variability was increased and cadence was reduced in FXS participants in the fast pace condition. There was greater dual task cost on peak turn velocity in men with FXTAS compared to controls [97]			
SCA	Larger variability of the swing period, toe-off angle and toe-out angle in pre-SCA2, and larger mean coronal and transverse ranges of motion of the trunk at the lumbar location and of the sagittal range of motion of the trunk at the sternum location compared to controls. During tandem gait, pre-SCA2 subjects showed larger lumbar, trunk, and arm ranges of motion than controls. The toe-off angle and swing time variability were significantly correlated with the time to ataxia onset, whereas the toe-off angle and transverse range of motion at trunk position during tandem gait were significantly associated with the SARA score [93]			
	Increased gait variability was the most discriminative gait feature of SCA; toe-out angle variability (sensitivity = 0.871; specificity = 0.896) and double-support time variability (sensitivity = 0.834; specificity = 0.865) were the most sensitive and specific measures. These variability measures were also significantly correlated with the scale for the assessment and rating of ataxia and disease duration. The same gait measures discriminated gait of people with prodromal SCA from the gait of controls [91]			
	Lateral velocity change (LVC) and outward acceleration but not general turning measures such as speed, allowed differentiating ataxic against healthy subjects in real life, with LVC also differentiating preataxic against healthy subjects. LVC was highly correlated with SARA score, and activity-specific balance confidence scale [89]			Moreover, LVC in real life allowed detecting significant longitudinal change in 1-year follow-up with high effect size [89]
CMT	Five mean gait outcomes measured, four showed statistically significant changes over the 6-min fast-as-possible walk: velocity, cadence, step time and trunk ROM. Stride length variability changed during the walking task, decreasing from bins 1–2, and remaining stable for bins 2–6. Changes in velocity, cadence, step time were related to general life satisfaction, but not perceived fatigue [36]			
*PAMSys-XTM sensors, BioSensics, Cambridge, MA*				
HD	In the clinic, the standard deviation of step time was increased in HD compared to controls. At home, significant differences were observed in seven additional gait measures (cadence, maximum step peak acceleration, maximum medial–lateral velocity, maximum medial–lateral displacement, average step peak acceleration, average medial–lateral velocity and displacement). The gait of individuals with higher TMS differed significantly from those with lower TMS on multiple measures at home [50]			
*Philips Respironics, Bend, OR*				
NP-C	Significant correlations were demonstrated between BK25, BK50 and BK75. FDS correlated with PDQ, UPDRS IV, UPDRS and AIMS. DK25 in comparison with NUCOG-A and DK75 in comparison with NUCOG and NUCOG-A demonstrated significant correlations. Additionally, duration of illness in comparison with PTI demonstrated significance [120]			
*Philips Respironics, Bend, OR*				
SBMA				Patients with higher AMAT subscore increased total activity count comparing to controls after intervention [45]
*RehaGait (HAS-OMED, Magdeburg, Germany)*				
HD				Device-extracted parameters revealed significant improvement in area, velocity, acceleration and jerkiness of sway in cerebellar repetitive Transcranial Magnetic Stimulation versus sham stimulation [74]
*SAM (Modus Health LLC, Washington, DC, USA)*				
Rett syndrome		Repeatability of step-count pairs was excellent (ICC 0.91, 95%). The standard error of measurement was 6 steps/min and we would be 95% confident that a change more than 17 steps/min would be greater than within-subject measurement error [125]		
				After intervention, the sedentary time was decreased. Positive effects with small to medium effect sizes were seen in sedentary time and that was maintained during follow-up period [122]
*SenseWear Armband, BodyMedia, Inc., Pitts burgh, PA, USA*				
Sarcoidosis	There was a significant correlation with SGRQ score, SF-12 physical health, Physical fatigue and reduced activity MFIS subscores, 6MWD [103]			
Scleroderma	Exercise capacity during daily activity was reduced compared with controls, and was associated with early evidence of functional decay [107]			
CMT	Results showed a decrease in daily steps taken in the CMT group, but shorter bouts of sedentary activity and more frequent transitions from sedentary to active behaviors compared to controls [155]			
*SHIMMER sensors, Shimmer Research Ltd., Dublin, Ireland*				
HD	Stride length and gait velocity were reduced, while stride and stance time were increased in patients with HD. Parameters reflecting gait variability were substantially altered in HD patients (stride length CV and stride time CV). Parameters representing gait variability (stride time CV, stance time CV, swing time CV, stride length CV, gait velocity CV) showed moderate to strong correlations to UHDRS-TMS. Stride length and gait velocity showed moderate inverse correlations to UHDRS-TMS [156]			
PSP	Gait speed was significantly reduced in patients with PD compared to controls and even more in atypical PD. Similar results were obtained for stride length. The maximum toe clearance and heel strike angle, toe off angles were significantly impaired in PD and atypical PD patients compared to controls but did not reveal a significant difference between both patient cohorts. Clinical ratings significantly correlated with gait speed and stride length [72]			
HSP	There were significant associations of absolute stride parameters with single SPRS items reflecting impaired mobility, with patients’ quality of life, and notably with disease duration. Sensor-derived coefficients of variation, on the other hand, were associated with patient-reported fear of falling and cognitive impairment [96]		In a small 1-year follow-up analysis of patients with complicated HSP and fast progression, the absolute values of mobile gait parameters had significantly worsened compared with baseline. [96]	
*SilmeeTM Bar-type Light, manufactured by Toshiba Corporation, Tokyo, Japan)*				
DMD	The Cj values had significant and very strong or strong correlations with the Brooke Upper Extremity Scale and the arm function scores for the DMD Functional Ability Self-Assessment Tool. The values also had a very strong or strong correlation with the elbow flexion strength [157]			
*Smartwatch*				
HD	There was a significant correlation between the model chorea score and the patient-reported chorea score for the same assessment [46]			
*Step WatchTM Activity monitor (SAM), Cyma Inc. Seattle, USA*				
DMD	Significant correlations for 10-m walk/run versus high and low stride rates were found at baseline. Changes in strides/day and percentages of high frequency and low frequency strides correlated significantly with changes in 10-m walk/run speed [158]		There were significant declines in average strides/day and percent strides at moderate, high and pediatric high rates as a function of age. Step activity outcomes were sensitive to change over 1 year, but the direction and parameter differed by age group [158]	
DMD	StepWatch accelerometry identified a decreased capacity for ambulation in boys with Duchenne compared to healthy controls. There were strong, significant correlations between 6-min walk distance and all StepWatch parameters [23]			
SCA	The objective monitor measurements were highly associated with disease duration and with the functional stage of disease. Monitor measurements were significantly correlated to SARA scores with the exception of the percent of steps expended in moderate and high speeds of activity. Fewer monitor measures had significant correlations with walk and peg board scores [159]	The objective SAM outputs also possessed high internal consistency, high ICC and could be fitted to a single factor by factor analysis (Subramony et al. 2012)		
CMT	Minutes at low activity average, step at low and high activity average, peak activity index average was correlate with myometer measures. Minutes high activity average correlate with myometer measures and the Short Form 36 Physical Composite Score. Some Sustained Activity measures correlated with CMT Examination Score, myometer measures and Short Form 36 Physical Composite Score [128]	Test–retest for both the 6MWT and SAM demonstrated excellent reliability with a value > 0.90 obtained comparing the two evaluations [128]	Statistical analysis showed a worsening of the StepWatchTM Activity Monitor outputs [129]	
*Roche HD Digital Monitoring*				
HD	Good overall convergent validity of sensor-derived features to Unified HD Rating Scale outcomes and good overall known-groups validity among controls, premanifest, and manifest participants were observed. [55]	All sensor-based features showed good to excellent test–retest reliability (ICC 0.89–0.98) [55]		
*wGT3X-BT, Timik Medical, Herlev, Denmark*				
DM1	The individuals with DM1 were less physically active compared to healthy controls [160]			

*DMD* Duchenne muscular dystrophy, *BBT* block and box test, *ICC* intraclass correlation coefficient, *SMA* spinal muscular atrophy, *MFM* motor function measure, *FSHD* facioscapulohumeral dystrophy, *MMT* manual muscle testing, *HD* Huntington disease, *FRDA* Friedrich Ataxia, *FD* Focal Dystinia, *PD* Parkinson disease, *UHDRS* Unified Huntington's Disease Rating Scale, *TMS* total motor score, *MPS* mucopolysaccharidosis, *TWSTRS* Toronto Western Spasmodic Torticollis Rating Scale, *MG* myasthenia gravis, *PA* physical activity, *MVPA* moderate-to vigorous physical activity, *PAL* physical activity level, *ALS* amyotrophic lateral sclerosis, *ALSFRS-R* Revised Amyotrophic Lateral Sclerosis Functional Rating Scale, *DM* myotonic dystrophy, *PWS* Prader–Willi syndrome, *HR* harmonic ratio, *LPA* light physical activity, *MPA* moderate physical activity, *VPA* vigourous physical activity, *QMT* quantitative muscle testing, *CMT* Charcot-Marie-Tooth, *SCA* spinocerebellar ataxia, *ICARS* International Cooperative Ataxia Rating Scale, *SARA* Scale for the Assessment and Rating of Ataxia, *BARS2* Brief Ataxia Rating Scale, *PSP* progressive supranuclear palsy, *TSC* tuberous sclerosis complex, *ADHD* Attention deficit hyperactivity disorder, *ASD* autism spectrum disorder, *IQ* intelligence quotient, *CoV* coefficient of variation, *PUL* performance of upper limb, *GMFM* gross motor function measure, *TUG* timed up and go test, *SDMT* symbol digit modalities test, *CAP* CAG-age-product, Total Functional Capacity Score, *FXS* Fragile X Syndrome, *NP-C* Niemann–Pick type C, *UPDRS* Unified Parkinson's Disease Rating Scale, *AIMS* Abnormal Involuntary Movement Scale, *NUCOG* neuropsychiatry unit cognitive assessment tool, *CV* coefficient of variation, *HSP* hereditary spastic paraplegia, *SPRS* Spastic Paraplegia Rating Scale

### Spectrum of pathological conditions assessed

#### Neuromuscular disorders

##### Duchenne muscular dystrophy (DMD)

Among the 18 studies that involved subjects with DMD, two studies also included subjects with Becker muscular dystrophy [17] and Niemann–Pick type C [18]; the rest involved only subjects with DMD. One study showed that participants who were ambulatory 2 years after study initiation had almost double the level of step activity at baseline compared to subjects who were not ambulatory after 2 years [19]. Two studies demonstrated that limb coordination (homolateral-limb coupling coefficient) and physical activity (stride count and time spent in different levels of activity) declined with age [20]. Another study showed that total physical activity did not significantly change over 1 year [17]. Validity was demonstrated for numerous gait parameters (e.g., stride length, stride velocity) [21]. One outcome, stride velocity 95th centile (SV95C), which was qualified as a secondary outcome measure [22], was in public consultation for qualification as a primary endpoint until April 2023 and is very likely to be qualified in 2023. Of the two interventional studies identified, one, a randomized controlled trial, failed to show the effect of a nutritional supplement on steps or inactive minutes per day [23], and the other showed that duration of walking episodes or the succession of walking episodes increased with prednisolone treatment [24]. One research team used a smartphone maze game to evaluate and improve upper limb performance [25]. In another study, the feasibility of a patient-led initiative to assess upper and lower limb function through video analysis of four motor tasks was demonstrated [26].

##### Amyotrophic lateral sclerosis (ALS)

We found 15 studies that used various sensors, mainly inertial technologies, in the ALS population. One study showed an inverse association between overall acceleration average and ALS risk based on genome-wide association study and inertial data [27]. Raw parameters as vector magnitude count and variation in vertical axis showed less variability than clinical assessment leading to a potential reduction of sample sizes by 30.3% for 12-month trials [28]. Another study showed that daily home measurements such as step count, electrical impedance myography, and grip strength resulted in more accurate assessment to track progression and could reduce sample sizes for trials [29]. Two studies used apps that integrated different tasks to monitor motor control in the upper body, speech, and cognition. One of these apps detected two metrics, error metrics and velocity rate, that are useful for inference of clinical variables [30]. Changes in typing activity (e.g., acceleration at key press, key release) were correlated with progression of dysfunction [31]. Assessment of head movements through four parameters confirmed the efficacy of the Head Up collar by showing a significant improvement in the control of movement expressed as the median ratio of movement coupling value [32]. Another study showed that it is feasible to assess ALSFRS-R, a score that stratifies severity of ALS, remotely through an app [33]. Studies have also demonstrated that accelerometers and electromyography can be used for evaluation of tremor frequency, involuntary movement [34], and fasciculation [35].

##### Charcot-Marie-Tooth (CMT)

Six studies were found that used inertial sensors in subjects with CMT disease, four employed the sensors in the home setting. Four studies collected information on physical activity based on various variables (e.g., power of different activities, steps taken) without any ranking in terms of importance of these variables. One study attempted to characterize gait fatigability and demonstrated that velocity, cadence, trunk range of motion, step time, and stride length variability showed statistically significant differences during the 6-min walk test [36]. One study used the detection to tremor characteristics (e.g., frequency, spectral power) to study possible mechanisms for tremor [37].

##### Myotonic dystrophy type 1 (DM1)

Four studies focused on difference between in the myotonic dystrophy patients and controls mainly in terms of active minutes, limb accelerations, and gait abnormalities (i.e., walking speed, stride frequency, stride length). Three used inertial technology. The fourth showed the feasibility of remote video assessment of timed-up-and-go and a digitally measured hand grip strength [38].

##### Facioscapulohumeral muscular dystrophy (FSHD)

Four studies in subjects with FSHD focused on psychometric evaluation of outcome measures gained with inertial sensors. In one of them, data were acquired for 6 weeks through multiple technologies (accelerometer, apps, GPS, Google Places calls, microphone), and analyses resulted in an accurate classifier [39].

##### Myasthenia gravis (MG)

Two studies of subjects with myasthenia gravis continuously assessed for 7 days, examined the validity of physical activity variables, defined as total [40] or levels of physical activity (e.g., low, moderate, high) [41].

##### Spinal muscular atrophy (SMA) types 2 and 3

In a natural history study of subjects with SMA2 and SMA3, wrist angular velocity, wrist acceleration, wrist vertical acceleration, power, and percentage of active time were extracted from a magneto-inertial sensor worn at the wrist [42]. The reported results were similar to those described in DMD [43]. In a limited number of ambulant patients, the device was placed on the lower limb and the SV95C, which was qualified in DMD [22] and studied in other dystrophies [44], remained stable over 12 months.

##### Spinal and bulbar muscular atrophy (SBMA)

The single study of subjects with SBMA assessed the use of accelerometry to identify changes in the physical activity of patients after 10 days of functional exercises [45].

#### Movement disorders

##### Huntington disease (HD)

Among the 26 studies on HD patients, 10 reported continuous [46–51] or discontinuous [52–55] real-life assessments. Physical activity was assessed with different sets of variables in each study (e.g., activity profiles, step count, and time spent sitting). Contradictory results were obtained regarding discriminant validity of levels of physical activity [47, 51]. Two studies found no difference in physical activity between patients and controls, but there were differences in spatiotemporal gait variables [50, 56]. Validity and accuracy of estimating gait events and various gait spatiotemporal parameters were assessed in controlled environments [50, 56–63] and in real-life settings in several studies [49, 50, 55, 56]. These studies used a selection of outcome measures (e.g., cadence, stride length, gait speed, variability) with no prioritization of outcomes in terms of metric properties. In a cross-sectional study, compliance and psychometric properties of upper and lower limb variables (e.g., sway path, spiral drawing speed variability, median turn speed, and step frequency variance) were extracted from a digital monitoring platform [55]. Balance and body sway (as root mean square [61, 64], jerk, sway area and power [65], peak and mean thoracic and pelvic excursion [66]) were impaired even at the premanifest stage of disease.

Upper limb function was evaluated in three studies in real-life settings using software to record speed of finger movement during computer typing [53], inertial technology to compute a chorea score, and a smartphone touchscreen sensor to assess the tap rate [53]. One study demonstrated the validity of a model chorea score based on accelerometer data [46]. Three studies used machine learning approaches to extract composite scores [67] (i.e., movement impairment scores) as well as complex [68] and standard [59] spatiotemporal variables (e.g., stride length, velocity) for discriminant validity analysis. Use of machine learning based on inertial data, the accuracy of gait event detection [63], the ability to predict upper limb impaired reaction (reaction time and error distance) [69], and the potential of root mean square to serve as biomarkers of postural control impairments [61] were also demonstrated in a number of studies. Three studies demonstrated that compliance was good [54, 70] and reported that participants would be willing to wear the sensors again [47].

##### Progressive supranuclear palsy (PSP)

Seven studies focused on evaluation of subjects with PSP using inertial sensors in a clinical setting. Validity assessment via gait length or speed comparison between subjects with PSP and those with Parkinson’s disease (PD) were not in agreement [71, 72]. Patients with PSP have significant impairment in postural control, expressed as root mean square, which can result in falls during challenging test conditions [73]. Cerebellar repetitive transcranial magnetic stimulation had a significant effect on inertial parameters correlated with stability (i.e., area, velocity, velocity, acceleration, and jerk in the medio-lateral direction) compared to placebo [74]. Upper limb assessment was restricted to the discriminant validity assessment of finger tapping-specific features [75]. Another study reported that a machine learning approach identified classifiers that discriminate PSP from PD with high sensitivity and specificity [76]. In the only longitudinal study of PSP subjects that we found, three out of 150 gait and postural features that were the most sensitive to change (i.e., mean toe off angle, mean turn velocity, standard deviation of stride length) were integrated in a regression model able to detect early progression [77].

##### Focal dystonia (FD)

We found nine studies regarding hand dystonia [78–81] or cervical dystonia [82–86]. One study showed that instrumental detection of dystonic tremor outperforms clinical examination [80]. Two studies aimed to define the characteristics of dystonia [79] and dystonia-associated tremor [78] with multiple measures including force, power, and frequency. In the latter study, the authors built an accurate classifier (95.1%) that discriminated between essential tremor, dystonic tremor, and controls using two tri-axial accelerometers and four pairs of surface EMG electrodes. One study used simultaneous MRI and accelerometer recording of tremor power to investigate its pathophysiology [81]. In a single-participant interventional study, tremor magnitude assessed by 3D-kinematics was concordant with accelerometry and improved with deep brain stimulation [86].

Accelerations in three directions and speed measured with an inertial device during a Timed Up and Go Test showed abnormalities in turning, standing-up, and sitting [83].

#### Genetic syndromes with manifestations of ataxia

##### Spinocerebellar ataxias (SCA)

We found nine studies in subjects with SCA that used inertial technologies. Evaluations in clinical settings demonstrated that wearable sensors are able to accurately capture gait parameters (i.e., mean step velocity, length, and swing and stance time) [87] and turn (i.e., angle, duration, steps, mean velocity, lateral velocity change, outward acceleration, inward acceleration, average and maximum rate, and hesitations) [88, 89] in patients with SCA. Gait parameters and body sway measures (i.e., stride length, its variability, stride duration and speed, cadence, and swing, and stance time or percentage, double-support time variability, pelvis, ankle, and hip sway, turn duration, lateral velocity change, outward acceleration, and toe-out angle variability) consistently identified ataxic gait changes in the clinical setting [87, 89–92]. Inertial technology also detected gait abnormalities as variability of the swing period, toe-off and toe-out angles, and elevation of feet at mid-swing as well as ranges of motion of the trunk and arm in patients with premanifest SCA type 2 [91, 93].

##### Hereditary spastic paraplegia (HSP)

We found three studies led by the same team of researchers that studied subjects with hereditary spastic paraplegia using inertial sensors fixed on the feet. Two of them, a pilot study and its validation [94, 95], validated a machine learning approach against a GAITRite system and manual sensor data labeling. The third study used the same approach on a large transversal cohort (n = 112) and a small 1-year longitudinal cohort (n = 11) [96].

##### Fragile X Syndrome (FXS)

In two studies in patients with FXS, investigators performed an inertial detection of stride length and velocity, swing time, peak turn velocity, double limb support time, and number of steps to turn. A fast-paced gait exacerbated gait deficits, and stride velocity variability when gait was fast paced was significantly associated with the number of self-reported falls in the past year [97]. Further, cognitive performance was significantly associated with shorter stride length slower turn-to-sit times in premanifest patients [98].

##### Friedreich’s ataxia (FRDA)

We found two studies of patients with FRDA that used inertial technology. Remote monitoring of several voice parameters, upper limb function through 14 parameters that can be grouped into parameters related to movement velocity, spectral frequency, and parameters related to deviation of the ideal trajectory, and 15 spatiotemporal gait parameters was feasible over 1 week [99]. The sensitivity of an upper limb composite score, the AIM-S, obtained through sensors contained in a spoon designed to detect deterioration in upper limb function, was greater than other measures [100].

#### Multisystemic rheumatological diseases

Five studies in subjects with sarcoidosis reported transversal evaluation of physical activity expressed in various way as number of steps [101–104]*,* sit-to-stand transition [102], or acceleration per day [105] in real-life settings over seven to 14 days. Patient-reported physical activity and fatigue collected through an app correlated with smartphone-tracked physical activity [104]. In dermatomyositis, three studies focused on real-life transversal physical activity assessment for 7–8 days. One study used vigorous physical activity detection for the validation of the stage of exercise scale in several rheumatic diseases [106]. The single study on scleroderma, which used an accelerometer for a 6-day period, explored validity of physical activity level detection [107].

#### Miscellaneous diseases

##### Prader–Willi syndrome (PWS)

Patients with PWS were included in eight different studies using accelerometers and/or gyroscopes. One study simply demonstrated significant agreement between mean stride length, mean stance percentage, and stance percentage coefficients of variation measured during two different walking tasks [108]. One study showed that because of the poor gait symmetry in PWS, upper body accelerations and the harmonic ratio (i.e., measure of step-to-step symmetry based on trunk acceleration) can be used as innovative parameters for gait analysis, providing information that cannot be extracted from spatiotemporal parameters only [109]. Two studies showed that patients with PWS do not meet healthy physical activity recommendations [110, 111]. Another group showed that moderate physical activity accounts for the variability found in bone mineral content and density and its z-score [112]. Two studies assessed the effect of training exercise programs. One reported that training increases moderate‐to‐vigorous physical activity and walking capacity without effect on body composition [113], whereas the other found that training did not result in changes in moderate‐to‐vigorous physical activity but did cause improvements in body coordination, strength, and agility [114].

##### Metabolic diseases

One study, in subjects with Pompe disease, focused on concurrent validity of physical activity outcomes (step count and peak 1-min activity) [115]. Another study showed that measurements performed by patients at home with a handheld electrical impedance device did not differ significantly from those performed in the clinic setting. These measurements correlated with measures of muscle strength and function and quantitative muscle ultrasonography [116]. Two trials compared physical activity and rhythmicity of children with mucopolysaccharidosis type III and Fabry disease to controls using continuous inertial recording [117, 118]. One study of patients with GM2 gangliosidosis used both a wearable device and a smartphone application and focused on compliance. This study failed to show significant changes in average daily maximum, average daily steps, or average daily steps per epoch over 6 months [119]. We found one study that assessed the feasibility of the use of accelerometers on patients with Niemann–Pick type C, DMD, and with juvenile idiopathic arthritis using the same outcome measures as used in the study of patients with GM2 gangliosidosis [18]. In another study, variables used in similar studies in PD (i.e., bradykinesia, dyskinesia, fluctuation score, percentage time immobile, and percent time with tremors) were used, demonstrating that bradykinesia and percentage time immobile are features of Niemann–Pick type C [120].

##### Rett syndrome

Of the five studies found that reported studies of subjects with Rett syndrome, all but one were observational, and no study had controls. Four studies used the same three inertial devices [121–124], and one study compared these sensors [125]. In a real-life setting, patients with Rett syndrome have sedentary behavior based on a 4-day accelerometer recording on the ankle [121]. One study was interventional and assessed the effects of an interventional program that aimed to increase enjoyable activities; positive effects on sedentary time, daily step count, and walking capacity were observed after the intervention [122]. Using machine learning algorithms and data derived from heart rate variability and activity metrics, a research team built an accurate classifier between high and low-severity Rett patients [123].

##### Tuberous sclerosis complex (TSC)

We identified a single study in this patient population that used accelerometry to assess movement levels in controlled settings and correlated these with clinical parameters [126].

##### Narcolepsy

Our review identified one study that compared energy expenditure and outcomes of physical activity (e.g., metabolic equivalent of task, step count, total energy expenditure), between a group of participants with narcolepsy type 1 and a group of participants with narcolepsy type 2 or idiopathic narcolepsy [127]. A tri-axial accelerometer was used, and there were no significant differences between the two groups that could account for weight gain.

#### Quality assessment

Finding a quality assessment tool suitable to the type of studies included in this review was challenging. The quality of studies we found were generally good even if data needed to answer all items of the quality assessment questionnaire were missing for most studies. The results of the quality assessment are presented in Additional file 3.

## Discussion

This review presents the developments in the field of digital outcome measures for a range of rare diseases with neurological manifestations. The increase in the number of studies on this topic attest of the growing interest in the field: In 2011, there were two published studies that included digital outcome measures, whereas in 2022 there were 25. A key finding of this review is that the use of digital outcome measures for motor function outside the clinical setting is feasible and it is being employed to evaluate subjects with a broad range of diseases. Although this is very encouraging, a publication bias cannot be excluded, as studies that failed to recruit participants or to collect robust data are less likely to be published.

An asset of technologies that provide digital measures of motor function is their potential application to the home setting, which is even more appealing since the COVID-19 pandemic. Although these devices are meant to be used at home, more than half of the evaluations in the studies took place in clinical setting. This indicates that many of these technologies are not yet ready to be used unsupervised. This could partially explain why the use of such outcomes for interventional studies remains uncommon (n = 14) and limited to a small number of diseases (i.e., PWS, Rett syndrome, FSHD, DMD, SBMA, ALS, PSP, FD, and HD). Future research should focus only on sensors that are meant to be used outside clinical environment, unless there is a clear plan for technology evolution toward that goal or if the purpose of the device is not remote monitoring (e.g. a device aiming through digitalization to remove part of the subjectivity of standard in-clinic assessments).

Another key finding is that most studies investigated a number of potential outcomes, mostly to demonstrate the validity of the technology via group comparisons and correlations with gold standard outcomes, whereas other psychometric properties were largely neglected. It is generally assumed that digital outcomes will be more sensitive to change in patient condition than standard tests and scales; however, this has yet to be proven, as suggested by the small number of studies reporting longitudinal data (n = 34) or sensitivity to positive or negative change (n = 21). A few research teams have performed psychometric evaluations, in CMT [128, 129], ALS [28], FSHD [44], and FRDA [130], regarding the use of accelerometers for physical activity assessment, but the psychometric evaluations were not complete.

Despite the number of studies, we found that few outcome measures had been robustly studied and adopted as secondary or primary endpoints in clinical trials. An exception is the recent qualification of the SV95C in DMD [131]. SV95C has been shown to be discriminative and to have concurrent validity, reliability, and clinical relevance as assessed through interview with patients, precise context of use, and sensitivity to positive and negative change. Normative data have been collected [132]. As stated on the FDA website [5], the collection of information to formally qualified a clinical outcome assessment requires a long-term commitment. SV95C has been used in evaluation of subjects with limb-girdle muscular dystrophies, FSHD, and SMA, but how the qualification in DMD can be extended to less common diseases remains to be determined. This review did not identify other digital outcomes for which available metric properties description could complete the qualification process.

Outside diseases like DMD or Rett that are nearly only observed in males or female, respectively, very few studies paid specific attention to balance gender representation in the studied population- which may question the generalizability of the conclusion. Nevertheless, gender may clearly influence the phenotype of some autosomal condition such as SMA type 3. In this view, balancing the sample according to gender rather than to phenotype or phenotype/age can potentially result towards a bias towards specific- and more specifically milder- phenotypes.

Of the 139 relevant papers, the vast majority used wearable sensors and fewer studies used portable sensors or non-wearable/portable technologies. The most common sensor is magneto-inertial technology (used in 126/139 studies). This technology is at a more mature stage than other types of sensors. As shown in Fig. 2, most studies reported the use of sensors that were attached on the trunk or the back, which allow for gross evaluation of motor function and ambulation. In contrast, sensors fixed at the extremities allow for refined and more precise analysis of movement. Physical activity was the most frequently assessed parameter (n = 58), followed by gait (n = 39). This is likely because ambulation is critical from patient, caregiver and and clinician perspectives. Technical reasons could also contribute as gait is easier to record digitally than movements that are more erratic. Development and validation of outcomes for upper limb are one step behind. Given the heterogenicity of variables, there appears to be no consensus on an efficient way to quantify upper limb motor function or activity. Quantification of abnormal movements such as tremor may be slightly easier to harmonize.

Although promising, machine learning approach was used in few studies; it was employed almost exclusively in studies of subjects with HD [59, 61, 63, 67–69]. These studies demonstrated that machine learning algorithms can distinguish between normal and pathological motor performance. In rare diseases, machine learning faces the issue of the paucity of data. A major obstacle is the difficulty associated with gathering clinically valid information through interviews with patients, as the outcomes that result from machine learning are often non-intuitive. The solution resides in a clear association with a hard disease milestone or a clinically significant event, but this requires large amounts of data of high quality. Recent papers in DMD [14] and Friedreich’s ataxia [133] showed the feasibility and the potential of machine learning, but these proof of mechanism study were conducted using different sensors in a controlled setting [14].

Although we used a pre-defined search strategy, our search had several limitations. Four papers from 2021 were not accessible in their full versions, and we had to discard them. Other limitations could be related to methodological reasons (e.g., we included only manuscript published in English, did not search grey literature, and only included references cited by selected papers). For this review, we excluded studies using technology-based devices with no potential to be used outside the hospital setting. Examples of excluded articles include studies using laboratory motion analysis such as hip kinetic and spatio-temporal gait parameters in boys with DMD [134, 135] and Kinect-based stereo camera assessments that have been used in studies of subjects with ALS [136] and FSHD [137]. Motion analysis is a robust alternative to standard clinical evaluation and has the potential to be used as a complementary approach to remote movement monitoring even at home setting.

## Conclusion

This review highlights several issues limiting the full integration of digital outcome measures into clinical trials and practice. Despite promising initiatives, most studies included in this review are monocentric, and almost all studies were performed in small groups of subjects. Another shortcoming is that it is difficult to compare data between studies, as different manufacturers provide different algorithms to analyze the recorded data.

Future research should focus on the systematic validation leading to the qualification of devices, variables, and algorithms to allow remote evaluation of diseases. To address the issue of generalizability, open-source platforms that facilitate data collection, sharing, and interpretation are necessary. The qualification of a first digital outcome could pave the way to development of digital outcome measures. It is clear from the current state of the field that digital outcome measures have great potential to positively impact clinical trials and accelerate drug development processes.

## Supplementary Information

**Additional file 1.** Method.

**Additional file 2.** Data extracted from individual studies.

**Additional file 3.** Results of quality assessment.

## Data Availability

Data sharing is not applicable to this article as no datasets were generated or analysed for the current manuscript.
