# Supplementary material for: The use of digital outcome measures in clinical trials in rare neurological diseases: a systematic literature review
Source: Orphanet J Rare Dis. 2023 Aug 2;18:224. doi: 10.1186/s13023-023-02813-3 (PMC10398976; doi:10.1186/s13023-023-02813-3)
Supplement: Supplementary file 1 — Additional file 1. Method. [file 13023_2023_2813_MOESM1_ESM.docx]

**Additional file 1: Methods**

Before formulating the search strategy, the lead authors (M, TM) created independently a selection of all rare diseases with neurological manifestation using the list:

<https://ec.europa.eu/health/archive/ph_threats/non_com/docs/rdnumbers.pdf> (last accessed on January 25^th^, 2023).

Any disagreement between the reviewers was resolved with the assistance of a third reviewer (LS). We included only rare diseases with a prevalence equal or higher than 1/100,000 and we excluded general terms (e.g., autism spectrum diseases).

Then, we conducted a review of the literature using a comprehensive search of 2 databases (MEDLINE via Ovid and EMBASE electronic databases) and pre-defined selection criteria. Our search strategy intended to capture studies using a technology-based device with potential for remote monitoring of the muscle strength and motor function of humans affected by rare neurological diseases. The literature search and analysis were conducted in accordance with the Preferred Reporting Items for Systematic Reviews and Meta-Analysis statement (PRISMA)^15^. The search strategy is explained in our published protocol, available at the International Prospective Register of Systematic Reviews (Registration: CRD42021281236).

We search Medline via Ovid and Embase on June 1^st^, 2021 and we last updated our search strategy on the November 30^th^, 2022. We included all studies with published results in the last 10 years (from June 1^st^, 2011 to November 30^th^ 2022) in English. After running the search strategy, citations of all the selected papers will be uploaded into Mendeley. Duplicates were manually eliminated.

Two independent reviewers (MP, TM) without conflict of interest reviewed the titles and abstracts of all potentially eligible articles. Any disagreement between the reviewers was resolved with the assistance of a third reviewer (LS).

All previously selected articles were fully reviewed by two investigators to decide if they met the eligibility criteria (available at the International Prospective Register of Systematic Reviews). The two reviewers compared their findings and recorded the reason for exclusion. Potential disagreements were resolved by consensus or, if necessary, with the involvement of a third investigator (LS).

Study characteristics related to publication (authors, title, year of publication) and study design (population characteristics, devices, recorded parameters, experimental set up, main findings) were extracted. One assessor (MP) extracted and digitally registered this information using Microsoft Office Excel. Another assessor (TM) verified the extracted data from all included articles. Selected papers were clustered according to the disease.

We performed risk of bias assessment of the included studies, using the Mixed Methods Appraisal Tool (MMAT).
