# Supplementary material for: The use of digital outcome measures in clinical trials in rare neurological diseases: a systematic literature review
Source: Orphanet J Rare Dis. 2023 Aug 2;18:224. doi: 10.1186/s13023-023-02813-3 (PMC10398976; doi:10.1186/s13023-023-02813-3)
Supplement: Supplementary file 2 — Additional file 2. Data extracted from individual studies. [file 13023_2023_2813_MOESM2_ESM.docx]

**Additional file 2**

| Reference | Disease | Aim of the study | Number of patients (women) | Age : mean ± SD (years) | Controls : number (women) | Age : mean ± SD (years) | Participants with another disease : number (women) | Age : mean ± SD (years) | Device : number, type (brand), (if applicable). | Number and position of sensors (if applicable) | Outcome measures | Experimental set up : controlled environnement or daily living, duration, task | Main findings |
| --- | --- | --- | --- | --- | --- | --- | --- | --- | --- | --- | --- | --- | --- |
| Le Moing, et al.  PLoS One, 2016 | Duchenne Muscular Dystrophy (DMD) | To determine relevant and pertinent clinical variables with potential for use as outcome measures in clinical trials or to guide therapy decisions | 7 | 18.5 ± 5.5 |  |  |  |  | Three-axis accelerometer, a three-axis gyroscope, and a three-axis magnetometer (Actimyo, Sysnav, Vernon) | 2 on each wrist | Four variables representative of upper limb activity were studied: rotation rate, ratio of the vertical component in the overall acceleration, hand elevation rate and an estimate of the power of the upper limb | Patients were assessed while wearing the device during performance of validated tasks (MoviPlate, Box and Block test and Minnesota test) and tasks mimicking daily living. | All ActiMyo variables showed high to very high reliability as assessed using ICC values. For the MoviPlate and Box and Block test, all variables were significantly correlated with the functional scores. norm of the angular velocity, power and elevation rate were significantly correlated with the Minnesota scores and with the writing task. The mean of the rotation rate and mean of the elevation rate appeared promising since these variables had the best reliability scores and correlations with task scores. Parameters could be computed even in a patient with a Brooke functional score of 6. |
| Fujii, et al. Brain Dev., 2019 | Duchenne Muscular Dystrophy (DMD) | To evaluate the utility and reliability of accelerometric analysis of motor activity in nonambulatory patients with DMD | 7 | 20 |  |  |  |  | Tri-axial accelerometer (SilmeeTM Bar-type Light, manufactured by Toshiba Corporation, Tokyo, Japan) | 1 on the dorsal face of the dominant forearm near the wrist | Cumulative sum of jerks (Cj) defined as the total sum of the jerk values obtained at a measurement frequency of 15.625 Hz for 8 h. The root of the sum of squared values of the changes per unit time in the 3 axes of the accelerometer was defined as a jerk. | The parents and the patients were instructed on how to use this accelerometer, and the equipment was attached and switched on at home on a weekday morning before leaving for school or work. Measurements were taken for at least for 8 h. Patients were also assessed with the Brooke Upper Extremity Scale, the DMDSAT and dynamometer measures. | The Cj values had significant and very strong or strong correlations with the Brooke Upper Extremity Scale (p = 0.00023) and the arm function scores for the DMD Functional Ability Self-Assessment Tool (p = 0.027). The values also had a very strong or strong correlation with the elbow flexion strength (nondominant arm: p = 0.002; dominant arm: p = 0.052). |
| Ganea, et al.  J. Child Neurol., 2012 | Duchenne Muscular Dystrophy (DMD) | To investigate the alteration of the gait pattern in children with DMD, using body-worn inertial sensors during a long walking distance | 25 | 8 ± 1.9 | 20 | 7.8 ± 2.5 |  |  | 1/ Gyroscope (ASUR devices) 2/ Tri-axial accelerometer (Physilog device) | 1/ 2 on the shanks 2/ 1 on the trunk | 1/ Spatio-temporal parameters : stride length, shank peak angular velocity, stride velocity, cadence, double support 2/ coefficient of variation (CV) corresponding to the percentage ratio of standard deviation (SD) of a parameter and its average (mean) during the walking trial  3/ the smoothness of the trunk movement was assessed based on the spectral entropy of the acceleration norm. | Participants were asked to walk over 200 m at a self-determined normal pace. The test was indoors, along a long, flat, straight, enclosed corridor, with a hard surface. The walking course was approximately 100 m. Participants were asked to turn around at the end of the corridor and walk back. | Except cadence, all gait parameters showed significant differences between patients and healthy children. They found a large statistically significant effect size, for all parameters, except shank peak angular velocity, cadence, double support, stride length variability, and cadence variability. When the group of patients was divided into mild and moderate based on the Motor Function Measure, shows that all gait parameters were more affected in the moderate group. A large statistically significant effect size was found for cadence, stride velocity, and spectral entropy. |
| Davidson, et al. J. Child Neurol., 2015 | Duchenne Muscular Dystrophy (DMD) | To conduct a preliminary exploration into the use of accelerometry as a complementary functional outcome measure in DMD and to describe the relationship between StepWatch activity measures and standard of functional assessments | 16 | 9.0 ± 2.1 | 13 | 9.0 ± 2.4 |  |  | Accelerometer (StepWatch, Orthocare Innovations, Washington) | 1 on the ankle | Number of step, inactive (zero step rate), low activity (1-15 steps/min), medium activity (16-30 steps/min), and high activity (more than 30 steps/min). | Participants completed a 6-minute walk test and wore the sensor for 5 consecutive days. | Both the 6-minute walk test and StepWatch accelerometry identified a decreased capacity for ambulation in boys with Duchenne compared to healthy controls. There were strong, significant correlations between 6-minute walk distance and all StepWatch parameters in affected boys only (r = 0.701-0.804). |
| Fowler, et al. Muscle Nerve, 2018 | Duchenne Muscular Dystrophy (DMD) | To examine longitudinal activity in boys with DMD | 42 | 7.9 ± 2.9 |  |  |  |  | StepWatch Activity Monitor, Modus Health LLC, Washington, DC | 1 above the ankle | Strides/day and percentages of high, moderate and low frequency strides | Data were collected at 6-month intervals over a 5-year period with continuous enrollment. Data were excluded from the natural history analysis if a participant was enrolled in an experimental drug treatment trial during the data collection period. Parents and children were provided with a calendar to denote time on/off, school attendance, typical versus atypical day, and reasons for activity variation or removal of the device (e.g., illness, weather, bathing, swimming). Data were collected in the boys’ communities, including home and school. Participants were instructed to wear their monitor 3–5 weekdays and 2 weekend days following each laboratory evaluation. Additionally, data were compared with 10-meter walk/run (10mWR) speed. | There were significant declines in average strides/day and percent strides at moderate, high and pediatric high rates as a function of age (P < 0.05). Significant correlations for 10mWR versus high and low stride rates were found at baseline (P < 0.05). Step activity outcomes were sensitive to change over 1 year, but the direction and parameter differed by age group (younger vs. older). Changes in strides/day and percentages of high frequency and low frequency strides correlated significantly with changes in 10mWR speed (P < 0.05). |
| Lott, et al. Muscle Nerve, 2021 | Duchenne Muscular Dystrophy (DMD) | To explore walking activity in a large cohort of boys with DMD | 70 | 8.7 ± 2.0 | 10 | 9.2 ± 1.3 |  |  | Accelerometer (GT3X Actigraph, Actigraph Corporation, Pensacola, Florida) | 1 on the waist | Daily step count | Participants were asked to wear the device for all waking hours (except for bathing and swimming) for 7 consecutive days upon returning home. Ambulatory status was determined 2 years later. | Two to 5 days of activity monitoring predicted weekly step activity (adjusted R² = 0.80-0.95). Age comparisons revealed significant declines for step activity with increasing age, and relationships were found between step activity with both function and strength (P < 0.01). Our regression model predicted 36.5% of the variance in step activity. Those who were still ambulatory after 2 years demonstrated baseline step activity nearly double that of those who were no longer walking 2 years later (P < .01). |
| An, et al. Chaos, 2020 | Duchenne Muscular Dystrophy (DMD) | To evaluate the coordination of children with DMD during a walking task | 100 |  | 100 |  |  |  | Accelerometers (developed by APDM Mobility LabTM, Portland, OR, USA) | 5 on each forearm, each shank and core region | Relative coupling coefficient (RCC), Core-Limb Coupling Coefficient (CLCC) and Homolateral-Limb Coupling Coefficient (HLCC) | Participants were asked to walk 7 m at their comfortable speed, turn 180°, then walk back to the starting position, and repeat three times to calculate the Intraclass Correlation Coefficient (ICC). The road at the experimental site was flat, and there was no external interference during the experiment. | T-test results show that, for all age groups, children of the same age with DMD and controls show significant differences in RCC (p < 0.001). RCC comprehensively reflects that the coordination ability of DMD patients under walking tasks gradually decreases with age, which is consistent with clinical experience. In addition, with the growth of age, the CLCC of children with DMD gradually decreases, while HLCC presents an upward trend. However, CLCC and HLCC of controls showed no obvious trend, and the standard deviation was relatively large. |
| Jeannet, et al.  Eur. J. Paediatr. Neurol., 2011 | Duchenne Muscular Dystrophy (DMD) | To assess the feasibility and accuracy of physical activity monitoring of DMD patients using a non-commercialized inertial device | 5 |  |  |  |  |  | Tri-axial accelerometer and gyrscope (ASUR-Autono- mous Sensing Unit Recorder) | 1 on the patient’s tee-shirt, on the chest | Time spend sitting, standing, lying, walking, the number of steps taken, the cadence, the number of walking episodes, their duration and how these were distributed over the day | Participants underwent physical activity monitoring during two days in a home environment. Each patient was monitored for two consecutive days on two separate occasions: at baseline before prednisolone treatment, and at follow up one month after the treatment was started. | Parameters possibly reflecting endurance, such as the duration of the walking episodes or the succession of two or three walking episodes lasting more than 30 seconds were the most improved after prednisolone treatment. |
| Capelini, et al.  Neuropsychiatr. Dis. Treat., 2017 | Duchenne Muscular Dystrophy (DMD) | To verify whether individuals with DMD improve their motor performance when undertaking a visual motor task using a smartphone game | 50 | Group 1.  17.1 ± 5.3  Group 2.   17.2 ± 4.7 | 50 | Group 1.  17.1 ± 4.3  Group 2.  17.4 ± 4.4 |  |  | Smartphone game |  | Time to finish each maze | Participants moved a virtual ball around a virtual maze and the time in seconds was measured after every attempt in order to analyze improvement of performance after the practice trials. Motor performance was measured in phases of acquisition, short-term retention, and transfer. | Use of the smartphone maze game promoted improvement in performance during acquisition in both groups, which remained in the retention phase. At the transfer phases, with alternative maze tasks, the performance in DMD group was similar to the performance of control group, with the exception of the transfer to the contralateral hand (nondominant). However, the group with DMD demonstrated longer movement time at all stages of learning, compared with the control group. |
| Kimura, et al.  Pediatr. Int., 2014 | Duchenne Muscular Dystrophy (DMD) | To evaluate the utility of a wrist actigraph for estimating muscle strength in DMD | 22 | 13.8 ± 2.6 |  |  |  |  | Piezoelectric sensor( Actigraph, Motionlogger Watch; Ambulatory Monitoring, Ardsley, NY, USA) | 1 on the wirst | Zero crossing mode (ZCM), which indicates the frequency of movement and proportional integration mode (PIM), which indicates activity level or vigor of motion. | Participants wore a wrist actigraph to monitor activities of daily living, and underwent a test of knee extension strength and the 6 min walk test. These measures were made at baseline and at 1 year later. | The ZCM and PIM scores of ambulatory patients were higher than those of non-ambulatory patients (P < 0.001). The correlation coefficient between ZCM score and 6 min walk distance, ZCM score and knee extension strength, PIM score and 6 minute walk distance, and PIM score and knee extension strength was -0.44, 0.25, 0.58, and 0.63, respectively. This indicates that the PIM score had a moderate-good association with 6 min walk distance and knee extension strength. |
| van der Geest, et al.  Muscle Nerve, 2020 | Duchenne Muscular Dystrophy (DMD) | To evaluate the validity of home measurements of UE accelerometry | 16 | 12.4 ± 3.2 |  |  |  |  | Accelerometer (MOX Accelerometry; Maastricht Instruments BV, Maastricht, the Netherlands) | 3 on the upper arm, the lower arm, and on the wheelchair or the trousers | Activity counts (intensity), level of elevation and transfer of arm elevation | Patients were monitored for 1 to 3 days with accelerometers. Patients completed a physical activity diary (a diagram on paper) for 2 to 3 consecutive days. | The mean intensity of activity and the mean frequency of transfers of arm elevation from low to middle were approximately twofold higher in patients with a Brooke scale score of 1 or 2 than in patients with a Brooke scale score of 3 or 4. Correlations with the Performance of Upper Limb scale score were high for intensity and for the total frequency of arm elevations per hour. |
| Killian et al.  Neuromuscular Disord., 2020 | Duchenne Muscular Dystrophy (DMD) | To assess the correlation and longitudinal change of two measures: quantitative muscle testing (QMT) and accelerometry | 48 | 13.0 ± 4.3 |  |  |  |  | Triaxial accelerometers (GT3X+, ActiGraph, Pensacola, FL) | 2 on the wrist and ankle | Total vector magnitudes and awake vector magnitude | Patients with DMD were prospectively enrolled and underwent QMT and accelerometers for seven days at baseline, 1-, and 2-years. QMT measures were indexed to age. | QMT and accelerometry measures had a moderate or strong correlation, particularly indexed arm QMT with total wrist vector magnitude (rho=0.85, p<0.001), total indexed QMT with total wrist vector magnitude (rho=0.8, p<0.001) and indexed leg QMT with total ankle vector magnitude (rho=0.69, p<0.001). QMT and accelerometry measures declined significantly over time. Accelerometry correlates with QMT and indexed QMT in boys with DMD. A combination of QMT and accelerometry may provide a complementary assessment of skeletal muscle function in non-ambulatory boys with DMD. |
| Siegel, et al.  Muscle Nerve, 2020 | Duchenne Muscular Dystrophy (DMD) | To describe sleep impairment and its relationship to quality of life (QOL) and to evaluate associations between rest-activity, sleep quality, and 6-minute walk test (6MWT) in DMD | 54 (23 wore actigraphy) | 10.7 ± 3.7 |  |  |  |  | Accelerometer (Actiwatch 2,AW2; Philips Respironics, Bend, Oregon) | 1 on non-dominant wrist | total sleep time, wake after sleep onset, sleep efficiency, interdaily stability, intradaily variability, most active 10 hours and least active 5 hours | Participants wore countinuously for up to 10 days. | Pathologic sleep was reported in 11 (20%) participants and correlated with lower QOL but not with ambulatory status. In ambulatory participants who completed actigraphy, rest-activity rhythm fragmentation was associated with subjective sleep impairment. Habitual daytime activity level was associated with 6MWT performance. |
| Arteaga, et al.  J. Neuromuscular Dis., 2020 | Duchenne Muscular Dystrophy (DMD) | To assess the total amount and patterns of physical activity in patients with DMD using accelerometers | 49 | 13.6 ± 4.0 | 15 | 14.0 ± 2.3 |  |  | Accelerometer (Actigraph GT3X accelerometer, Actigraph, Pensacola FL, USA) | 2 on their dominant the wrist and the ankle | Sedentary, low- intensity, and moderate-to-vigorous physical activity or MVPA | Patricipants wore the device for 7 days and 24 hours per day. | Among 49 patients with DMD, 44 and 33 had valid recordings from the wrist and ankle accelerometers, respectively, and 11 of 15 healthy controls had valid recordings. Physical activity across intensity categories differed between study groups (p < 0.001). Patients with DMD spent on average 98.8% of their daytime in the sedentary and low-intensity categories. Compared to non-ambulatory, ambulatory patients spent more time in sedentary-3 and low-intensity-2 subcategories (p < 0.001). Amount of activity was lower in all patients than controls (p < 0.05) and in non-ambulatory than ambulatory patients and controls (p < 0.001), but similar between ambulatory patients and controls. Activity measures in patients were significantly affected by age and ambulation status (p < 0.05) but not corticosteroid use. |
| Davidson, et al.  Clin. Nutr., 2021 | Duchenne Muscular Dystrophy (DMD) | To compare the effect of a Standard nutritional supplement with an enhanced nutritional supplement combining three nutriceuticals on functional outcomes in ambulatory boys with DMD | 36 | Group 1.  8.7 ± 2  Group 2. 9.4 ± 2.6 |  |  |  |  | StepWatchTM accelerometer (Orthocare Innovations, WA USA) | 1 on the ankle | Steps and inactive minutes par day | A 50-week double blinded, randomized, controlled crossover trial was conducted to evaluate the effect of a multicomponent nutritional supplement on functional outcomes. Primary outcome measures were 6-min walk distance (6MWD) and community ambulation (StepWatch™ Activity Monitoring). Secondary outcome measures included body composition and quality of life. | Twenty-seven boys completed the intervention. Traditional crossover analysis demonstrated the enhanced supplement compared to the Standard supplement was associated with a difference of +12 metres in 6MWD, + 0.5 inactive minutes per day and -95 steps per day. A mixed effect model indicated a potentially clinically important effect of the enhanced supplement on the 6MWD of +31 metres. Mean serum 25 hydroxy-vitamin D levels at week 50 was 94 nmol/L. There was no observable effect of either supplement regime on body composition or quality of life. |
| Ferrer-Mallol, et al. Front. Pharmacol., 2022 | Duchenne Muscular Dystrophy (DMD) | To identify new digital biomarkers that could help to measure the efficacity of new treatment and that could provide robust real-world evidence | 11 | 13 | 1 |  |  |  | App (Atom5TM digital platform and video capture capabilities) |  | 1/time, step length, vertical heel-toe distance 2/, 3/ Raised arm area, raised arm symmetry, time to raise arm, Root-mean-square-error (RMSE) 4/ Time, leg alignement, foot-to-foot distance and foot alignment | Patients, parents and carers of DMD patients met and determined the physical capabilities of patients and the types of movement that patients were able to complete and record using the mobile app that were representative of the transition stage. The motor function tasks selected were: 1) walking, 2) hands-to-head while sitting, 3) hands-to-head while standing and, 4) sit-to-stand then hands-to-head while standing. These task were performed at home by participants.  A video analysis was performed using OpenPose, an open- source software that maps 25 points on the body. | A total of 62 videos were submitted by the eight participants, and 52 of these were used in the analysis. 83.4% of participants said that the video tasks and the instructions for the tasks were easy to understand. 83.4% of participants said their performance in the tasks were representative of their physical abilities whilst the remaining 16.6% said they were somewhat representative. |
| Jacques, et al.  Disabil. Rehabil., 2022 | Duchenne Muscular Dystrophy (DMD) | To quantify changes, from a one year follow up, in body composition, muscle strength, muscle size and physical activity levels in adults with DMD and Becker Muscular Dystrophy (BMD); and to identify the impact of changes in physical activity on body composition and muscle strength | 15 |  |  |  | 12 patients with BMD |  | Tri-axial accelerometer (GENEActiv, Kimbolton, Cambs, United Kingdom) | 1 on the wrist | Daily average minutes being physically active or average daily percentage of waking hours being sedentary (sedentary behaviour). | Participants were assessed at baseline and 12-months for body composition (Body fat and lean body mass (LBM)), Isometric maximal voluntary contraction (Knee-Extension (KEMVC) and Plantar-Flexion (PFMVC) and physical activity. | 12-Month change in strength was found as -19% (PFMVC) and -14% (KEMVC) in DMD. 12-Month change in strength in BMD, although non-significant, was explained by physical activity (R² = 0.532-0.585). Changes in LBM (DMD) and body fat (BMD) were both masked by non-significant changes in body mass. |
| van Eijk, et al. J. Neurol., 2019 | Amyotrophic lateral sclerosis (ALS) | To determine the value of remote, accelerometer-based monitoring of physical activity in patients with ALS | 42 (11) | 60 ± 12 |  |  |  |  | Tri-axial accelerometer (ActiGraph GT9X Link, ActiGraph LLC, Pensacola, FL) | 1 on the right hip | %active (the proportion of the vector magnitude counts that exceeded the 100 counts per minute threshold), metabolic Equivalent of Task score, daily VM (average daily vector magnitude count and its variation), daily A1 (variation in vertical axis) | Patients wore the sensor during waking hours for 7 days every 2–3 months and provided information regarding their daily functioning (ALSFRS-R). | A total of 42 patients participated; the total valid monitoring period was 9288 h with a 93.0% adherence rate. At baseline, patients were active 27.9% (range 11.6–52.4%) of their time; this declined by 0.64% (95% 0.43–0.86, p < 0.001) per month. Accelerometer-based endpoints were strongly associated with the ALSFRS-R (p < 0.001), but showed less variability over time than the ALSFRS-R (coefficient of variation 0.64–0.81 vs. 1.06, respectively). Accelerometer-based endpoints could reduce sample size by 30.3% for 12-month trials and 44.6% for 18-month trials; for trials lasting less than 9 months, the ALSFRS-R resulted in smaller sample sizes. |
| Garcia-Gencedo, et al. JMIR Mhealth Uhealth, 2019 | Amyotrophic lateral sclerosis (ALS) | To investigate the feasibility of a novel digital platform for remote data collection of multiple symptoms | 25 (4) | 53.1 ± 9.9 |  |  |  |  | Tri-axial accelerometer (Mega Faros) and 2-lead ECG sensor (Mega Electronics Ltd., Finland) | on the torso | Average daytime active, percentage of daytime active, total daytime activity score, total 24-hour activity score, maximum daytime activity score, mean maximum daytime activity score, number of active periods per hour, duration of active periods >1 minute | Participants attended a clinical site visit every 3 months to perform activity reference tasks while wearing a sensor, to conduct digital speech tests and for conventional ALS monitoring. In addition, patients wore the sensor in their daily life for approximately 3 days every month for the duration of the study. | The amount and quality of digital speech data captured at the clinical sites were as intended, and there were no significant issues. All the home monitoring sensor data available were propagated through the system and were received as expected. However, the amount and quality of physical activity home monitoring data were lower than anticipated. A total of 3 or more days (or partial days) of data were recorded for 65% of protocol time points, with no data collected for 24% of time points. At baseline, 24 of 25 patients provided data, reduced to 13 of 18 patients at Week 48. Lower-than-expected quality HRV data were obtained, likely because of poor contact between the sensor and the skin. In total, 6 of 25 patients had mild or moderate adverse events (AEs) in the skin and subcutaneous tissue disorders category because of skin irritation caused by the electrode patch. There were no reports of serious AEs or deaths. Most patients found the sensor comfortable, with no or minimal impact on daily activities. |
| Kelly, et al. Amyotroph. Lateral Scler. Frontotemporal Degener., 2020 | Amyotrophic lateral sclerosis (ALS) | To explore novel, real-world biotelemetry disease progression markers in patients with amyotrophic lateral sclerosis (ALS) and to compare with clinical gold-standard measures | 25 (4) | 53.1 ± 9.93 |  |  |  |  | Tri-axial accelerometer (Mega Faros) and 2-lead ECG sensor (Mega Electronics Ltd., Finland) | on the torso | Average daytime active, percentage of daytime active, total daytime activity score, total 24-hour activity score, maximum daytime activity score, mean maximum daytime activity score, number of active periods per hour and duration of active periods >1 minute | Participants wore biotelemetry sensors for approximativly 3 days per month at home, measuring physical activity, heart rate variability (HRV), and speech over 48 weeks. These measures were assessed longitudinally in relation to ALS Functional Rating Scale-Revised (ALSFRS-R) score and forced vital capacity (FVC); assessed by telephone [monthly] and clinic visits [every 12 weeks]) | Four endpoints showed moderate or strong between-patient correlations with ALSFRS-R total and gross motor domain scores : average daytime active; percentage of daytime active; total daytime activity score; total 24-hour activity score. Moderate correlations were observed between speech endpoints and ALSFRS-R bulbar domain scores; HRV data quality was insufficient for reliable assessment. The sensor was generally well tolerated; 6/25 patients reported mostly mild or moderate intensity skin and subcutaneous tissue disorder adverse events. |
| Liao, et al.  Front. Aging Neuroscience, 2022 | Amyotrophic lateral sclerosis (ALS) | To estimate the causal association between physical activity phenotypes and neurodegenerative diseases. |  |  |  |  |  |  | Accelerometer (Activity-AX3) | 1 on the wrist | Overall acceleration average (OAA) and fraction of accelerations > 425 milligravities (FAA) | Genetic variants robustly associated with PA phenotypes, used as instrumental variables, were extracted from public genome-wide association study (GWAS) summary statistics. GWAS information was also obtained from the most recent large population study of individuals with European ancestry. Multiple MR methods, pleiotropy tests and sensitivity analyses were performed to obtain a robust and valid estimation. Physical activity was assessed through questionnaires and accelerometry. | This study showed a positive association between moderate-to-vigorous physical activities and ALS based on the inverse variance weighted MR analysis method (p = 0.013). The pleiotropy test and sensitivity analysis confirmed the robustness and validity of these MR results. No causal effects of PA phenotypes were found on PD and AD. |
| Geronimo, et al. J. Med. Eng. Technol., 2021 | Amyotrophic lateral sclerosis (ALS) | To evaluate wearable-based methods for assessing gait to facilitating better monitoring of ambulatory health and ultimately lessen fall risk | 30 (11) |  |  |  |  |  | IMU sensors : accelerometer, gyroscope and magnetometer (MetaMotionR, mbientlabs, San Francisco, CA) | 2 on the back of the waist and over the dorsum of the left foot | Stride length, duration and walking speed | Participants were guided by a physical therapist on a short walk, during which inertial sensors recorded their movement. Two methods, utilising sensors at the waist or foot, were used independently to estimate gait parameters. | Decreased stride length, increased stride duration and decreased walking speed were associated with walking sub-score of the ALSFRS-R and the presence of a cane or walker. Overall, there was no group-wide mean walking speed differences between methods, though the waist method overestimated stride length and walking speed in those with more significant gait dysfunction compared to the foot method. Reconstruction of movement using the foot-based sensor resulted in route segments that were 94 ± 1% standard error of the mean (SEM) the length of a centre-to-centre hallway reference vector, with an angular error of 0.66 ± 0.28 SEM. |
| Rutkove, et al. Ann. Clin. Transl. Neurol., 2020 | Amyotrophic lateral sclerosis (ALS) | To determine the potential for improving amyotrophic lateral scle- rosis (ALS) clinical trials by having patients or caregivers perform frequent self-assessments at home | 61 (18) | 60.1 ± 9.9 |  |  |  |  | 1/ The Camry Handgrip Dynamometer (Camry Scale-USA, City Industry, CA) 2/ Electrical impedance myography (EIM). The Skulpt Scanner (aka Chisel, Myolex, Inc, Boston, MA) 2/ Spirometry with the AirSmart Spirometer (Nuvoair AB, Stockholm, Sweden).  3/ Speech analysis app and database (Aural Analytics 2020, Phoenix, Arizona, USA). 3/ The Mi Band® (Xiaomi Corp,H219 Beijing, China) | / | Steps, EIM, grip strenght | ALS patients were enrolled into a nonblinded, longitudinal 9-months study in which patients and caregivers obtained daily data using several different instruments, including a slow-vital capacity device, a hand grip dynamometer, an electrical impedance myography-based fitness device, an activity tracker, a speech app, and the ALS functional rating scale-revised. Questions as to acceptability were asked at two time points. Participants were instructed to obtain all outcome measures daily for 90 days, then twice weekly for an additional 180 days, except for the ALSFRS-R which participants collected weekly throughout the study. Participants with a minimum of 7 days data and were included in the analysis. | Daily measurements resulted in more accurate assessments of the slope of progression of the disease, resulting in smaller sample size estimates for a hypothetical clinical trial. For example, by performing daily slow-vital capacity measurements, calculated sample size was reduced to 182 subjects/study arm from 882/arm for monthly measurements. Similarly, performing the ALS functional rating scale weekly rather than monthly led to a calculated sample size of 73/arm as compared to 274/arm. Participants generally found the procedures acceptable and, for many, improved their sense of control of their disease. |
| Pancani, et al.  PLoS One, 2017 | Amyotrophic lateral sclerosis (ALS) | To quantitatively characterise head movements in ALS compared to aged matched controls | 13 (6) |  | 13 (6) |  |  |  | Tri-axial accelerometer, a tri-axial gyroscope and a tri-axial magnetometer (OPAL, APDM Inc., USA) | 2 on the forehead and sternum | Mean angular velocity (ωm), peak angular velocity (ωp), normalized jerk (NJ), and ratio of movement coupling (RMC) | Participants performed a series of controlled head movements while wearing two inertial sensors. Each participant was asked to sit on a chair and perform a series of active head movements: flexion (F), extension (E), axial rotation (AR, toward their left and right side) and lateral flex- ion (LF, toward their left and right side), starting from their own neutral position (NP) and looking ahead. | Results confirmed a general limitation in the ability of the ALS patients to perform and control head movements. High inter-patient variability was observed due to a wide range of functional impairment levels. The ability to extend the head backward and flex it laterally were the most compromised, with significantly lower angular velocity (P < 0.05, Cohen’s d > 0.8), reduced smoothness and greater presence of coupled movements with respect to the controls. A significant reduction of angular velocity (P < 0.05, Cohen’s d > 0.8) in extension, axial rotation and lateral flexion was observed when patients were asked to perform the movements as fast as possible. |
| Pancani, et al. Clin. Biomech. (Bristol, Avon), 2018 | Amyotrophic lateral sclerosis (ALS) | To characterise the ability of this orthosis to provide head support and facilitate the control of head movements in people living with Amyotrophic Lateral Sclerosis | 13 (6) |  |  |  |  |  | Tri-axial accelerometer, a tri-axial gyroscope and a tri-axial mag-netometer (OPAL, APDM Inc., USA) | 2 on the forehead and sternum | Mean angular velocity (ωm), peak angular velocity (ωp), normalized jerk (NJ), and ratio of movement coupling (RMC) | Participants were asked to perform a series of head movements with and without wearing the collar. Two parameters (mean angular velocity and ratio of movement coupling) were extracted from recorded angular velocities, to quantify changes in the execution of the movement between the two conditions. | Participants exhibited different levels of impairment in performing different movements. When wearing the collar self-selected movement velocity was preserved and significant improvement in the control of lateral flexion movement was observed (median ratio of movement coupling value reduced from 1.1 to 0.84, P = 0.013). A lower ratio of movement coupling was also observed in 4 out of 7 individuals that were fitted with anterior supports. |
| Vieira, et al.  NPJ. Digit. Med., 2022 | Amyotrophic lateral sclerosis (ALS) | To provide an objective measure for ALS disease severity. | 1/ 395 (134) 2/ 80 (28) 3/ 109 (43) | 1/ 58.7 ± 12 2/ 57.8 ± 12 3/ 59.3 ± 10 |  |  |  |  | Accelerometer (Actigraph GT3X devices). | 4 on each limbs | Total Body Vector Magnitude (TBVM), FFT 1 Hz, Uniform 1 Hz. | The accelerometer measurements, obtained from Actigraph GT3X devices (one for each limb), came from 5 limb-based exercises each about 45 s long with a short 15 s break in between. A full set of measurements is approximately 5 minutes. A machine learning (ML) based objective measure for ALS disease severity was developed based on voice samples and accelerometer measurements from a four-year longitudinal dataset. | 350 participants contributed 13,009 accelerometer samples, while simultaneously measuring ALSFRS-R scores. Using these data, we trained ML models to predict bulbar-related and limb-related ALSFRS-R scores. On the test set; the voice models achieved a multiclass AUC of 0.86 on speech ALSFRS-R prediction, whereas the accelerometer models achieved a median multiclass AUC of 0.73 on 6 limb-related functions. The correlations across functions observed in self-reported ALSFRS-R scores were preserved in ML-derived scores. We used these models and self-reported ALSFRS-R scores to evaluate the real-world effects of edaravone, a drug approved for use in ALS. In the cohort of 54 test participants who received edaravone as part of their usual care, the ML-derived scores were consistent with the self-reported ALSFRS-R scores. At the individual level, the continuous ML-derived score can capture gradual changes that are absent in the integer ALSFRS-R scores. |
| Agurto, et al.  Annu. Int. Conf. IEEE Eng. Med. Biol. Soc., 2019 | Amyotrophic lateral sclerosis (ALS) | To characterize ALS subjects as well as their progression using acoustic and fine motor features | 43 (13) | 60.2 ± 6.8 | 29 | 53.1 ± 8.8 |  |  | An app called “Help us Answer ALS”. The app integrates 7 different tasks that can be used to monitor motor control in the upper body, speech and cognition. |  | 1/ Error Metrics 2/ Velocity rate, estimated by the number of points recorded for the tracing. | Data was collected with the app. Each task is performed once per week covering each of the days of the week . In addition, clinical variables such as speech, writing and total ALSFRS-R scores are also acquired along with forced and slow vital capacity. | Results show that both types of features are useful to infer clinical variables especially for males (R2=0.79 for ALSFRS-R total score), but their initial values are not helpful to predict speech and motor decline. However, we found that longitudinal progression for bulbar and spinal ALS onset are different and they can be identified with high accuracy by the extracted features. |
| Baxi, et al. Nat. Neurosci., 2022 | Amyotrophic lateral sclerosis (ALS) | To provide large clinical and biological datasets in an open source-like application that affords researchers the proper tools to identify biological subgroups and an extensive collection of IPS cell lines with which to test ALS therapies and hypotheses about ALS pathogenesis | 861 (320) | 59.3 ± 11.1 | 108 (71) | 55.0 ± 14.1 | 66 (25) non-ALS motor neuron disease | 61.9 ± 12.0 | App |  | Performance values | Answer ALS is a biological and clinical resource of patient-derived, induced pluripotent stem (iPS) cell lines, multi-omic data derived from iPS neurons and longitudinal clinical and smartphone data from over 1,000 patients with ALS. A unique smartphone-based system was employed to collect deep clinical data, including fine motor activity, speech, breathing and linguistics/cognition. | Features from the finger tracing showed modest individual correla- tions with the ALSFRS-R total score. The combination of features from all tasks (vounting, picture, reading, tracing )  correlated very highly with the ALSFRS-R total score (Pearson’s R = 0.89). |
| Londral, et al. Clin. Neurophysiol., 2016 | Amyotrophic lateral sclerosis (ALS) | To explore movement activity in the use of keyboards and identify markers for upper limb (UL) dysfunction in ALS | 19 (16) |  |  |  | 6 patients with neuromucular disorders |  | Tri-axial accelerometer. | 1 on the index finger | Mean keyhold time, time to press, acceleration to press, acceleration to release | Participants performed the same 10-word typing task (2–6 assessments for 2 to 20 months) wearing the accelerometer. | During disease progression, mean time in holding down a key increased and was longer than in control subjects. Acceleration at key press and key release decreased with progression of UL dysfunction. Delay between tapping and pressing down each key increased with upper limb dysfunction. |
| Vogelnik, et al. J. Neurol., 2022 | Amyotrophic lateral sclerosis (ALS) | To describe phenomenology, prevalence and pathophysiology of involuntary movements in motor neuron disease | 14 |  |  |  |  |  | 1/ Triaxial accelerometer (Biometrics Ltd; sensitivity ± 50 mV/G) 2/ surface electromyography (EMG) | 1/ 2 both third metacarpal bone 2/ 2 forearm flexors and forearm extensors | Frequency | Accelerometry with electromyography was recorded in a subset of patients. Recording was performed in 3 situations : with hands resting in the lap, half pronated, while the patient extend and abduct the thumb against gravity with hands supported in the lap half pronated (postural condition) and in postural condition with 50 g mass attached to the thumb (weight loading). | Involuntary movements were observed in 68.9% of patients and could be separated into rest minipolymyoclonus, thumb tremor, pseudodystonic thumb posture, action minipolymyoclonus, and action tremor. One-third of patients reported negative impact of involuntary movements on hand use. Logistic regression showed that rest minipolymyoclonus and thumb tremor were more likely to occur in patients with more prominent distal muscle weakness and less spasticity. Similarly, action involuntary movements were more likely to appear in weaker patients. Patients with brisk tendon reflexes were more likely to display action tremor than action minipolymyoclonus. Action tremor was characterized by accelerometer and corresponding electromyography peak frequency, which decreased with mass loading, suggesting a mechanical-reflex tremor. |
| Crook-Rumsey, et al. Muscle Nerve, 2022 | Amyotrophic lateral sclerosis (ALS) | To establish an optimal recording duration to enable longitudinal remote monitoring of fasciculation at home | 20 (2) | 63.6 ± 9 |  |  | 5 (0) patients with benign fasciculation syndrome | 41.6 ± 6.9 | High-density surface electromyography (64 electrodes) | On the biceps brachii and gastrocnemii | Fasciculation frequency, median fasciculation amplitude, IQR fasciculation amplitude and total fasciculation number | Participants underwent serial 30 min HDSEMG recordings from biceps brachii and gastrocnemii. SPiQE was independently applied to abbreviated epochs within each 30-min recording (0–5, 0–10, 0–15, 0–20, and 0–25 min), outputting fasciculation frequency, amplitude median and amplitude interquartile range. Bland–Altman plots and intraclass correlation coefficients (ICC) were used to assess agreement with the validated 30-min recording. | In total, 506 full recordings were included. The 5 min recordings demonstrated diverse and relatively poor agreement with the 30 min baselines across all parameters, muscles and patient groups (ICC = 0.32–0.86). The 15-min recordings provided more acceptable and stable agreement (ICC = 0.78–0.98), which did not substantially improve in longer recordings. |
| Meyer, et al. Amyotroph. Lateral Scler. Frontotemporal Degener., 2022 | Amyotrophic lateral sclerosis (ALS) | To using digital data to capture was investigated for its feasibility as an add-on to ALSFRS-R assessments during multidisciplinary clinic visits. | 893 (743) | 60.1 ± 12.1 |  |  |  |  | ALS-App |  |  | Participants performed remote assessments of the ALSFRS-R. In addition to the assessment of ALSFRS-R during clinic visits, patients were offered a digital self-assessment of the ALSFRS-R – either on a computer or on a mobile application (“ALS-App”) | An estimated multicenter cohort of 4,670 ALS patients received care at participating ALS centers. Of these patients, 971 remotely submitted the ALSFRS-R, representing 21% of the multicenter cohort. Of those who opted for remote assessment, 53.7% (n 1⁄4 521) completed a minimum of 4 ALSFRS-R per year with a mean number of 10.9 assessments per year. Different assessment frequencies were found for patients using a computer (7.9 per year, n1⁄4857) and mobile app (14.6 per year, n1⁄4234). Patients doing remote assessments were more likely to be male and less functionally impaired but many patients with severe disability managed to complete it themselves or with a caregiver. |
| Peterson, et al.  Gait Posture, 2021 | CMT | To characterize performance fatigability during gait, and assessing its relation to life satisfaction | 31 (24) | 56.83 ± 10.78 |  |  |  |  | inertial sensors including accelerometers, gyroscopes, and magnetometers (OPALs, APDM, Portland Oregon) | 4 on each feet, the pelvis, and the trunk (sternum) | Step time, cadence, stride length, trunk range of motion (ROM) per stride, walking velocity, step time asymmetry, cadence Variability, stride length variability, step time variability and trunk ROM variability | Participants completed a 6-minute, fast-as-possible walk while gait outcomes were captured via inertial sensors. Perceived fatigue and general life satisfaction were assessed via questionnaire. | Five mean gait outcomes measured, four showed statistically significant changes over the 6-minute fast-as-possible walk: velocity (reduced; p=0.008); cadence (reduced; p<0.001), step time (increased; p < 0.001), and trunk ROM (increased; p = 0.032). Of the four variability and one asymmetry outcomes, only stride length variability changed during the walking task (p = 0.015), decreasing from bins 1–2, and remaining stable for bins 2–6. Changes in velocity, cadence, step time were related to general life satisfaction (0.038 < ps<0.04), but not perceived fatigue (ps>0.343). |
| Menotti, et al. Brain Behav., 2014 | CMT | To quantify daily living activities in CMT1A patients by means of inertial sensors | 8 (5) | 35.9 ± 9.9 | 8 (5) | 35.1 ± 11.2 |  |  | Inertial sensor (IDEEA, Intelligent Device for Energy Expenditure and Activity; MinisunLLC, Fresno, CA) | 5 on the chest, on each thigh, and each sole | Time, count, speed, and power of resting, walking, running, and jumping | Time and count (amount), and velocity and power (intensity) of 24 h daily living activities were measured in participants by means of a wearable inertial sensor device. | There were no differences between patients and controls in the 24-h distance covered and count of steps. However, count of step climbing and sit to stand were lower in patients than in controls as well as mean daily step-climbing and walking velocities. In CMT1A patients there was a positive correlation between strength of the knee extensor muscles and both count of steps climbed (R = 0.80) and sit to stand (R = 0.79). |
| Ramdharry, et al. Disabil. Rehabil., 2017 | CMT | To compare physical activity, patterns of sedentary behavior and overall energy expenditure of people with CMT and healthy matched controls | 20 |  | 20 |  |  |  | Multi-sensor device that incorporates skin temperature alvanic skin response and bi-axial accelerometry (SenseWear Pro3, Bodymedia Inc, PA, USA) | 1 on the right arm | Calorie expenditure (kcal), energy expenditure, steps taken, time spent in sedentary, moderate and vigorous activities . | Patient were asked to wear an activity monitor for 7 days. | Results showed a decrease in daily steps taken in the CMT group, but somewhat paradoxically, they demonstrate shorter bouts of sedentary activity and more frequent transitions from sedentary to active behaviors. No differences were seen in energy expenditure or time spent in sedentary, moderate or vigorous activity. |
| Pazzaglia, et al. Neuromuscular Disord., 2019 | CMT | To determine if these outcome measures are sensitive to change over a 12-month period | 149 (76) | 42.5 ± 12.5 |  |  |  |  | Accelerometer (StepWatchTM Activity Monitor (SAM), Cyma Inc. Seattle, USA) | 1 on the ankle | 1/ Duration, percentage of at none, low, medium, high activity.  2/ number and average of step at low, medium, high activity.  3/ Sustained activity measures: max 1, max 5, max 20, max 30, max 60 [step/min]  4/ Activity index [step/min] | The recording time was 5 days and patients were instructed remove it only for sleeping at night and showering. The 6MWT was performed according to the guidelines provided by the American Thoracic Society. All evaluation were repeated after 12 months. | Statistical analysis showed a worsening of the CMT-Neuropathy Score (p < 0.05), strength of distal muscles measured by myometry (p < 0.05) and StepWatchTM Activity Monitor outputs (p < 0.05). The 10 meter walking test (p > 0.05), muscular strength as detected by clinical evaluation (p > 0.05), 6-minute walk test (p > 0.05), pain (p > 0.05) and quality of life (p > 0.05) showed no change. In the current study, patients showed clinical worsening over 12 months, confirmed by a reduction of activity as detected by StepWatchTM Activity Monitor. The 6-minute walk test failed to detect change |
| Padua, et al. Eur. J. Neurol., 2016 | CMT | To evaluate the reliability and validity of the 6MWT and SAM as clinical tools reflecting patients’ activities of daily living in CMT1A, CMT1B and CMTX | 168 (87) | 44.4 ± 13.7 |  |  |  |  | Accelerometer (StepWatchTM Activity Monitor (SAM), Cyma Inc. Seattle, USA) | 1 on the ankle | 1/ Duration, percentage of at none, low, medium, high activity.  2/ number and average of step at low, medium, high activity.  3/ Sustained activity measures: max 1, max 5, max 20, max 30, max 60 [step/min]  4/ Activity index [step/min] | Subjects were instructed to wear the monitor for 5 days removing it only for sleeping and showering. Participants were also assessed with the CMT Neuropathy Score, the 10-m timed walking, hand-held myometry and the 36 item Short Form questionnaire. | Statistical analysis showed that the 6MWT was highly related with all previously used OMs. Some, but not all, SAM parameters were related to commonly used OMs but may provide more information about quality of life. |
| Saifee, et al. Clin. Neurophysiol., 2015 | CMT | To clarify the potential role of the cerebellum in CMT tremor | 16 |  |  |  |  |  | Triaxial accelerometer (Biometrics Ltd; sensitivity ±50 mV/G) | 2 on each index finger.  Surface EMG from biceps brachii (BB), forearm flexors (FF), forearm extensors (FE) and abductor pollicis brevis (APB) bilaterally | Peak tremor frequency, total power of the spectra on each accelerometer axis and the square root of the sum of the squares of each axis (derived for each measure of total power) | 1/ Assessed prevalence of tremor by questionnaire 2-3/ Participants underwent a clinical assessment, classical eye-blink conditioning, electro-oculography, visuomotor adaptation test, tremor recording with surface EMG and accelerometry, and retrospective correlation with nerve conduction studies to investigate the possible mechanisms of tremor generation. Recordings were performed with arms outstretched (postural condition) and in postural condition with 500 g mass attached to the hand (weight loading). | The prevalence study revealed tremor in 21% of patients and in 42% of those it caused impairmentof function. Tremor recordings revealed a mild-to-moderate amplitude tremor with a weight load-invariant 7.7 Hz frequency component. For tremulous subjects, there was no correlation between age and frequency of postural arm tremor. There was no difference in postural tremor frequency between males, 7.6 (0.61) Hz and females, 8.3 (2.4) Hz with tremor. There was no difference in EMG peak frequency between proximal and distal muscles. Six of ten tremulous patients had an EMG peak at the same frequency as the main accelerometer peak. Weight loading and alteration of posture had no effect on the postural tremor frequency in tremulous patients as a group. However, with weight loading, four patients demonstrated a secondary accelerometry peak with lower frequency. |
| Knak, et al. J. Neurol., 2020 | Myotonic Dystrophy (DM) | To assess physical activity and predictors of physical activity in individuals with myotonic dystrophy type 1 (DM1) | 67 (32) | 41 ± 10 | 39 (21) | 39 ± 11 |  |  | Accelerometer (wGT3X-BT, Timik Medical, Herlev, Denmark) | 1 on the hip | Time spent in light, moderate, vigourous, very vigourous activity | Physical activity was monitored by an accelerometer and assessed using the International Physical Activity Questionnaire. Patients wore the device over 7 consecutive days for 24 h, removing it only when showering or performing water activities. | The individuals with DM1 were on average − 187 min (p < 0.00001) objectively and − 48% (p = 0.001) subjectively less physically active per week compared to healthy controls. Education was the only predictor of physical activity in DM1 (p = 0.02). |
| Jimenez-Moreno, et al. Disabil. Rehabil., 2019 | Myotonic Dystrophy (DM) | To compare accelerometry data between a DM1 cohort and healthy controls, to explore the reliability of their use to detect ambulation at different speeds, and to assess which body location to place these devices when measuring ambulation in the DM1 population | 30 (10) | 48 | 14 (8) | 48 |  |  | Tri-axial accelerometer (GENEActiv, Activinsights Ltd., Cambridgeshire, United Kingdom) | 4 on each wrist and ankle | Acceleration in three planes | Participants wore while performing diiferent assessements : stand still for a minimum of 10s; 6min walking test (6MWT); 10 m walking test (10-mWT), where patients were requested to walk at their comfortable speed; 10 m walk/run test (10-mW/RT), where patients were requested to walk as fast as possible and if considered capable, running was allowed. | They found a high intra-accelerometer reliability (i.e. 0.97 to 0.99; p<0.001). For each test acceleration values differ significantly between each other and there was no inter-accelerometer reliability between wrist-worn devices and ankle-worn. They observed a significant difference between the myotonic dystrophy group and the healthy-controls detectable at each test. |
| Bachasson, et al. Neuromuscular Disord., 2016 | Myotonic Dystrophy (DM) | To evaluate gait using lower-trunk accelerometry and investigated relationships between gait abnormalities, postural instability, handgrip myotonia, and weakness in lower-limb and axial muscle groups commonly affected in myotonic dystrophy type 1 (DM1) | 22 (11) | 42 | 20 (11) | 44 |  |  | Triaxial accelerometer (Locometrix®, Centaure Metrix, Evry, France) | 1on the waist (L3–L4 inter-vertebral space) | Speed, stride frequency, stride length, stride length:height, root mean square of the accelerations in all three directions normalized to gait speed, interstride regularity AP (anteroposterior) and V (vertical), gait asymmetry AP and V, entropy of the acceleration signal AP, ML (mediolateral) and V, coefficient of variation of stride times, ratio of high-over low-frequency in the acceleration signal in all three direction. | Wearing the sensor, participants performed a 6-min walk test according to standard guidelines. Postural stability was measured via center of pressure displacement analysis using a force platform during eyes-closed normal stance. Handgrip myotonia was quantified using force-relaxation curve modeling. | Patients displayed lower walking speed, stride frequency, stride length, gait regularity, and gait symmetry. Strength of ankle plantar flexors, ankle dorsal flexors and neck flexors correlated with interstride regularity in the vertical direction. Knee extension strength correlated with gait symmetry in the anteroposterior direction. Center of pressure velocity was greater in patients and correlated with neck flexion and ankle plantar flexion weakness, and with interstride regularity in the vertical direction. No correlation was found between handgrip myotonia and any other variable studied. |
| Hamel, et al. Muscle Nerve, 2022 | Myotonic Dystrophy (DM) | To test the feasibility to use remote study visits to evaluate patients with myotonic dystrophy type 1 (DM1) | 23 (15) | 44 ± 16 |  |  |  |  | iPad 7 tablet (Apple) and stand (Manfrotto), spirometer (NuvoAir), grip dynamometer (Jamar Plus Digital; JLW Instruments), and 3-m tape measure. |  | Grip strength, forced vital capacity, peak cough flow, timed-up-and-go (TUG), and grip myotonia (hand opening time) | Toolkits containing a tablet computer, grip dynamometer, and spirometer were shipped to participants. The tablets were loaded with software for video-conferencing and questionnaires about functional impairment, patient experience with technology, and willingness to participate in future remote studies. The grip dynamometer is digital, and the result from each successive trial was displayed to the investigator. Grip myotonia was assessed by recording video hand opening time (VHOT). Mobility was assessed using the timed up and go (TUG). | All 23 subjects completed the remote visits. 95% of participants were able to complete all components of the remote study. All toolkit components were returned upon completion. Grip strength and TUG demonstrated moderate to strong correlations with self-reported inventories of upper and lower extremity impairment, respectively. A total of 91% of subjects expressed interest in participating in future remote visits. |
| Gidaro, et al. Muscle Nerve, 2022 | Facioscapulohumeral dystrophy (FSHD) | To report the data of exploratory digital outcomes extracted from wearable magneto-inertial sensors used in a non-controlled environment for ambulant patients with FSHD and LGMDR2. | 5 |  |  |  | 5 patients with LGMDR2 |  | Inertial sensors (ActiMyo®, Sysnav, France) | 2 on the wrist and ankle | Stride length (median and 95th centile), stride speed (median and 95th centile), distance walked per hour, number of stride per hour | A subset of participants of the open-label study ATYR1940-C-004 were asked to were an inertial device during the 3 months of ATYR1940 treatment and 1 months of follow-up. Activity and gait variables were calculated from the data recorded in 30-day sub-periods using the sensors. For each sub-period, activity and gait parameters were compared between FSHD and LGMDR2 patients. Sensitivity to change was assessed over the 4-months follow-up | Ten patients were ambulant and compliant for analysis. Gait parameters, but not activity variables, were significantly lower in LGMDR2 compared to FSHD patients at baseline. Longitudinal analyses showed a slight but significant decrease in stride speed at month 4 for all subjects. Activity variables such as total number of strides per day were highly variable from month to month in individual patients, and no visit effects were found for this variable. |
| Huisinga, et al Muscle Nerve, 2018 | Facioscapulohumeral dystrophy (FSHD) | To evaluate specific gait metrics obtained during an instrumented TUG (iTUG) in persons with FSHD to identify the test–retest reliability of those metrics and to examine the relationship between these metrics and FSHD disease severity | 17 (10) | 53.7 |  |  |  |  | Triaxis accelerometer and gyroscope (Mobility Lab, Opal sensors; APDM, Portland, OR) | 6 on the sternum, the lumbar spine, each ankle and wrist | Total duration, stride length, stride velocity, cadence, double support, RoM knee, RoM knee asymmetry, RoM trunk horizontal, RoM trunk sagittal, RoM arm, RoM arm asymmetry, Turn: duration. | Participants performed an instrumented timed up and go (iTUG) test using a commercially available system of wireless motion sensors. Patients returned within 2 weeks to determine test–retest reliability. Gait parameters in FSHD participants were compared with a normative database, FSHD clinical severity score, manual muscle testing, and patient-reported functional disability. | Gait parameters in FSHD participants were significantly (P < 0.05) altered compared with normative values, and reliability was excellent (intraclass correlation coefficient 0.84–0.99). Stride velocity and trunk sagittal range of motion had moderate to strong correlations to other FSHD disease measures. |
| Statland, et al. Muscle Nerve, 2019 | Facioscapulohumeral dystrophy (FSHD) | To examine changes in instrumented timed functional motor tasks variables | 10 (4) | 54 ± 8.2 |  |  |  |  | Accelerometer, gyroscope, and magnetometer (Mobility Lab, APDM, Portland, Oregon) | 6 on each wrist, the sternum, the lumbar area and each ankle. | Total duration, stride length, stride velocity, cadence,  double support, ROM knee,  ROM trunk sagittal, ROM arm,  turn duration, iTUG Z score | Participants performed an instrumented timed up and go (iTUG) trial at each visit, wearing the sensors. Participants stood up from a chair, walked 7 meters, turned around, walked 7 meters, turned, and sat down. | For an average of 20.6 months, the iTUG duration stayed constant, whereas stride length, stride velocity, and trunk sagittal range of motion changed, indicating poorer performance. Arm swing changed in a compensatory direction toward the normative mean. |
| Maleki, et al. JMIR Form. Res., 2022 | Facioscapulohumeral dystrophy (FSHD) | To investigate the feasibility of using smartphones and wearables to capture symptoms related to FSHD based on a continuous collection of multiple features, such as the number of steps, sleep, and app use. | 38 (23) | 45 ± 14 |  |  | 20 (11) | 33 ± 12 | 1/ Accelerometer, apps, GPS, Google Places Calls, Microphone (smartphone) 2/ Withings Steel HR smartwatch (Withings Health) | 1/ 1 2/ 1 on the wrist | 1/ Maximum magnitude of the acceleration, number of times an app is opened, amount of time app is open; total kilometers per day; average kilometers per trip, 95% maximum distance from home, number of unique places visited; time spent at each unique location 2/ Total step count; mean steps per minute; mean steps per hour; maximum steps per hour  These device also recorded parameters relative to heart rate, sleep pattern, voice and blood pressure | Participants were monitored using a smartphone monitoring app for 6 weeks. On the first and last day of the study period, clinicians assessed the participants’ FSHD clinical score and Timed Up-and-Go test time. Participants installed the app on their Android smartphones, were given a smartwatch, and were instructed to measure their weight and blood pressure on a weekly basis using a scale and blood pressure monitor. The user experience and perceived burden of the app on participants’ smartphones were assessed at 6 weeks using a questionnaire. With the data collected, we sought to identify the behavioral features that were most salient in distinguishing the 2 groups (FSHD and controls) and the optimal time window to perform the classification. | Overall, the app was well tolerated, but 67% of participants (39/58) noticed a difference in battery life. Using the 6 weeks of data, the classification between patients with FSHD and non-FSHD controls with 93% accuracy, 100% sensitivity, and 80% specificity. We found that the optimal time window for the classification is the first day of data collection and the first week of data collection, which yielded an accuracy, sensitivity, and specificity of 95.8%, 100%, and 94.4%, respectively. Features relating to smartphone acceleration, app use, location, physical activity, sleep, and call behavior were the most salient features for the classification. |
| O’Connor, et al. J. Neuromuscular Dis., 2019 | Myasthenia Gravis (MG) | To evaluate baseline patterns of physical and sedentary behavior in MG patients | 27 (13) | 62 ± 16 |  |  |  |  | Accelerometer (DynaPort Move-Monitor, McRoberts, The Hague, The Netherlands) | 1 on the lower back | The amount of time spent in moderate and vigorous intensity activities, physical activity level (PAL), number of steps/day and sedentary time | Participants wore the sensor for seven consecutive days. Correlations between physical activity assesment and disease severity were analyzed. The results were compared to general recommendations and published data of healthy individuals and to data of patients with the chronic disorders chronic obstructive pulmonary disease (COPD) and mitochondrial myopathy. | MG patients had sedentary behavior during 78 ± 7% of the day. There was neither a correlation between disease severity and number of steps/day (R = –0.15; p = 0.56) nor between disease severity and PAL (R = 0.33; p = 0.26). Nevertheless, the MG patients met the recommendations of daily deliberate exercise (181 ± 158 MET min/day). PAL was lower in MG patients (1.5 ± 0.138) than in healthy individuals (1.67 ± 0.145, p < 0.00001). |
| Birnbaum, et al. J. Neuromuscular Dis., 2021 | Myasthenia Gravis (MG) | To describe habitual PA patterns and explore relationships between PA metrics, clinical MG characteristics, and health-related QoL (HRQoL) | 33 (33) | 45.4 ± 10.6 | 66 (66) | 46.1 ± 12.0 |  |  | Triaxial accelerometer (Version 3.0 DynaPort Move- 126 monitor, McRoberts, The Netherlands) | 1 on the lumbar spine | Total volume PA, time  (minutes) spent in different PA intensities (light, moderate, Moderate-to vigorous-intensity) and time spent sedentary | Participants wore the device for seven days. MG-specific evaluations, the six-minute walk test and knee extension strength were assessed in individuals with MG. | Patients with MG perform less vigorous-intensity PA than control subjects (p = 0.001), spend more time sedentary (p = 0.02) and engage in less and shorter durations of moderate-vigorous-intensity PA (MVPA). For patients with MG, habitual PA correlated positively with 6 min walking distance (rho = 0.387, p = 0.029) and negatively with body mass index (rho = –0.407, p = 0.019). We did not find any association between PA or sedentary behaviour and; HRQoL, symptom severity nor lower limb strength. |
| Annoussamy, et al. Ann. Clin. Transl. Neurol., 2021 | Spinal muscular atrophy (SMA) | To characterize the natural history of spinal muscular atrophy (SMA) over 24 months using innovative measures such as wearable devices, and to provide evidence for the sensitivity of these measures to determine their suitability as endpoints in clinical trials | 81 |  |  |  |  |  | Tri-axial accelerometer and a tri-axial gyroscope and tri-axial magnetometer (Actimyo, Sysnav, Vernon, France) | 1 on the wheelchair and the wirst | The wrist angular velocity, the wrist acceleration, the wrist vertical acceleration against gravity, the power and the percentage of activity time | Patients with Type 2 and 3 SMA who were not receiving disease-modifying treatment were assessed over 24 months: motor function (Motor Function Measure [MFM]), upper limb strength (MyoGrip, MyoPinch), continuous remote upper limb activity (ActiMyo), quantitative magnetic resonance imaging (fat fraction [FFT2] mapping and contractile cross-sectional area [C-CSA]), pulmonary function (forced vital capacity [FVC], peak cough flow, maximum expiratory pressure, maximum inspiratory pressure, and sniff nasal inspiratory pressure), and survival of motor neuron (SMN) protein levels. | MFM32 scores declined significantly over 24 months, but not 12 months. Changes in upper limb activity could be detected over 6 months and continued to decrease significantly over 12 months, but not 24 months. Upper limb strength decreased significantly over 12 and 24 months. FVC declined significantly over 12 months, but not 24 months. FFT2 increased over 12 and 24 months, although not with statistical significance. A significant increase in C-CSA was observed at 12 but not 24 months. Blood SMN protein levels were stable over 12 and 24 months. MFM 32 and grip strength were found to have high correlation with wrist acceleration as measure by ActiMyo. Upper limb activity was shown to decrease significantly ovre 6 and 12 months as measures in patients with Type 2 SMA (sitters and non-sitters) and non-ambulant with Type 3. |
| Shrader, et al. Ann. Clin. Transl. Neurol., 2015 | Spinal and bulbar muscular atrophy (SBMA) | To determine the safety and efficacy of a home-based functional exercise program in SBMA | 54 enrolled and 50 completed the study : 1/ 24 in the functional exercise group 2/ 26 in the stretching control group | 1/ 53.8 ± 10.0  2/ 56.5 ± 8.1 |  |  |  |  | Actical accelerometer (Philips Respironics, Bend, OR) | 1 | Average count per day | Participants were randomly assigned to participate in 12 weeks of either functional. exercises (intervention) or a stretching program (control). The primary outcome measure was the Adult Myopathy Assessment Tool (AMAT) total score, and secondary measures included total activity by accelerometry, muscle strength, balance, timed up and go, sit- to-stand test, health-related quality of life, creatine kinase, and insulin-like growth factor-1. The accelerometer was worn during the first 10 days of the trial and during the last 10 days before the final evaluation. | Functional exercise was well tolerated but did not lead to significant group differences in the primary outcome measure or any of the secondary measures.To determine whether a subset of the subjects may have benefited, we divided them into high and low functioning based on baseline AMAT scores and performed a post hoc subgroup analysis. Low-functioning individuals receiving the intervention increased AMAT functional subscale scores compared to the control group. Individuals with higher AMAT baseline functional subscale scores who received the intervention became more active, showing an increase in total activity count per day by accelerometry when compared to the control group, accounting for age differences. |
| Gordon, et al. Digit. Biomark., 2019 | Huntington disease (HD) | To develop algorithms that could objectively quantify chorea by analyzing the data from the wearable sensors and to provide insight into the daily variability of HD motor symptoms outside the clinical setting | 17 (8) were screened and 15 were enrolled | 51 ± 12 |  |  |  |  | 1/ A smartphone application  2/ Triaxial accelerometer (smartwatch and smartphone) | 2/ 2 on the wrist of the chorea-dominant upper limb or on the nondominant limb, the chorea-dominant upper limb could not be determined (smartwatch) and on a location next to the first one (i.e., in the patient’s waist pocket or holstered in the waist area) (smartphone) | Chorea occurrence and severity | A subset of patients enrolled in the Open-PRIDE-HD study (treated with 45 mg pridopidine twice daily) were asked to participate in this device substudy. Patients were asked to report on their chorea symptoms for 6 months. For the in-clinic assessments, participants attended a clinic visit every 2 months and underwent an assessment of chorea severity using a seven-step structured test. The structured test was built into the mobile application and the tasks were performed (timed up and go, sit at rest with arms relaxed for 2 min, sit at rest with arms extended for 1 min, stand at rest for 30 s, walk in a straight line for 10 m, drink from a cup repetitively five times, and pronation-supination for 30 s). At home, patients were asked to rate their chorea severity using two methods. Using smartphone application, sit at rest with arms relaxed for 2 minutes and stand at rest for 30 seconds. The second method consisted of a single report of their current chorea severity at a predefined time. Triaxial accelerometer data were collected from both the wearable smartwatch and the smartphone. The patient’s smartwatch accelerometer data were collected continuously every day and the patient’s smartphone accelerometer and gyroscope data were collected during chorea assessments. | Arm chorea can be characterized using accelerometer data during static assessments. Patients completed 890 home assessments and provided 1,388 chorea reports following reminders in their home environment during the study period. A very high correlation (Pearson r = 0.99) was found between the patients’ mean chorea severity reports at home assessment and the chorea reporting following the home reminder. There was a correlation between the model chorea score (based on accelerometers data) and the patient-reported chorea score for the same assessment. There was also an overall trend of an increase in the model predicting chorea as the clinician’s report of chorea severity increased. |
| Adams, et al. Digit. Biomark., 2017 | Huntington disease (HD) | To evaluate the feasibility of capturing data from some sensors, characterize participant activities and survey participants on their experience | 1/ 15 (3)  2/ 5 (2) premanifest HD | 1/ 55 ± 10.7  2/ 38 ± 8.6 | 20 (14) | 58 ± 16.2 | 16 (7) patients with PD | 68 ± 8.7 | Tri-axial accelerometer (BioStampRC, by MC10 Inc.,Lexington, MA, USA) | 5 on each anterior thigh and forearm, and on the chest | Proportion of the day participants spent lying down, sitting, standing, and walking. | Participants were video-recorded undergoing standard clinical assessments including the Movement Disorder Society – Unified Parkinson Disease Rating Scale , the Unified Huntington Disease Rating Scale, Timed Up and Go, Ten-Meter Walk, and smartphone assessments. Following the visit, participants wore the sensors at home for an additional continuous interval totaling approximately 48 hours. During this time, participants filled out an activity diary. | Data were successfully obtained from 99.3% of sensors dispatched. On average, individuals with Huntington disease spent over 50% of the total time lying down, substantially more than individuals with prodromal Huntington disease, Parkinson disease, and controls. Most (86%) participants were “willing” or “very willing” to wear the sensors again. |
| Gil Polo, et al. Ann. Nutr. Metab., 2015 | Huntington disease (HD) | To analyze the energy expenditure in HD patients and compare their metabolic rates with healthy controls | 22(14) | 50.3 ± 15.6 | 18 (9) | 47.4 ± 13.8 |  |  | Tri-axial accelerometer (ActiGraph GT3X) | 1 on the waist | Total energy expenditure, physical activity (unspecified) | Data were recorded for 10 h. Patients were free to do the activities they usually practiced. The patients did not know when the devices were on. Food caloric energy intake (EI) and total energy expenditure (TEE) were considered for estimating energy balance (EB). A dietary recall questionnaire was used to assess the EI. TEE was computed as the sum of resting energy expenditure (REE), measured by indirect calorimetry. | TEE was significantly lower in patients compared to controls. PA was lower in patients compared to controls |
| Dinesh, et al. J. Huntingtons Dis., 2020 | Huntington disease (HD) | To measure motor symptoms of HD using wearable sensors in a longitudinal study | 1/ 15 (3)  2/ 5 (2) premanifest HD | 1/ 55.4 ± 10.7  2/ 38.5 ± 8.6 | 19 (13) | 57.6 ± 16.2 |  |  | Tri-axial accelerometers (BioStampRC® wearable sensors developed by MC10 Inc) | 5 on on each forarm and thigh and on the trunk | 1/ Truncal Chorea Index calculated based on the accelerometer data from the trunk when the participant was sitting.  2/ Gait parameters (step count, step duration, step length, walk speed, and coordination between the legs) 3/ Activity profiles | Participants underwent a baseline visit and were subsequently given the option to come for three follow up visits over the course of 12 months (at approximately 6 months, 9 months, and 12 months). During the visits, participants were video-recorded undergoing standard clinic assessments including the Unified Huntington Disease Rating Scale, Timed Up-and-Go, Ten-Meter Walk test, and smartphone assessments. After each visit, participants wore continuously the sensors at home for two days. | The average truncal Chorea Index was higher in individuals with HD (26.6, p < 0.001) than in controls (15.6). For participants with HD, the truncal Chorea Index showed a high intra-day variability but minimal change over 12 months. For the baseline visit, the Spearman correlation coefficient between the physician rated UHDRS maximal truncal chorea score and the average truncal Chorea Index was r = 0.52 (p = 0.06). Individuals with HD walked less (HD = 3818, prodromal HD = 6957, controls = 5514 steps/day) and took longer duration steps (HD = 0.97, prodromal HD = 0.78, controls = 0.85 seconds/step) than the other groups. Individuals with HD spent over half their day lying down (HD = 51.1%, prodromal HD = 38.0%, controls = 37.1%). |
| Andrzejewski, et al. J. Huntingtons Dis., 2016 | Huntington disease (HD) | To evaluate the feasibility and ability of wearable sensors to measure motor impairment in individuals with Huntington disease in the clinic and at home | 15 (6) | 56.8 ± 6.6 | 5 (3) | 53.4 ± 20.4 |  |  | Tri-axial accelerometer (PAMSys-XTM sensors (BioSensics, Cambridge, MA) | 5 on the chest and each ankle and wrist. Only the chest sensor was required for data analysis presented in this paper | 1/ time spent sitting, standing, walking, and running, the total number and duration of sit-to-stand and stand-to-sit transitions, and the total number of walking episodes, steps, and steps per walking episode.  2/ step time standard deviation, cadence, Step peak acceleration, medial-lateral speed, medial-lateral displacement | Participants were then fitted with five sensors and were video recorded while performing the UHDRS motor, Timed Up and Go, and Q-Motor assessments. At home, all five sensors were worn for 24 hours and then a replacement chest sensor was worn for an additional six days | Sensor data were successfully captured from 18 of the 20 participants at home. In the clinic, the standard deviation of step time (time between consecutive steps) was increased in Huntington disease compared to controls. At home with additional observations, significant differences were observed in seven additional gait measures. The gait of individuals with higher total motor scores (50 or more) differed significantly from those with lower total motor scores (below 50) on multiple measures at home. |
| McLaren, et al.  Neuropsychology, 2021 | Huntington disease (HD) | To investigate how sleep and physical activity habits related to cognitive functioning, in naturalistic settings, in early HD | 1/ 20  2/ 22 premanifest HD | 1/ 51.6 ± 8 2/ 42.1 ± 8.5 | 29 | 44.3 ± 10.9 |  |  | Triaxial accelerometer (Fitbit One) | 1 | Minutes sedentary, steps, activity calories | Participants wore Fitbit One sleep and activity monitors for 7 days and 7 nights. They used a smartphone application to complete daily sleep and activity diaries, sleep and mood inventories, and a brief battery of cognitive tests, which were completed on Day 8 of the study. | Groups were mostly similar across a range of Fitbit and self-report measures of sleep and physical activity, although the Manifest-HD group spent more time in bed than the Premanifest-HD and Healthy Control groups and had better self-reported sleep quality and more self-reported time spent sitting than the Healthy Control group and the Premanifest-HD group, respectively. There were no significant differences in Fitbit physical activity measures between the three groups. |
| Waddell, et al.  J. Huntingtons Dis., 2021 | Huntington disease (HD) | To assess feasibility of use and key features of HD in clinic and at home | 1/ 8 (4)  2/ 5 patients with premanifest HD (4) | 1/ 54.2± 7.2  2/ 38.5 ± 12.5 | 10 (5) | 57.4±12.7 |  |  | Android smartphone application :  1/ Tri-axial accelerometer and gyroscope data from the smartphone-embedded sensors for the chorea and gait and balance activities. 2/ Smartphone touchscreen sensor. |  | Chorea score (accelerometer data accumulated were divided into non-overlapping 2- s intervals. For each interval, a “chorea index” score normalized to 0–100 was estimated by computing the total variation in the band-pass filtered magnitude acceleration data), tap rate (estimated for each hand by dividing the total number of taps by the duration of the activity), step count, voice was assessed but not analysed | At home, participants were instructed to complete the activities thrice daily for one month. The GEORGE application included a set of four activities for which the following instructions were provided:  1/ Chorea activity (hold the phone in the hand with its screen facing upwards for 20 s in four different seated positions) 2/ Finger tapping activity (alternately tap your index and middle fingers as many times as possible in 20s)  3/Gait and balance activity (place the phone in your hip pocket and walk continuously for 30 s at your normal pace. After 30 s, turn in a full circle, then stand still for another 30 s)  4/ Voice activity (say “Aaaaah” into the microphone for as long as possible). | Participants used the application 2.1 times daily. Significant differences in chorea score (HD: 19.5; prodromal HD: 4.5, p = 0.007; controls: 4.3, p = 0.001) and tap rate (HD: 2.5 taps/s; prodromal HD: 8.9 taps/s, p = 0.001; controls: 8.1 taps/s, p = 0.001) between individuals with and without manifest HD were observed. Tap rate correlated strongly with the traditional UHDRS finger tapping score (left hand: r = – 0.82, p = 0.022; right hand: r = –0.79, p = 0.03). |
| Lang, et al. J. Huntingtons Dis., 2021 | Huntington disease (HD) | To find markers that reflect the prodromal phase in gene carriers | 1/ 12 (6)  2/ 7 (3) premanifest HD | 1/ 43.9 ± 13.2  2/ 41.4 ± 12.2 | 6 (4) | 52.17 ± 7.88 |  |  | A software developed by NeuraMetrix |  | This programme automatically separates the content of the written text from the information about consistency, the speed of movement of individual fingers, the dwell time of each finger on certain keys and the speed with which fingers of the right hand are moved in relation to fingers of the left hand - for right- or left-handed people. | Participants were asked to perform a software-supported, monitoring of keyboard typing on the participants’ computer at home for several months. Participants also undergo standard evaluation. | Statistically significant higher typing inconsistency was observed in HD patients compared to controls. Premanifest HD patients tended to show higher inconsistency levels than healthy controls. They found correlations between typing cadence and clinical scores (UHDRS finger tapping item, composite UHDRS and CAP score). |
| Cohen, et al. BMC Med. Inform. Decis. Mak., 2018 | Huntington disease (HD) | To understand patient compliance patterns in remote settings for four digital study protocol metrics | 17 | 51 ± 12 | 0 |  | 51 (22) patients with PD | 62 ± 11 | 1/ Pebble watch, iPhone 2/ Apple Watch, iPhone |  | Four compliance metrics specific to remote studies:  - smartphone app-based medication reporting - app-based symptoms reporting - the duration of smartwatch data streaming except while charging  - the performance of structured motor tasks at home. | Patient compliance was assessed in two remote, six-month clinical trials of PD and HD. Over 6 months, participants underwent app-based medication reporting, smartwatch data streaming, structured motor assessments, and app-based daily symptoms reporting | Compliance over time differed between the PD and HD studies, both studies maintained high compliance levels for their entire six month duration. None (− 1%) to a 30% reduction in compliance rate was registered for HD patients, and a reduction of 34 to 53% was registered for the PD study. Both studies exhibited marked changes in compliance rates during the initial days of enrollment. Daily smartwatch data streaming patterns were similar, peaking around noon, dropping sharply in the late evening hours and having a mean of 8.6 daily streaming hours for the PD study and 10.5 h for the HD study. Individual patients tended to have either high or low compliance across all compliance metrics. Predefined schedules and app-based reminders fulfilled their intended effect on the timing of medication intake reporting and performance of structured motor tasks at home. |
| Lipsmeier et al., J. Med. Internet Res., 2022 | Huntington disease (HD) | To evaluate a novel smartwatch- and smartphone-based digital monitoring platform to remotely monitor signs and symptoms of HD | 1/ 179 (72) 2/ 20 premanifest HD (10) | 2/ 44.9 ± 10.0 | 20 (7) |  |  |  | Roche HD Digital Monitoring Platform :  1/ smartwatch  2/ smartphone app | 1/ 1 on the wrist | Sway path, spiral drawing speed variability, median turn speed and step frequency variance | Data from 3 studies were used: the predrug screening phase of an open-label extension study evaluating tominersen and 2 untreated cohorts—the HD Natural History Study and the Digital-HD study. Patricipants completed 6 motor and 2 cognitive tests at home and in the clinic. To assess motor performance, the UHDRS Maximal Chorea item, UHDRS Finger Taps item, and UHDRS-TMS were used. A thome, the app requested the completion of active tests daily 79and subsequently recorded sensor data during daily living. Active tests included patient reported-outcome, cognitive tests, upper limb and gait tests (finger tapping, drawing, chorea evaluation, u-turn, balance, walking). | Participants in the open-label extension study, the HD Natural History Study, and the Digital-HD study completed 68-90% of the active tests, respectively. All sensor-based features showed good to excellent test-retest reliability (intraclass correlation coefficient 0.89-0.98) and generally low quality control failure rates. Good overall convergent validity of sensor-derived features to Unified HD Rating Scale outcomes and good overall known-groups validity among controls, premanifest, and manifest participants were observed. Among participants with manifest HD, the digital cognitive tests demonstrated the strongest correlations with analogous in-clinic tests (Pearson correlation coefficient 0.79-0.90). |
| Gaßner, et al. J. Neurol., 2020 | Huntington disease (HD) | To objectively identify characteristic features of gait in HD patients using sensor-based gait analysis | 43 (18) | 50.0 ± 11.1 | 43 (22) | 51.0 ± 11.3 |  |  | Tri-axial accelerometer and a tri-axial gyroscope (Wearable SHIMMER sensors, Shimmer Research Ltd., Dublin, Ireland) | 2 on the posterior portion of both shoes | Stride lenght, gait velocity, stride time, stance time. For each parameter, stride-by-stride variance is presented as the coefficient of variance (CV). | Participants performed a standardized 4 × 10 m overground gait test on a 10 m-long corridor in self-selected walking speed and without stops at turning points. Only straight strides were automatically detected by the stride detection algorithm and used for gait parameter calculations. Gait patterns were recorded by inertial sensors. Machine learning algorithms were applied to calculate spatio-temporal gait parameters and gait variability. | Stride length (− 15%) and gait velocity (− 19%) were reduced, while stride (+ 7%) and stance time (+ 2%) were increased in patients with HD. However, parameters reflecting gait variability were substantially altered in HD patients (+ 17% stride length CV up to + 41% stride time CV with largest effect size) and showed strong correlations to total motor score (TMS) and total functional capacity (TFC) (0.416 ≤ rSp ≤ 0.690). |
| Trojaniello, et al.  Gait posture, 2015 | Huntington disease (HD) | To test three methods, based on the use of a single IMU mounted at waist level, that have previoulsy been validated on healthy subjects on patients with pathological gait | 10 (5) | 50.3 ± 13.3 | 10 (6) | 69.7 ± 5.8 | 1/ 10 (2) hemiparetic patients  2/ 10 (5) patients with PD | 1/ 58.6 ± 12.1 2/ 73.8 ± 5.7 | Tri-axial accelerometer and a tri-axial gyroscope (OpalTM, APDM) | 1 on lumbar spine (between L4 and S2 | Initial and final foot contacts, referred to as gait events (GEs) and mean step length estimate. | Subjects were asked to walk back and forth for about 1 minute along a 12-m walkway with the instrumented mat. Subjects walked at self-selected, comfortable speed, wearing their own shoes. Walking aids such as canes or tripods were allowed if used in daily life. A single trial including several gait cycles was recorded for each subject. | Missed or extra events were found for all methods and a global decrease of their performance was observed to different extents depending on the specific group analyzed. None of the tested methods outperformed the others except the Parkinson’s disease subjects group for which one of the methods performed better than others. The hemiparetic subjects group was the most critical group to analyze (stride duration errors between 4-5 % and step duration errors between 8-13 %of the actual values across methods). |
| Purcell, et al. PLoS One, 2020 | Huntington disease (HD) | To investigate whether fast-paced and dual-task walking uncover deficits in gait and turning not seen under single-task, cognitive and gait outcomes relate to fall incidence, and gait deficits measured with wearable inertial sensors correlate with motor symptom severity in HD as measured by the Unified Huntington’s disease Rating Scale- total motor score | 17 (7) | 55 ± 9.7 | 17 (8) | 56.5 ± 9.3 |  |  | Tri-axial accelerometer and a tri-axial gyroscope (OpalTM wearable sensors) | 6 on the wrists, dorsum of feet, sternum and lower back (L5 level) | Cadence, stride length, gait speed, swing (% gait cycle), double support (% gait cycle), turn duration, number of steps to complete a turn, lateral step variability (m), which groups 3 consecutive steps and derives the extent of perpendicular deviation of the middle foot placement from the first and third step, stride length coefficient of variation (CoV), another common measure of gait variability, and step duration. The extent of DT interference, or the dual-task cost (DTC) in gait and turn performance was defined as DTC (%) = ((DT-ST)/ ST)*100 | Participants performed three, 2-minute walk tests on a 25-meter walkway under a self-selected (SS) pace, fast-as-possible (FAP) pace, and cognitive-motor DT condition (DT) at their normal pace. During the DT trial, participants were asked to perform an animal naming verbal fluency task, with the instruction that no animal could be repeated. | Four spatiotemporal gait parameters (gait speed, stride length, lateral step variability, and stride length variability) were consistently observed to be significantly different between the HD and control groups under all three conditions. Participant’s self-reported falls did not correlate with any gait or turn parameters. HD participants demonstrated significantly greater DTC for turning. |
| Dalton, et al. Gait Posture, 2013 | Huntington disease (HD) | To investigate the capacity of a single triaxial accelerometer sensor in detecting gait and balance impairments in pre-manifest and manifest Huntington’s disease subjects | 1/ 14 (6)  2/ 10 (6) premanifest HD | 1/ 51.83 ± 14.8  2/ 44.8 ± 11.7 | 10 (5) | 56.4 ± 10.9 years |  |  | Tri-axial accelerometer (AD_BRC sensor) | 1 on the thorax | Velocity, cadence, step length, step time, stride length | Participants was asked to perform five trials of walking on GAITRite system at their comfortable pace with all statistical analysis performed on respective mean values. They also performed the Romberg balance tests. An inverted pendulum model of the body’s centre of mass and an unbiased autocorrelation procedure were employed to derive gait parameters from the triaxial accelerometer signal. The accuracy of the gait measurements was compared to those recorded by a computerized walkway. | Strong agreement was seen between the sensor and the walkway; cadence (ICC = 0.95, CI = [0.75, 0.97]), velocity (ICC = 0.94, CI = [0.75, 0.97]) and step length (ICC = 0.89, CI = [0.77, 0.95]). Sensor derived velocity was significantly higher in healthy controls (p < 0.001) and PHD (p < 0.005) when compared to patients with HD. Step and stride length was significantly longer in healthy controls (p < 0.05) and patients with premanifest HD (p < 0.001) when compared to patients with HD. Significant diffences between subject groups across all four balance tasks (p < 0.001) were found. |
| Mannini, et al.  Annu. Int. Conf. IEEE Eng. Med. Biol. Soc. 2015 | Huntington disease (HD) | To obtain a general segmentation model, capable of dealing with all the available dataset groups | 10 (5) | 56.9 ± 12.6 | 10 (6) | 69.7 ± 5.8 | 10 (3) hemiparetic patients (3) | 64.5 ± 9.1 | Tri-axial accelerometer and a tri-axial gyroscope (Opal, APDM, Inc.) | 3 on both ankles and on lumbar spine, between L4 and S2. Only ankle sensors were considered in this study. | Gait event (foot strike and toe off) | Subjects were asked to walk back and forth for about one minute along a 12-meter walkway with the instrumented mat placed two meters from the starting line (GAITRiteTM Electronic Walkway). Subjects walked both at self-selected, comfortable speed and higher speed, wearing their own shoes. A single trial including several gait cycles was recorded for each subject. | This paper provide with a method to estimate gait event with shank-mounted sensors in heathly eldery, hemiparetic patient and patient with Huntinghton's disease. Effective stride segmentation was obtained across the different groups, despite the differences in signals morphology which are evident especially for hemiparetic patients at the impaired side. No missed cycles were observed and all spurious cycles were properly removed automatically. |
| Keren, et al. Front. Neurol., 2021 | Huntington disease (HD) | To evaluate daily-living gait quantity and quality in HD, taking into account irregular movements | 1/ For controlled environment : 42  2/ For non controlled environment : 29 (14 ) | 2/ 56.5 ± 11.9 | 1/ 14  2/ 22 (12) | 2/ 53.0 ± 9.6 |  |  | Tri-axial accelerometer (GENEActiv, Activinsights) | 1 on the wrist of the non-dominant hand | Walking time, steps, number of walking bouts per day, total daily-living activity index, cadence, dominant frequency, range, RMS, amplitude and width dominant frequency, step and stride regularity, step time variability and percentage of walking bouts with irregular movements. | Participants completed clinic-based assessments and a standardized laboratory-based circuit of functional activities, wearing inertial measurement units on the wrists, legs, and trunk. These activities were used to train and test an algorithm for the automated detection of walking. Some participants wore a tri-axial accelerometer on their non-dominant wrist for 7 days. | Measures of daily-living gait quantity including step counts, walking time and bouts per day were similar in HD participants and non-HD peers (p > 0.05). HD participants with higher clinician-rated upper body chorea had a greater percentage of walking bouts with irregular movements compared to those with lower chorea (p = 0.060) and non-HD peers (p < 0.001). Even after accounting for irregular movements, within-bout walking consistency was lower in HD participants compared to non-HD peers (p < 0.001), while across-bout variability of these measures was higher (p < 0.001). Many of the daily-living measures were associated with disease-specific measures of motor function. |
| Collett, et al. Gait Posture, 2014 | Huntington disease (HD) | To investigate gait variability and symmetry in HD using phase plot analysis | 1/ 28 (9) early stage 2/ 12(3) late stage 3/ 7 (5) premanifest HD | 1/ 47 ± 10  2/ 50 ± 14  3/ 48 ± 16 | 22 (11) | 46 ± 10 |  |  | Tri-axial accelerometer and a tri-axial gyroscope (LabVIEW2011, National Instruments, Ireland) | 1 on lower back (fourth lumbar vertebra) | 1/ Step time, cadence, stride lenght 2/ CoV (SD/mean x 100) was used to calculate variability in modelled spatiotemporal parameters.  5/ Phase plots considered the variability in consecutive wave forms from vertical movement and were quantified by SDA (spatiotemporal variability), SDB (temporal variability), ratio∡ (ratio SDA:SDB) and Δangleβ (symmetry). | For the walking assessment participants walked at their self-selected walking speed over a pre-set distance (8.8 or 10 m) whilst wearing the device to estimate centre of mass excursion. A subset of 28 individuals returned for a second assessment in order to investigate the stability of the phase plot measures. | Step time CoV was greater in manifest HD than controls, as was stride length CoV for late HD. No differences were found in spatiotemporal variability between premanifest HD and controls. Phase plot analysis identified differences between manifest HD and controls for SDB, Ratio∡ and Δangleβ . Furthermore Ratio∡ was smaller in premanifest HD compared with controls. Ratio∡ also produced the strongest correlation with UHDRS-TMS and was correlated with DBS. |
| Desai et al. Clin. Biomech. (Bristol, Avon), 2022 | Huntington disease (HD) | To evaluate postural control and gait in people with and without Huntington’s disease using wearable sensors; and to identify measures related to diagnosis and clinical severity | 43 (19) | 56.9 ± 11.7 | 15 (7) | 53.2 ± 13.2 |  |  | Inertial sensor (Opal sensors, ERT, Portland, OR, USA). | 6 | Postural measures included total sway area, root mean square (RMS) of sway, mean velocity, jerk and gait variable (gait speed, cadence, percentage of time spent in stance and double support, and stride length) | Participants performed standing with feet together and feet apart, sitting, and walking with wearable inertial sensors. One-way analysis of variance determined differences in measures of postural control and gait between early and mid-disease stage, and non- Huntington’s disease peers. A random forest analysis identified feature importance for Huntington’s disease diagnosis. Stepwise and ordinal regressions were used to determine predictors of clinical chorea and tandem walking scores respectively. | Root mean square measures obtained in sitting and standing using wearable sensors have the potential to serve as biomarkers of postural control impairments in Huntington’s disease |
| Muratori, et al. Hum. Mov. Sci., 2021 | Huntington disease (HD) | To evaluate if dual-task postural sway would be a more sensitive marker of instability in people with HD than dual-task gait | 43 (18) | 53.6 ± 11.6 | 15 (7) | 52.2 ± 13.2 |  |  | Inertial sensors (APDM Opal,Portland, OR) | 6 on both wrists, mid-chest, lumbar spine, and both feet | Root mean square (RMS) of postural sway | Participants completed single tasks of walking (Timed Up & Go, 7 m walk), standing (feet together, feet apart and foam surface) and seated cognitive performance (Stroop, Symbol Digit Modalities Test, Delis-Kaplan Executive Function System Sorting test and dual cognitive-motor tasks while standing) and walking (+ DKEFS, TUG cognitive). Opal sensors recorded measures of postural sway and time to complete motor tasks. | Individuals with HD had a greater increase in standing postural sway compared to HC from single to dual-tasks and with changes to support surface. Both groups demonstrated a decrease in gait performance during the TUG cognitive, however, this difference was greater in people with HD compared to HC. While those with HD showed a greater dual-task motor cost compared to HC, both groups behaved similarly as condition complexity increased. |
| Porciuncula, et al. Neurorehabil. Neural Repair 2020 | Huntington disease (HD) | To examine postural control in patients with HD and premanifest HD | 1/ 11  2/ 17 premanifest HD | 1/ 50 ± 11.8  2/ 41.1 ± 9.3 | 11 | 43.8 ± 11.4 |  |  | Triaxial inertial sensor (APDM, Eugene, OR) | 1 on lower back (L5 segment) | Jerk in anteroposterior and mediolateral, as the time derivative of acceleration and refers to smoothness of sway. Total sway area is the 2-dimensional planar sway area in the transverse plane, computed as the area included in acceleration per unit time. Total power in anteroposterior and mediolateral directions refers to total power of a specific plane and provides a measure of the frequency of sway | Using wearable sensors, postural control was assessed according to (1) postural set (sit vs stand), (2) sensory attenuation using clinical test of sensory integration, and (3) sensory enhancement with gaze fixation. | Patients with premanifest HD reduced postural sway in sitting relative to standing, whereas patients with HD had pronounced sway in standing and sitting, highlighting a baseline postural deficit. During sensory attenuation, postural control in patients with premanifest HD deteriorated relative to controls when proprioceptive demands were high (eyes closed on foam), whereas patients with HD had significant deterioration of postural control when proprioception was attenuated (eyes open and closed on foam). Finally, gaze fixation improved sway smoothness, amplitude, and frequency in premanifest HD; however, no benefit was observed in HD. All mesures except for total power AP had good reliabilities. |
| Kegelmeyer, et al. J. Neurol. Sci., 2017 | Huntington disease (HD) | To compare trunk control in sitting, standing and walking between individuals with HD and controls; and to examine the ability of individuals with HD to alter pelvic positioning in response to biofeedback as a potential training technique to improve postural stability | 41 (21) | 52.2 ± 10.6 | 36 (20) | 45.9 ± 14.5 |  |  | Gyroscope and accelerometer (iPod) | 2 on lower back (L5 vertebral level) and on lower border of the scapulae | For thoracic excursion and pelvic excursion :  peak anterior, peak posterior, peak right, peak left, total sagittal, total frontal, mean sagittal and mean frontal | Participant performed two trials of each of the following activities: sitting on a standard height chair, standing with feet together and arms by their sides for 1 min ans walking for 14 meters at their self-selected walking speed over a GAITRite® electronic walkway. | Amplitude of thoracic and pelvic trunk movements was significantly greater in participants with HD, and differences were more pronounced during static (i.e. sitting, standing) than dynamic (i.e. walking) tasks. In contrast to the slow, smooth sinusoidal trunk movements of controls, individuals with HD demonstrated rapid movements with varying amplitudes that continuously increased without stabilizing. 97% of participants with HD were able to modify their trunk position in response to auditory cues. |
| Desai, et al. Gait Posture, 2021 | Huntington disease (HD) | To determine inertial measurement unit-derived measures of anticipatory postural adjustments (APA) acceleration amplitudes and durations, and first step range of motion (ROM) and durations as previously identified via force plate measures, in HD and controls | 33 |  | 15 |  |  |  | Tri-axial accelerometer and a tri-axial gyroscope (Opal, APDM, Inc., Portland,OR) | 3 on the dorsum of the feet bilaterally and on the lumbar region | APA acceleration amplitude, APA durations, first step range of motion (ROM), first step durations (and relationship to gait speed) | Participants wore the IMU during a 14-meter walk under no load and cognitive load conditions. Participants started walking at the sound of a tone, walked to a 7-meter mark, turned and walked back to the start, completing 14 meters. The walking condition consisted solely of the 14-meter walk (no-load). For the walking condition with an added cognitive load, participants were instructed to begin walking while performing the D-KEFS verbal fluency task with category switching. | Individuals with HD had greater APA acceleration amplitudes, smaller first step ROM and longer first step durations compared to non-HD peers. No differences in APA durations were present between groups in both conditions. Cognitive loading influenced first step ROM but not other APA parameters. Mediolateral APA acceleration amplitudes were a significant predictor of gait speed and were related to disease-specific measures. |
| Bennasar, et al. IEEE Trans. Neural Syst. Rehabil. Eng., 2018 | Huntington disease (HD) | To develop and validate a novel low cost, objective automated system for the evaluation of upper limb movement impairment in HD in order to eliminate the inconsistency of the assessor and offer a more sensitive, continuous assessment scale | 44 |  | 48 |  |  |  | Tri-axial accelerometer (GENEactiv accelerometers, Activinsights, UK) | 3 on each wrist and on the sternum | Captured acceleration data were used to develop an automatic classification system for discriminating between healthy and HD participants and to automatically generate a continuous movement impairment score (MIS) that reflected the degree of the movement impairment. | Participants completed the Money Box Test (MBT) a functional upper limb dexterity assessment involving a series of token transfer tasks wearing the device. | The developed system achieved 98.78% accuracy in discriminating between healthy and HD participants. The Pearson correlation coefficient between the automatic MIS and the clinician rated mULMS was 0.77 with a p-value < 0.01. The approach presented in this paper demonstrates the possibility of an automated objective, consistent, and sensitive assessment of the HD movement impairment. |
| Mannini, et al. Sensors (Basel), 2016 | Huntington disease (HD) | To lay the methodological foundation for mobile-based gait assessment tools | 17 (7) | 54.3 ± 12.2 | 10 (6) | 69.7 ± 5.8 | 15 (5) post stroke patients | 61.3 ± 13 | Tri-axial accelerometer and a tri-axial gyroscope (Opal, APDM, Inc., Portland, OR, USA) | 3 on both shanks and on lumbar spine (between L4 and S2) | The complete features data set hence included 90-dimensional feature vectors (six time domain features for each channel, six frequency domain features for each channel and six HMM-based features). | A 7-meters long instrumented gait pressure mat (GAITRiteTM Electronic Walkway) was used to acquire reference data. The instrumented mat returned the foot strike (FS) and toe off (TO) events and all relevant gait temporal parameters. The IMUs and the instrumented mat were synchronized by means of a wired connection.  The number of passages on the instrumented mat varied from subject to subject from a minimum of two passages to a maximum of 16. | The 90.5% of subjects was assigned to the right group after leave-one-subject–out cross validation and majority voting. |
| Lauraitis, et al. J. Healthc. Eng., 2018 | Huntington disease (HD) | To create a computerized behavioural model, which predicts an impaired reaction condition for HD patients | 10 |  | 10 |  |  |  | Any mobile devices that support Android OS |  | The subject’s reaction time (rt) and the Euclidian distance between the two points of true and touched positions (delta) | The mobile application randomly generates circular shape objects (2, 3, and 5 circles at time) of particular color that are generated on the mobile device’s screen. An active circle that needs to be touched is marked by a black contour so as to differ from other objects. The subjects are instructed to touch every object, starting from first in sequence, by finger as close to center and as quickly as possible. When subject finishes the test, collected data is stored in external mobile device storage and sent to the database using the internet connection. | The feed-forward backpropagation (FFBP) neural network achieved the regression R value of 0.98 and mean squared error (MSE) values of 0.08, while the fuzzy logic system (FLS) provides a final evaluation of subject’s reaction condition in terms of functional capacity level. |
| Shih, et al. medRxiv, 2022 | Huntington disease (HD) | To evaluate the feasibility of using wearable activity monitors as part of a physical  activity (PA) intervention and describe patterns of device weartime and PA behavior in people with Parkinson and Huntington disease. | 14 |  |  |  | 13 patients with PD |  | Fitbit | 1 on the wrist | Average steps MET*min per week | Secondary analyses were conducted on two pilot studies. Participants enrolled in a 4-month physical activity coaching program. Participants wore a Fitbit Charge 2 and physical and occupational therapists guided them through understanding of PA data and goal-setting to build autonomy and facilitate PA uptake. | Retention rate of the intervention was 85%. Participants had a mean (SD) of 92.3(9.2)% valid wear days over the intervention period. Regardless of diagnosis, day & night Fitbit wearers had more improvements in steps (d=1.02) and MET*min/week (d=0.69) compared to day-only wearers. There was a small effect of increased steps per day in participants with PD and decreased steps per day in participants with HD from baseline to follow-up. |
| Raccagni, et al. Brain Behav.,2018 | Progressive supranuclear palsy (PSP) | To identify quantitative gait parameter differences in PD and in atypical PD patients using sensor-based gait analysis and to correlate gait parameters with clinical rating scales | 12 (3) | 67.4 ± 8.7 | 25 (12) | 63.7 ± 9.7 | 1/ 25 (12) patient with PD  2/ 13(9) patient with MSA | 1/ 66.6 ± 7.9  2/ 63.5 ± 8.5 | Three-axial accelerometers and gyroscope (SHIMMER 2 sensors, Shimmer Research Ltd., Dublin, Ireland) | 2 on the posterior lateral portion of both shoes | Gait speed, stride length, cadence, maximum toe clearance. These parameters were normalized to the height of the participants. | Patients were assessed using standardized clinical rating scales (MDS-UPDRS-3, UMSARS, PSP-RS). Gait analysis consisted of inertial sensor units generating gait parameters from 4 × 10 m walk tests. | Gait speed was significantly reduced in patients with PD compared to controls and even more in atypical PD. Similar results were obtained for stride length. The maximum toe clearance and heel strike angle, toe off angles were significantly impaired in PD and atypical PD patients compared to controls but did not reveal a significant difference between both patient cohorts. Clinical ratings significantly correlated with gait speed and stride length in atypical PD patients. |
| Hatanaka, et al. Eur. Neurol., 2016 | Progressive supranuclear palsy (PSP) | To evaluate the gait characteristics of PSP patients in comparison to PD patients and controls using a portable triaxial accelerometer rhythmogram, and attempted to better clarify the disease-specific differences in gait performance between PSP and PD | 20 (14) | 71.8 ± 5.9 | 24 (5) | 73.7 ± 3.8 | 124 (64) patients with PD | 68.4 4 ± 11.2 | Tri-axial accelerometer rhythmogram device (Mimamori-Gait® System, LSI Medience Corp., Tokyo, Japan). | 1 on the back of the waist. | Double support time, step time, cadence and velocity | Patients walked at most comfortable speed without any secondary tasks in a 16-m long and 2-m wide hallway that was well-lit, flat, and free of obstacles. | Both PSP and PD patients shared the following similar hypokinetic gait characteristics: decreased velocity, step length, cadence and mean acceleration. Step time and variability in step time were mutually related. However, among the 3 groups, PSP patients showed characteristically low vertical displacement and a higher acceleration than PD patients at the same cadence. |
| Baston, et al. Gait Posture, 2014 | Progressive supranuclear palsy (PSP) | To introduce an instrumented easy-to-use method to measure postural strategy | 7 (3) | 68 ± 5 | 7 (4) | 68 ± 7 | 5 (1) patients with PD | 62 ± 6 | Tri-axial accelerometer (Opal inertial sensors (ADPM Inc., Portland, OR) | 2 on the trunk at L5 level and on the right shank | Covariance index over time (by a sliding-window algorithm, representing the coordination between the upper and lower segments of the body during postural sway), postural strategy index and the amount of postural sway (as adjunctive information to characterize balance, by the root mean square of the horizontal trunk acceleration signal) | Participants were asked to stand quietly on a moveable plate (Neurocom Balance Master, Neurocom, Clackamas, OR), secured in a safety harness during the Sensory Organization Test. All participants were assessed during 6 sensory conditions in 3 consecutive trials of 20 s each: condition 1 (eyes open), condition 2 (eyes closed), condition 3 (sway referenced visual surround) with a stable base and condition 4 (eyes open), condition 5 (eyes closed), condition 6 (sway- referenced visual surround) with a moveable base (sway referenced). | This study showed that control subjects were able to change their postural strategy, whilst PSP and PD subjects persisted in use of an ankle strategy in all conditions. PD subjects had root mean square values similar to control subjects even without changing postural strategy appropriately, whereas PSP subjects showed much larger root mean square values than controls, resulting in several falls during the most challenging sensory organization test conditions. |
| Pilotto, et al. Cerebellum, 2021 | Progressive supranuclear palsy (PSP) | To test the efficacy of theta burst repetitive transcranial magnetic stimulation (rTMS) on postural instability in PSP | 20 (7) | 74 ± 4 |  |  |  |  | Tri-axial accelerometer and gyroscope (Rehagait®, Hasomed GmbH, Magdeburg, Germany) | 1 on the lower back (third lumbar spine) | Extracted sway parameters: Area, mean velocity, mean acceleration (root mean square - RMS), jerk (indicating smoothness of compensatory movements), mean frequency, mean velocity | Participant underwent a clinical evaluation including the Tinetti test, the Short Physical Performance Battery (SPPB), the Timed Up and Go test and the Functional Reach test (FR) wearing the inertial sensor before and after stimulation. | Active stimulation was associated with increase in time without falls. In the same tasks, device-extracted parameters revealed significant improvement in area, velocity, acceleration and jerkiness of sway in real versus sham stimulation. Cerebellar rTMS showed a significant effect on stability in PSP patients, when assessed with mobile digital technology, in a double-blind design. |
| Djuric-Jovicic, et al. J. Clin. Neurosci., 2016 | Progressive supranuclear palsy (PSP) | To investigate repetitive finger tapping patterns in patients with PD, PSP and MSA | 15 (6) | 65.8 ± 8.7 | 14 (8) | 56.8 ± 9 | 1/ 14 (9) patients with MSA  2/ 13 (6) patients with PD | 1/ 58 ± 4.5  2/ 60.9 ± 9.9 | Inertial sensor | 2 on the thumb and index finger | Angle amplitude in degrees, cycle duration, speed, coefficients of variation (CV) of amplitude and coefficient of variation (CV) of speed | The participants were asked to sit comfortably and were asked to hold their hand in front of them. Participants were instructed to repeatedly tap the index finger and thumb as rapidly and as widely as possible for 15 seconds with a 1 minute pause between trials. Each of the three consecutive trials began and ended with fingers closed (zero angle). | The lack or only minimal progressive reduction in amplitude during the finger tapping in PSP patients, similar to HC, but significantly different from the sequence effect (progressive decrement) in both PD and MSA patients. No significant differences were found in the average finger separation amplitudes between PD, PSP and MSA patients The lack of clinically significant sequence effect during finger tapping differentiated PSP from both PD and MSA patients, and might be specific for PSP. |
| De Vos, et al. Gait Posture, 2020 | Progressive supranuclear palsy (PSP) | To record data during commonly applied gait-related tasks and explore how well the data can distinguish PSP from PD and from healthy control (HC) participants. To analyse which sensors/body locations are necessary to acquire this data, to determine how far the sensor array can be simplified while still yielding satisfactory results | 21 (9) | 71 | 39 (20) | 67.1 | 20 (9) patients with PD | 66.4 | Tri-axial accelerometer, gyroscope, and magnetometer (OpalTM, APDM, Portland, USA) | 6 on the lumbar spine, sternum, wrists and feet | The software automatically extracts a range of clinical features specific to the three tasks. The full feature set included 109 parameters from analysis of the gait task, 33 from the sway test, and 14 from the TUG. | Participants performed a two minute walk, static sway test, and timed up-and-go task, while wearing an array of six inertial measurement units. The data were analysed to determine what features discriminated PSP from PD and PSP from HC. Two machine learning algorithms were applied, Logistic Regression (LR) and Random Forest (RF). | 17 features were identified in the combined dataset that contained independent information. The RF classifier outperformed the LR classifier, and allowed discrimination of PSP from PD with 86 % sensitivity and 90 % specificity, and PSP from HC with 90 % sensitivity and 97 % specificity. Using data from the single lumbar sensor only resulted in only a modest reduction in classification accuracy, which could be restored using 3 sensors (lumbar, right arm and foot). For maximum specificity the full six sensor array was needed. |
| Sotirakis, et al. Movement Disorders, 2022 | Progressive supranuclear palsy (PSP) | To establish whether data collected by a body-worn inertial measurement unit (IMU) network could predict clinical rating scale scores in PSP and whether it could be used to track disease progression | 17 (8) | 63 |  |  |  |  | Inertial sensor (Opal, APDM, Portland, OR, USA) | 6 on the left and right wrists/feet, the sternum, and the lumbar region | 150 gait and posture features. Selection of the 3 best progression feature : mean toe off angle, mean turn velocity, standard deviation of stride length | Participants underwent five visits at 3-month intervals. Participants performed a 2-minute walk and an assess- ment of postural stability by standing for 30 seconds with their eyes closed, while wearing the device. | A simple linear regression model incorporating the three features with the clearest progression pattern was able to detect statistically significant progression 3 months in advance of the clinical scores. A more complex linear regression and a random forest approach did not improve on this. |
| Panyakaew, et al. J. Neurosci., 2020 | Focal Dystonia (FD) | To examine the neurophysiological  characteristics of dystonic tremor syndrome and its subtypes in comparison with essential tremor | 22 (10) | 59.0 ± 10.3 | 19 (8) | 55.8 ± 9.3 | 21 (8) patients with essential tremor | 56.1 ± 8.9 | 1/ Tri-axial accelerometers 2/ Bipolar surface EMG electrodes | 1/ 2 on the dorsum of both hands 2/ 4 on the flexor carpi radialis and extensor carpi radialis muscles bilaterally | Peak frequency (Fp), Half-width power (HWP) meaning the area under the curve between two vertical straight lines at the rising and falling edge of the peak at half peak power, full-width half maximum (FWHM), tremor stability index (TSI) calculated as the interquartile range of change in frequency, computed from the relationship between instantaneous variation in frequency and instantaneous frequency), intermuscular coherence magnitude (CohM), intermuscular coherence phase (CohPh). | Tremors were analyzed during posture, simple kinetic movement, and writing. Cerebellar inhibition was performed by transcranial magnetic stimulation. Severity of tremor and its impact The Essential Tremor was assessed using the Rating Assessment Scale (TETRAS). | Dystonic tremor (DT) exhibited higher variability of peak frequency and greater instability of tremor burst intervals over time than essential tremor (ET) or tremor  associated with dystonia regardless of tasks (TAWD). Intermuscular coherence magnitude between the antagonist pairs increased during the writing task in DT, but not ET or TAWD. ET and TAWD exhibited different phase relationships of  the temporal fluctuations of voluntary movement and tremor in the kinetic condition. A linear  discriminant classifier based on these tremor parameters was able to distinguish the three  groups with a classification accuracy of 95.1%. Cerebellar inhibition was significantly reduced in DT, but not in TAWD, compared to ET and healthy controls. Our study shows that the two DTS are distinct entities with DT closer to non-tremorous dystonia and TAWD closer to ET. |
| Delrobaei, et al. J. Neuroeng. Rehabil., 2016 | Focal Dystonia (FD) | To define the kinematic and kinetic characteristics of writer’s cramp at multiple joints, across forces and angles at these joints and to study the relationship between changes in the angles of the writing surface and the kinematics of cramping | 9 (3) |  |  |  |  |  | 1/ Goniometer (NexGen Ergonomics Inc., Pointe Claire, Quebec) 2/Single axis torsiometer (NexGen Ergonomics Inc., Pointe Claire, Quebec) 3/ FlexiForce® sensors (NexGen Ergonomics Inc., Pointe Claire, Quebec) placed on a pen.  4/ A pressure-sensitive writing surface (ATI Industrial Automation, Inc., Apex, California) | 1/ 3 sensors : 1 twin axis goniometer on the wrist, 1 single axis goniometer attached on the elbow and 1 twin axis goniometer attached on the shoulder. 2/ 1 sensor on the forearm. 3/ 2 on a pen | Nine kinetic-kinematic measures : thumb, index and hand force; wrist flexion-extension, ulnar-radial deviation, pronation-supination, elbow flexion-extension, shoulder flexion-extension and abduction-adduction | Participants performed a series of 10 standard scripted writing and drawing tasks while wearing the kinematic sensors. All tasks were performed on a horizontal surface as well as on three tilted surfaces. | Four tasks appeared to best predict cramp occurrence. Unique biomechanical profiles emerged for patients regarding force, angles and cramp severity. Cluster analyses using these features showed a clear separation of patients into two severity classes. Finally, a relationship between severity and kinetic-kinematic information suggested that primary cramping versus compensatory movements could be potentially inferred. . |
| Rudzińska, et al. Neurol. Neurochir. Pol., 2013 | Focal Dystonia (FD) | To assess the incidence and to characterize parameters of hand tremor accompanying focal and segmental dystonia | 123 |  | 51 |  |  |  | 1/ Graphic tablet 2/ Three-axial accelerometer (BIOPACK) 3/ Surface electromyography. | 2/ 1 on the proximal phalanx of the third finger 3/ 2 on wrist flexors and extensors | Frequency and occurrence of tremor | Clinical and instrumental assessment of upper limb tremor was performed consecutively in both hands with the use of instruments (accelerometer, electromyography, graphic tablet). Frequency and severity of tremor were assessed in three positions: at rest; with hands extended; during ‘finger-to-nose’ test and during Archimedes spiral drawing. The severity of dystonia was assessed with the Toronto Western Spasmodic Torticollis Rating Scale (TWSTRS); Jankovic Rating Scale for the assessment of the severity of blepharospasm; Function Scale and Burke-Fahn-Marsden Evaluation Scale. Clinical evaluation of tremor severity was made using the Simple Tremor Severity Scale. | The incidence of tremor was significantly higher in dystonic patients as compared to controls (p = 0.0001). In clinical examination, tremor was found in 50% of dystonic patients, and in instrumental assessment in an additional 10-20%. The most frequent type of tremor was postural and kinetic tremor with 7 Hz frequency and featured essential tremor type. In the control group, tremor was detected in about 10% of subjects as 9-Hz postural tremor of enhanced physiological tremor type. No differences were found between patients with different types of dystonia with respect to the tremor incidence, type and parameters (frequency and severity). No correlations between tremor severity and dystonia severity were found either. |
| Nieuwhof, et al. Neuroimage Clin., 2022 | Focal Dystonia (FD) | To investigate the contribution of the cerebello-thalamo-cortical circuit and the basal ganglia to the pathophysiology of dystonic tremor syndrome | 27 (14) | 62.0 ± 12.7 | 27 (14) | 61.0 ± 11.5 |  |  | Tri-axial accelerometer (Brain ProductsAcc) | 1 on the dorsum of the hand or on one of the fingers | Tremor power | Participants performed concurrent recording with accelerometery and functional MRI during a posture holding task that evoked tremor, alternated with rest. Multiple regression analyses was used to separate tremor-related activity from brain activity related to (voluntary) posture holding. | Tremor-related activity in sensorimotor regions of the bilateral cerebellum, contralateral posterior and anterior ventral lateral nuclei of the thalamus (VLp and VLa), contralateral primary motor cortex (hand area), contralateral pallidum, and the bilateral frontal cortex (laterality with respect to the tremor). Grey matter volume was increased in patients compared to controls in the portion of contralateral thalamus also showing tremor-related activity, as well as in bilateral medial and left lateral primary motor cortex, where no tremor- related activity was present. Effective connectivity analyses showed that inter-regional coupling in the cerebello-thalamic pathway, as well as the thalamic self-connection, were strengthened as a function of increasing tremor power. |
| Elble, et al. Mov. Disord. Clin. Pract., 2017 | Focal Dystonia (FD) | To figure out which one of gyroscopy or accelerometry is superior to assess head tremor. To study the relationship between 0 to 4-point tremor ratings and the transducer measures of tremor amplitude | 17 (15) | 61 ± 9 |  |  | 9 (2) patients with essential tremor | 74 ± 5 | Tri-axial accelerometer and a tri-axial gyroscope (Kinesia motion sensor, Great Lakes Neurotechnologies, Cleveland, OH) | 1 on a hat | Tremor frequency, mean peak-to-peak tremor displacement or rotation over the entire duration of each 1-minute recording (“mean tremor amplitude”), mean peak-to-peak tremor amplitude over the 3-second interval with greatest tremor during each recording (“maximum burst amplitude”) | Head tremor was recorded while subjects sat in a chair with the head in each of five positions used in The Essential Tremor Rating Assessment Scale (TETRAS). Each head position was held for 1 minute while tremor was recorded with the transducers. During the recording, a movement disorders specialist rated head tremor using the Fahn-Tolosa-Marin. | Gyroscopic transducers are superior to accelerometry for assessment of head tremor. Both measures of head tremor are logarithmically related to tremor ratings. Minimum detectable change (percent reduction) was approximately 66% of the baseline geometric mean. That is is comparable to those previously reported for hand tremor. |
| Celletti, et al. Clin. Biomech. (Bristol, Avon), 2021 | Focal Dystonia (FD) | To investigate whether dystonic posture in patients with cervical dystonia affects walking and causes postural changes | 22 (16) | 62.4 ± 11.1 |  |  | 16 (8) patients with essential tremor | 60.8 ± 8 | Triaxial accelerometer, triaxial magnetometer, triaxial gyroscope (G-Sensor, BTS SpA, Milano, Italy) | 1 on the lower back (L5) | Total duration, Sit-to-Stand Duration, AP acceleration, L acceleration, V acceleration, Stand-to-Sit Duration, Mid-Turning Duration, peak velocity, Mean velocity, Final-Turning Duration | Participants underwent an instrumental evaluation of the Timed Up and Go Test. | All the spatio-temporal parameters of the sub-phases of the Timed up and go test had a significantly higher duration in cervical dystonia patients compared to the control group while no differences in flection and extension angular amplitudes were observed. Indeed, we found that Cervical Dystonia patients had abnormalities in turning, as well as in standing-up and sitting-down from a chair during the Timed up and go test than healthy controls. |
| Park, et al. J. Medical and Biological Eng., 2019 | Focal Dystonia (FD) | To provide an objective evaluation of the severity of cervical dystonia based on three-dimensional kinematics of the neck using wearable inertial sensors | 8 (4) |  |  |  |  |  | Tri-axial accelerometer and a tri-axial gyroscope and tri-axial magnetometer (EBIMU 9DOF, E2BOX, Inc., Seoul, Republic of Korea) | 2 on the anterior chest and head | Mean and peak values of rotation angle (RA) and magnitude of angular velocity (MAV) | Patient was recorded while sitting with inertial sensors and camera evaluation. Instrumented outcomes were compared with Toronto Western Spasmodic Torticollis Rating Scale (TWSTRS) scores. | The MAV parameters showed a higher correlation with clinical severity than RA parameters. The intraclass correlation coefficients of the four parameters between the test–retest were more than 0.9. |
| Zhang, et al. Ann. Clin. Transl. Neurol., 2022 | Focal Dystonia (FD) | To evaluate a novel software system to quantify multi-axis directionality and severity of head posture in CD using only conventional video camera recordings | 185 (137) | 44.0 ± 12.0 |  |  |  |  | The Computational Motor Objective Rater (CMOR) is based on computer vision and machine learning technology that captures 3D head angle from video. |  | Angle of head deviation in 3 axis | CMOR was used to quantify the axial patterns and severity of predominant head posture in a retrospective, cross-sectional study. | The predominant head posture involved more than one axis in 80.5% of patients and all three axes in 44.4%. CMOR’s metrics for head posture severity correlated with severity ratings from movement disorders neurologists using both the TWSTRS-2 and an adapted version of the Global Dystonia Rating Scale ( all p <0.001). |
| Mason, et al. Trem. Other Hyperkinet. Mov. (NY), 2022 | Focal Dystonia (FD) | To characterize and quantify the effect of Deep Brain Stimulation (DBS) using markerless-3D-kinematics combined with accelerometry | 1 (0) | 56 |  |  |  |  | Accelerometer (PhoneXS, Apple Inc.) | 1 on the head at the right temple | Total tremor amplitude and frequency (estimated as the single peak frequency derived from a Fast Fourier transform) | Stereo videography was used to record the participant in 3D. The DeepBehavior toolbox was applied to obtain timeseries of joint position for kinematic analysis. Accelerometry was performed simultaneously for comparison. | Bilateral Vim DBS improved both dystonic tremor magnitude and tonic posturing. DBS of the hemisphere contralateral to the direction of dystonic head rotation (left Vim) had greater efficacy. Assessment of tremor magnitude by 3D-kinematics was concordant with accelerometry and was able to quantify dystonic posturing. |
| Hickey, et al. Physiol. Meas., 2016 | Spinocerebellar ataxia (SCA) | To examine the validity of a single wearable for deriving 14 spatio-temporal gait characteristics in SCA6 and control cohorts | 22 (15) | 57.2 ± 12.8 | 23 (15) | 51.3 ± 12.3 |  |  | Tri- axial accelerometer-based wearable (Axivity AX3; Axivity, York, UK) | 1 on the lower back (L5) | Mean and variability of step velocity, length, and step, swing and stance time; and the asymmetry of step length, and step, swing and stance time | Participants performed eight intermittent walks along a 7 m instrumented walkway at their preferred walking pace while wearing the accelerometer. | Mean gait characteristics showed good to excellent agreement for both groups, although gait variability and asymmetry showed poor agreement between the two systems. Agreement improved considerably in the SCA 6 group when people who used walking sticks were excluded from the analysis, suggesting poorer agreement in people with more severe gait impairment. Despite poor agreement for some characteristics, gait measured using the wearable was generally more sensitive to group differences than the instrumented walkway. |
| Shah, et al. IEEE Trans. Biomed. Eng., 2020 | Spinocerebellar ataxia (SCA) | To validate and determine the generalizability of two algorithm (discrete Turn Algorithm for variable and sequential turns close in time and Merged Turn Algorithm for a single turn angle in the presence of hesitations) | 51 | 54.5 ± 13.3 | 50 | 55.8 ± 14.8 |  |  | Triaxial accelerometers, gyroscopes, and magnetometers (Opals by APDM Wearable Technologies, Portland, OR, USA) | 6 on both feet, wrists, the sternum, and lumbar regions. They used only feet, sternum, and lumbar sensor data in this analysis. | Turn angle, turn Duration, steps in Turn, turn Rate Average, turn Rate Max and Turn Hesitations | The SCA/HC Study Protocol: participants were asked to perform a 2-min walk test (subjects were instructed to walk at a comfortable pace back and forth continuously between two lines on the floor 7.5 m apart for 2 min). | The Discrete Turn Algorithm shows improved agreement with optical motion capture and with known turn angles, compared to our previous algorithm by El-Gohary et al. The Merged Turn algorithm that merges consecutive turns in the same direction with short hesitations resulted in turn angle estimates closer to a fixed 180-degree turn angle in the PD, SCA, and controls subjects compared to a previous turn algorithm. Additional metrics were proposed to capture turn hesitations in PD and SCA. |
| Kanzler, et al. Ann. Clin. Transl. Neurol., 2022 | Spinocerebellar ataxia (SCA) | To unravel quantitative motor biomarkers in degenerative ataxias in real-life turning movements that are sensitive for changes both longitudinally and at the preataxic stage. | 22 + 8 premanifest SCA | 51 ± 15 | 23 | 48 ± 15 |  |  | Inertial sensor (OpalTM, APDM, Portland, Oregon) | 3 on both feet and L5 | Mean velocity, duration, angle, number of steps, lateral velocity change (LVC), Outward acceleration, inward acceleration | Participants underwent a cross-sectional and longitudinal observational study.Turning movements were assessed by three body-worn inertial sensors in three conditions: (1) instructed laboratory assessment, (2) supervised free walking, and (3) unsupervised real-life movements (total recording time per subject: 4–6 hours). | Measures that quantified dynamic balance during turnin (lateral velocity change (LVC) and outward acceleratio) but not general turning measures such as speed, allowed differentiating ataxic against healthy subjects in real life (effect size δ = 0.68), with LVC also differentiating preataxic against healthy subjects (δ = 0.53). LVC was highly correlated with clinical ataxia severity (scale for the assessment and rating of ataxia [SARA] score, (effect size ρ = 0.79) and patient reported balance confidence (ρ = 0.66). Moreover, LVC in real life—but not general turning measures or the SARA score—allowed detecting significant longitudinal change in 1-year follow-up with high effect size/ |
| Jin, et al. Artif. Intell. Med., 2020 | Spinocerebellar ataxia (SCA) | To evaluate the gait characteristics of patients with SCA, as well as to analyze the correlation between gait parameters, clinical scales, and imaging on deep learning | 17 (9) | 47.6 ± 13.9 | 16 (11) | 48.1 ± 14.2 |  |  | Inertial sensor (JiBuEn gait analysis system) | The JiBuEn system comprises wearable devices of shoes and modules with inertial microelectromechanical system (MEMS) sensors attached under the shoe heel bottom and behind the upper and lower limbs and wrist. | Stride length, stride time, velocity, supporting-phase percentage, and swinging-phase percentage | Gait data consisting of 10 m of free walking of each individual in the SCA group and the gait control group were detected by wearable gait-detection equipment. Cerebellar volume and the midsagittal cerebellar proportion in the posterior fossa (MRVD) were calculated according to MR. Patients performed ataxia scales (ICARS and SARA). | There were significant differences in stride length, velocity, supporting-phase percentage, and swinging- phase percentage between the SCA group and the gait control group. The stride length and stride velocity of SCA groups were lower while supporting phase was longer than those of the gait control group. SCA group's velocity was negatively correlated with both the ICARS and SARA scores. The cerebellar volume and MRVD of the SCA imaging subgroup were significantly smaller than those of the imaging control group. MRVD was significantly correlated with ICARS and SARA scores, as well as stride velocity variability |
| Shah, et al. Mov. Disord., 2021 | Spinocerebellar ataxia (SCA) | To identify a set of gait measures from body-worn inertial sensors that best discriminate between people with prodromal or manifest spinocerebellar ataxia and age-matched healthy controls, and to determine how these measures relate to disease severity | 163 + 42 premanifest SCA |  | 96 |  |  |  | Inertial sensors (Opals by APDM Wearable Technology, an ERT company, Portland, OR, USA) | 6 on the dorsum of each foot and hand, on the sternum and on the lower lumbar vertebral segments | Mean of gait speed, stride time, double support time, variability of gait speed, stride time, and double support time, and also lateral step variability, mean of toe-out angle, toe-off angle, foot strike angle, foot elevation at mid swing, trunk sagittal range of motion [ROM], transverse ROM, coronal ROM, turn velocity, arm ROM, and the variability for each of these measures | Participants performed a natural pace, 2-minute walk wearing the sensors. | Increased gait variability was the most discriminative gait feature of SCA; toe-out angle variability (AUC = 0.936; sensitivity = 0.871; specificity = 0.896) and double-support time variability (AUC = 0.932; sensitivity = 0.834; specificity = 0.865) were the most sensitive and specific measures. These variability measures were also significantly correlated with the scale for the assessment and rating of ataxia (SARA) and disease duration. The same gait measures discriminated gait of people with prodromal SCA from the gait of HC (AUC = 0.610, and 0.670, respectively). |
| Zhou, et al. Neurol. Sci., 2022 | Spinocerebellar ataxia (SCA) | To objectively assess gait and balance in patients with SCA | 14 (9) | 61.6 ± 8.6 | 4 (4) | 49.0 ± 16.4 |  |  | Inertial sensors (LEGSysTM, BioSensics, Newton, MA) | 7 on the shin, thighs, wrists, and waist | Normalized stride length (normalized by height), stride duration, cadence, stance and swing phase, double support (initial, terminal, and total), stride time and stride length variability, medial–lateral pelvis sway distance, and turn duration | Participants performed two independent trials of gait and balance assessments from the Scale for the Assessment and Rating of Ataxia (SARA) and Brief Ataxia Rating Scale (BARS2) wearing the sensors. | Stride length variability, stride duration, cadence, stance phase, pelvis sway, and turn duration were different between SCA and controls (p < 0.05). Similarly, sway and sway velocity of the ankle, hip, and center of mass differentiated SCA and controls (p < 0.05). Using these features, linear regression models showed moderate-to-strong correlation with clinical scores from the in-person rater during SARA assessments of gait (r = 0.73, p = 0.003) and stance (r = 0.90, p < 0.001) and the BARS2 gait assessment (r = 0.74, p = 0.003). |
| Mohammadi-Ghazi, et al. Sensors (Basel), 2022 | Spinocerebellar ataxia (SCA) | To objectively assessing the upper-extremity motor symptoms in spinocerebellar ataxia (SCA) using data collected via a wearable sensor | 14 |  | 3 |  |  |  | Inertial sensors (LEGSysTM, BioSensics, Newton, MA, USA) | 2 on both wrists | Cycle time (duration of a full cycle), decline/rise time (duration of phases 1 and 3, respectively), pause duration at patient’s nose (duration of phase 2), pause duration at rater’s finger (duration of phase 4), number of cycles per second. Standard deviation (STD) and coefficient of variation (CoV) of all timing features, STD of angular velocity (AV), and linear acceleration (LA) Maximum, minimum, range, mean value, and root-mean-square of motion intensity of AV and LA and all three components of the AV, \|AV\|, LA, \|LA\|, speed metric of AV and MIAV. Jerk index of the motion intensity of AV and LA and all three components of the AV and LA. The first resonant frequency (RF) and the magnitude of resonant frequency (MR) of each component of AV and LA. The second RF and MR of each component of AV and LA. | Participants underwent a standardized ataxia evaluation (SARA and BARS version 2) wearing the inertial device. | The results showed excellent intra-rater reliability as the ICC was in the range of 0.94–0.99. The cycle detection technique showed an accuracy of 97.6% in a Bland–Altman analysis and a 94% accuracy in predicting the severity of the finger-to-nose test. The dependency of the upper-extremity tests was investigated through statistical analysis, and the results confirm dependency and potential redundancies in the upper- extremity SARA assessments. |
| Velázquez-Pérez, et al. Mov. Disord.,2021 | Spinocerebellar ataxia (SCA) | To describe novel preclinical biomarkers of subtle gait and postural sway abnormalities in prodromal spinocerebellar ataxia type 2 (pre-SCA2) | 30 (23) premanifest SCA2 | 43.5 ± 10.5 | 30 (23) | 43.3 ± 10.2 |  |  | Inertial sensors (The Opal TM, Mobility Lab, ver- sion 4.6.3.v20170301-0400, APDM Inc., Portland, OR, USA) | 6 on each hand, feet, on the sternum, and on the lower back (L5) | 1/ Lower-limb metrics (gait speed, stride length, foot elevation at mid-swing, toe- out angle, toe-off angle, double support period, and swing period) 2/ Lumbar/trunk metrics (ranges of motion in the coronal, sagittal, and transverse planes at the lumbar and trunk regions)  3/ Arm range of motion (ROM) | The experimental protocols for gait assessment included two tasks: natural gait and forward tandem gait.  The postural sway assessment comprised 3 tasks: natural stance, feet-together stance, and tandem stance. The scale for assessment and rating of ataxia (SARA) was performed to evaluate cerebellar signs, whereas non-cerebellar features were assessed using the Inventory of Non-Ataxia Symptoms (INAS) Scale. | Quantitative analysis of natural gait showed a significantly larger variability of the swing period, toe-off angle and toe-out angle in pre-SCA2, and larger mean coronal and transverse ranges of motion of the trunk at the lumbar location and of the sagittal range of motion of the trunk at the sternum location compared to controls. During tandem gait, pre-SCA2 subjects showed larger lumbar, trunk, and arm ranges of motion than controls. Postural sway analysis showed excessive body oscillation that was increased in tandem stance. Overall, these abnormalities were detected in pre-SCA2 patients without clinical evidence of abnormalities in SARA. The toe- off angle and swing time variability were significantly correlated with the time to ataxia onset, whereas the toe-off angle and transverse range of motion at trunk position during tandem gait were significantly associated with the SARA score. |
| Subramony, et al. J. Neurol. Sci., 2012 | Spinocerebellar ataxia (SCA) | To investigate the utility of home-based gait monitor assessment in patients with SCA | 19 | 56 ± 10.7 |  |  |  |  | Accelerometer (Step activity monitor (SAM), Cyma Inc. Seattle, USA) | 1 on the ankle | Percent time being inactive (TIA), percent time spent in low, moderate and high speeds of activity (TLA, TMA, THA), percent steps taken in low, moderate and high speeds (SLA, SMA, SHA), average daily step count (ADS-CT), average speed of steps (steps/minute, S/MT), and capacities for burst (maximum steps in any one minute epoch: MAX-1) and endurance (highest average steps in any 60 minute epoch: HGH-60) types of activities. | Patients performed the scale for assessment and rating of ataxia (SARA), disease staging, a timed 25 foot walk test and a 9 hole peg-board test. The patient wore the SAM bracelet for seven 24 hour periods at home. | The objective monitor measurements were highly associated with disease duration and with the functional stage of disease (p<0.01). Monitor measurements were significantly correlated to SARA scores with the exception of the percent of steps expended in moderate and high speeds of activity. Fewer monitor measures had significant correlations with walk and peg board scores. The objective SAM outputs also possessed high internal consistency, high intraclass correlation coefficients and could be fitted to a single factor by factor analysis. |
| Martindale, et al. Annu. Int. Conf. IEEE Eng. Med. Biol. Soc., 2018. | Hereditary Spastic Paraplegia (HSP) | To validate an unsupervised model based on local cyclicity estimation and hierarchical hidden Markov models to assess gait in Hereditary Spastic Paraplegia | 10 (6) | 58 ± 7 |  |  |  |  | Tri-axial accelerometer and gyroscope (Shimmer 2R, Shimmer Sensing, Dublin, Ireland) | 2 on the lateral side of the shoes | Stride time, swing time, stance time, swing duration and cadence. | Each subject performed two 4 x 10 m walks at a self-selected pace. The subjects were allowed to use their preferred walking aid. The GAITRite® pressure sensor carpet was used as a reference system. | This personalised hierarchical hidden Markov Model (hHMM) achieves a mean absolute error of 0.04 s ± 0.03 s for stride time estimation with respect to a GAITRite® reference system. |
| Martindale, et al. Annu. Int. Conf. IEEE Eng. Med. Biol. Soc., 2017 | Hereditary Spastic Paraplegia (HSP) | To determine which algorithms (the hidden Markov model (HMM) or dynamic time warping (DTW)) is the more effective to assess gait characteristics of patients with Hereditary Spastic Paraplegia | 21 (13) | 47.2 ± 13.3 |  |  |  |  | Tri-axial accelerometer and gyroscope (Shimmer 2R, Shimmer Sensing, Dublin, Ireland) | 2 on the lateral side of the shoes | Stride time, swing time, stance time, swing duration and cadence. | Each subject performed a 4 by 10 m walk at a self-selected speed while being recorded with a camera. | Using a nested cross validation for parameter choice and validation, the HMM was found to be superior for segmentation purposes with a mean segmentation error of 0.10 ± 0.05 s. |
| Regensburger, et al. Neurology, 2022 | Hereditary Spastic Paraplegia (HSP) | To investigate gait cyclicity parameters by application of a mobile gait analysis system in a cross-sectional cohort of patients with HSP and a longitudinal fast progressing subcohort | 112 (52) | 47.7 ± 15.2 | 112 (63) | 48.5 ± 13.8 |  |  | Inertial sensor (Shimmer 2R, Shimmer Sensing, Dublin, Ireland) | 2 on the shoes | Stride time, swing time, stance time, swing duration and coefficient of variation of each variable | Participants performed a 4 × 10 m walking test during regular visits in 3 outpatient centers. Patients were also rated according to the Spastic Paraplegia Rating Scale and in a subset, questionnaires on quality of life and fear of falling were obtained. An unsupervised segmentation algorithm was used to extract stride parameters and respective coefficients of variation. | Although swing time was unchanged compared with controls, there were significant increases in the duration of the total stride phase and the duration of the stance phase, both regarding absolute values and coefficients of variation values. Although stride parameters did not correlate with age, weight, or height of the patients, there were significant associations of absolute stride parameters with single SPRS items reflecting impaired mobility, with patients’ quality of life , and notably with disease duration. Sensor-derived coefficients of variation, on the other hand, were associated with patient-reported fear of falling and cognitive impairment. In a small 1-year follow-up analysis of patients with complicated HSP and fast progression, the absolute values of mobile gait parameters had significantly worsened compared with baseline. |
| O’Keefe, et al. Cerebellum, 2021 | Fragile X Syndrome (FXS) | To determine the impact of challenging gait conditions, including DT cognitive interference and fast-paced walking, on gait and turning deficits in individuals with FXTAS To investigate whether there are sex differences in DT and fast-paced gait performance in individuals with FXTAS, and the association between gait performance and falls under these challenging gait conditions | 30 (17) | 68.9 ± 8.8 | 35 (20) | 65.5 ± 8.3 |  |  | Inertial sensor (OpalTM, APDMTM, Oregon) | 4 on the dorsum of each foot, sternum and lumbar trunk (at L5) | Stride length, stride velocity, cadence, double support, swing, stride length CoV, stride velocity CoV, peak turn velocity, number of steps to turn | Participants underwent gait analysis using an inertial sensor-based 2-min walk test under three conditions: self-selected pace, fast pace, and dual-task with a concurrent verbal fluency task. | FXTAS participants had reduced stride length and velocity, swing time, and peak turn velocity and greater double limb support time and number of steps to turn compared to controls under all three conditions. There was greater dual task cost of the verbal fluency task on peak turn velocity in men with FXTAS compared to controls. Additionally, stride length variability was increased and cadence was reduced in FXTAS participants in the fast pace (FP) condition. Stride velocity variability under FP gait was significantly associated with the number of self-reported falls in the last year. Greater motor control requirements for turning likely made men with FXTAS more susceptible to the negative effects of DT cognitive interference. FP gait exacerbated gait deficits in the domains of rhythm and variability, and increased gait variability with FP was associated with increased falls. |
| O’Keefe, et al. Gait Posture, 2018 | Fragile X Syndrome (FXS) | To investigate the associations between executive function and information processing speed and gait, turning and falls in premutation carriers with and without FXTAS compared to healthy controls | 18 + 15 premutation carriers without FXTAS (11) | 62.7 ± 7.1 | 27 (9) | 69.1 ± 6.6 |  |  | Inertial sensor (APDM Mobility LabTM inertial sensor system, APDMTM; Oregon) | 6 on ankle, wrists, lower back and sternum. | Gait speed (stride length and velocity), Rhythm (cadence), gait variability (stride length and cadence variability) and gait cycle phase (percentage of gait cycle spent in swing and double limb support) | An inertial sensor based instrumented Timed Up and Go was employed to test gait, turns and functional mobility. The well validated and reliable instrumented 7m TUG (i-TUG) was performed three times by participants and the mean value for each gait parameter was calculated. Global cognition and the cognitive domains of information processing speed, attention, response inhibition, working memory and verbal fluency were tested with a neuropsychological test battery. | Lower information processing speed was significantly associated with shorter stride length, reflecting slower gait speed, in premutation carriers with FXTAS but not premutation carriers without FXTAS or controls. Lower response inhibition was also significantly associated with slower turn-to-sit times in premutation carriers with FXTAS but not in those without FXTAS or controls. |
| Mueller, et al. Ann. Clin. Transl. Neurol., 2021 | Friedreich's ataxia (FRDA) | To evaluate the utility of home-based, self-administered digital endpoints in children with Friedreich’s ataxia and unaffected controls and their relationship to standard clinical rating scales | 13 (10) | 13 ± 2 years | 12 (5) | 12 ± 4 |  |  | 1/ Hand motor function was analyzed using a digital pen (Anoto Pen, DPC Solutions, Morges, Switzerland) 2/ Speech tests were recorded on a computer or smartphone 3/ Triaxial accelerometer and gyroscope (GaitUp Physilog 5, Lausanne, Switzerland) | 3/ 5 on each foot, each wrist, and on the trunk | 1/Hand motor function: 14 parameters were calculated and roughly grouped into the following categories: veloc- ity, deviation of the actual from the “ideal” spiral, and frequency domain-based parameters 2/Automated analysis of speech : Automated Syllable Count Diff, automated Syllable Count, automated syllable rate, acceleration, instability, RValue, mean Syllable Duration, SD Syllable Duration, Mean Gap Length, SD Gap Length 3/15 gait parameters : steps per minute, double support, foot fully flat, gait cycle time, heel strike angle, loading,  3D foot path distance, peak swing, foot take-off, stride width, step speed, stance, swing width, foot turning angle, toe-off angle | In the screening clinic visit, families received equipment and training with the home-based digital devices and recorded their first session. At home, participants recorded digital endpoints for three assessments at specified days (but no specific time of the day) within 1 week and responded to a usability questionnaire for the tests and devices before returning the equipment. Participants were asked to wear the sensors during waking hours to record walking, balance, and arm movements during six days. | Hand-drawing and speech tests were easy to conduct and generated high-quality data. Hand-drawing parameters also strongly correlated with standard 9-hole peg test scores. The sensor-based gait and balance tests suffered from technical limitations in this study setup. Greater activity levels were measured for the foot sensors, followed by wrist and trunk sensors. The control group was significantly more active than the FRDA group with foot and wrist movements. Peak swing and stance period were the most discriminatory parameters between group. The FRDA group spent more time in stance phase and had high peak acceleration during swing time There was correlation between the 25-foot walk test and the cadence as estimated from real-world walking.   Digitally derived stride width strongly reflected the risk of falling. Mean stride width from the real-world gait analysis dropped with GAA repeat length of the short allele. |
| Corben, et al. Cerebellum, 2021 | Friedriech's ataxia (FRDA) | To evaluate the AIM-S as a sensitive and functionally relevant clinical outcome for use in clinical trials | 40 (20) : 1/ 31 adults 2/ 9 children | 1/ 38.2 ± 12  2/ 14 ± 3.1 | 20 : 1/ 19 adults  2/ I child | 1/ 33.8 ± 8.8 2/ 15 |  |  | The AIM-S system consists of three components: 1/ the data logger which is a spoon containing sensors, Wi-Fi and processor (the AIM-S data logger); 2/ algorithms that distinguish between movements made by control and ataxic users and grades the severity of ataxia when detected; and 3/ ataxia scores presented in a manner that has utility for clinicians (the AIM-S “score”). |  | AIM-S score | A prospective longitudinal study evaluated the capacity of the AIM-S to detect change in upper limb function over 48 weeks. Friedreich ataxia clinical severity, performance on the Nine-Hole Peg Test and Box and Block Test and responses to a purpose-designed questionnaire regarding acceptability of AIM-S were recorded. | The sensitivity of the AIM-S to detect deterioration in upper limb function was greater than other measures. Patient-reported outcomes indicated the AIM-S reflected a daily activity and was more enjoyable to complete than other assessments. The AIM-S is a more accurate, less variable measure of upper limb function in Friedreich ataxia than existing measures. The AIM-S is perceived by individuals with Friedreich ataxia to be related to everyday life and will permit individuals who are non-ambulant to be included in future clinical trials. |
| Pilzak, et al. Adv. Exp. Med. Biol., 2018 | Sarcoidosis | To define how sarcoidosis patients’ quality of life, daily physical activity, and physical performance are related to each other | 17 (7) | 46.8 ± 8.8 |  |  |  |  | Accelerometer (Actigraph GT3X+ device, Pensacola, FL) |  | Number of steps and the amount of energy expended during daily physical activity | Physical activity was assessed using accelerometry during 7 days (7am to 22pm). | Daily PA and VO2max were lower in sarcoidosis patients than the known predicted values in healthy age-matched individuals. |
| Cho, et al. Lung, 2019 | Sarcoidosis | To objectively assess physical activity in patients with pulmonary sarcoidosis and investigate its relationship with lung function, exercise capacity, symptom burden, and health status | 15 (11) | 52.7 ± 15.5 | 14 (10) | 46.5 ± 5.5 |  |  | Tri-axial accelerometer (ActivPal, PAL Technologies Ltd, Glasgow, UK) | 1 on the upper thigh. | Steps per day and sit-to-stand transition | Participants completed the questionnaires, spirometry and 6MWT prior to the application of the activity monitor. Participants were instructed to wear the activity monitor for 8 consecutive days. | Patients with sarcoidosis had significantly lower daily step counts than healthy controls and a trend towards fewer sit-to-stand transitions each day. Daily step count was significantly associated with 6MWT distance in sarcoidosis, but not with forced vital capacity, fatigue, dyspnoea or KSQ health status. Time spent upright was associated with fatigue and health status , and there was a significant correlation between the number of sit-to-stand transitions and MRC dyspnoea score. |
| Korenromp, et al. Chest, 2011 | Sarcoidosis | To assess the severity and characterize fatigue in patients with sarcoidosis in clinical remission and to characterize it according to the international criteria for chronic fatigue syndrome (CFS) | 75  1/ Fatiguated : 37 (25) 2/ Non fatiguated : 38 (17) | 1/ Fatiguated : 48.1 ± 7.4 2/ Non fatiguated : 46.2 ± 8.9 |  |  |  |  | Accelerometer (Actilog V3.0; Nijmegen, The Netherlands) | 1 on the ankle. | Acceleration per day | Assessements included questionnaires (Checklist Individual Strength [CIS], Symptom Checklist-90, Beck Depression Inventory for primary care, Medical Outcomes Study 36-Item Short-Form Health Survey), standardized interview (CFS criteria), sleep diary, accelerometer, and muscle strength tests. Participants instructed to wear this motion device during 14 consecutive days and nights, except while bathing, swimming, and during activities in rainy weather conditions. | Physical activity levels were reduced in fatigued participants. |
| Bahmer, et al. Respiration, 2018 | Sarcoidosis | To evaluate the relationships between daily physical activity and established clinical assessment parameters in patients with sarcoidosis and to explore whether fatigue and its different domains are associated with physical activity impairments in patients with sarcoidosis | 57 (25) | 49.7 ± 10.6 |  |  |  |  | Tri-axial accelerometer (SenseWear Armband, BodyMedia, Inc., Pitts burgh, PA, USA) | 1 on the upper left arm. | Steps per day | Physical activity was assessed in daily living for 1 week. Lung function (DLCO, FVC), exercise capacity (6-min walking distance), health-related quality of life (St George’s Respiratory Questionnaire), generic quality of life (12-Item Short-Form Health Survey), and fatigue were also assessed. | The MFI-20 subscales “reduced activity” and “physical fatigue” were weakly associated with steps per day on a bivariate level (Spearman ρ = –0.274 and ρ = –0.277, respectively; p < 0.05), while the other subscales and the total score were not. 6MWD, SGRQ score, and SF-12 (physical health) score showed stronger associations with steps per day in bivariate analyses (Pearson r = 0.499, r = –0.386, and r = 0.467, respectively; p < 0.01), and were independent predictors of steps per day in multivariate linear regression analyses adjusting for confounders (p < 0.05). In ROC curve analyses, 6MWD, SGRQ score, and SF-12 (physical health) score properly identified sedentary patients (steps per day <5,000; AUROC 0.90, 0.81, and 0.80, respectively; p < 0.01). Fatigue was less predictive (MFI-20 subscale “general fatigue,” AUROC 0.70; p = 0.03). |
| Chu, et al. JMIR Mhealth Uhealth, 2022 | Sarcoidosis | To investigate whether smartphones could assess the quality of life and physical activity of a large cohort of individuals with sarcoidosis. | 629 (404) | 51 ± 10.9 |  |  |  |  | Sarcoidosis App | 1 | Average daily step | Individuals with sarcoidosis were recruited, consented, and enrolled entirely within the app. Surveys on sarcoidosis history, medical history, and medications were administered. Patients completed modules from the Sarcoidosis Assessment Tool, a validated patient-reported outcomes assessment of physical activity, fatigue, pain, skin symptoms, sleep, and lungs symptoms. Physical activity measured by smartphones was tracked as available. | Both QoL related to physical activity (P<.001) and fatigue (P<.01) correlated with actual smartphone-tracked physical activity. |
| Berntsen, et al. Rheumatology (Oxford), 2019 | Dermatomyositis | To compare cardiorespiratory fitness expressed as maximal oxygen uptake (VO2max) between patients with long-term juvenile dermatomyositis and controls and between patients with active and inactive disease, as well as to explore exercise limiting factors and associations between fitness and disease variables. | 45 (28) | 28.9 ± 12 | 45(28) | 29.2 ± 12 |  |  | Accelerometer (GT3X, Actigraph, Pensacola, FL, USA) |  | Time spent in light physical or moderate to vigorous physical activity (MVPA) and the total time with count per minute | Participants performed a cardiopulmonary exercise test (CPET) on a treadmill until exhaustion. Disease activity, damage and muscle strength/function were assessed by validated tools. Participants were instructed to wear the device during all waking hours not spent in water for 8 consecutive days and to keep a habitual level of physical activity. | Patients with inactive disease had lower physical activity levels compared with controls. |
| Stephens, et al. J. Rheumatol., 2016 | Dermatomyositis | To examine the face, content, and construct validity of the stage of exercice scale (SOES) in determining the readiness for exercise in children with rheumatic disease. | 15 (9) | 15.5 ± 2 |  |  | 1/ 39 (30) patients with Juvenil idiopathic arthritis  2/ 13 (3) patients with cystic fibrosis | 1/ 14.3 ± 2  2/ 14 ± 2.1 | Accelerometer (Actical) | 1 on the right hip | Time spent in light physical or moderate to vigorous physical activity (MVPA) | Each participant wore an inertial over a 7-days period during waking hours except for when they were swimming or bathing. | Children and adolescents in higher stages (SOES) participated in more minutes of vigorous physical activity compared with those in the lower stages (SOES). |
| Pinto, et al. Semin. Arthritis Rheum., 2016 | Dermatomyositis | To objectively measure physical activity levels in a cohort of juvenile dermatomyositis patients and to associate physical activity variables with disease-related parameters, physical capacity, and health-related quality of life. | 19 | 14.6 ± 3.9 | 19 | 14.6 ± 3.8 |  |  | Accelerometer (Actigraph GT3Xs,ActiGraph, Pensacola, FL) | 1 on the right side of the hip | Sedentary time, light-intensity physical activity and moderate-to-vigorous physical activity | Participants were instructed to wear the accelerometer for 7 consecutive days during waking hours, except when bathing. | Only one of the 19 patients (5%) achieved the minimum recommended moderate-to-vigorous physical activity levels. Sedentary time was positively correlated with disease duration and negatively with VO2 peak. MVPA was negatively associated with disease duration, and positively associated with VO2 peak, and current use of corticoid. |
| Battaglia, et al. Clin. Respir. J., 2017 | Scleroderma | To evaluate this performance and its correlates, in patients with SSc compared with healthy controls, in a free-living setting | 27 (23) | 45.2 ± 12.2 | 11 (3) | 41.5 ± 5.3 |  |  | Bi-axial accelerometer (SenseWear Armband, Bodymedia, Pittsburgh, PA, USA) | 1 on the upper right arm | Physical activity duration (PAD; in minutes) for non-sedentary activities and physical activity level (PAL = total daily energy/resting energy expenditure) per day | Physical activity was assessed by a device worn for at least 6 days. Nutritional status was estimated by bioelectrical impedance analysis and pulmonary arterial hypertension excluded by echocardiography. | Daily physical activities and PAL were significantly reduced in patients with scleroderma compared with controls. Seventy-four per cent of patients with scleroderma showed PAL < 1.70, whereas only 27% of controls were below this threshold for sedentary life style. Both PAD and PAL positively correlated with diffusing capacity of the lungs (DLCO). In patients with scleroderma, exercise capacity during daily activity was reduced compared with controls, and was associated with early evidence of functional decay (decreasing DLCO but not with malnutrition. |
| Kraan, et al. J. Intellect. Disabil. Res., 2022 | Prader-Willi Syndrom (PWS) | To test the feasibility of ‘real-world’ instrumented gait assesment using the Physilog®5 wearable in children with PWS and Angelman Syndrom | 9 (5) |  |  |  | 5 (2) patients with Angelman Syndrome |  | Inertial sensors (Gait Up Physilog®5, Lausanne, Switzerland) | 1 on the ankle | Mean and variability results for stride time, cadence, stance percentage and stride lenght | The PWS group completed two indoor gait assessments: laboratory assessment within a research centre (15 m track; 3–4 laps with turns around a pole; ‘real-world’ long walk assessment of 2 min in a quiet public hospital corridor with one turn. | This approach was found to be feasible, with all participants able to complete at least one assessment. This study also demonstrated significant agreement, using Lin’s concordance correlation coefficient (CCC), between laboratory and ‘real-world’ assessments in the PWS group for mean stride length, mean stance % and stance % CV (n = 7, CCC: 0.782–0.847, P = 0.011–0.009). |
| Belluscio, et al. Hum. Mov. Sci., 2019 | Prader-Willi Syndrom (PWS) | To develop and evaluate the efficacy of pathology-specific and personalized rehabilitation treatments | 11 (4) | 5.3 | 12 (4) | 6.10 | 15 (9) children with Down syndrom | 6.63 | Tri-axial accelerometer and tri-axial gyroscope (Opal, APDM Inc., Portland, Oregon, USA) | 4 on the pelvis, sternum, and both distal tibiae | Spatiotemporal parameters (walking speed, stride frequency, and stride length) and a set of indices related to gait symmetry and upper-body stability (Root Mean Square, Attenuation Coefficient and Improved Harmonic Ratio) | Participants performed a 10-meter walking test while wearing inertial sensors. The Gross Motor Functional Measures (GMFM-88) and Intelligence Quotient (IQ Wechsler) were also assessed for each patient. | Children with Down syndrom and PWS exhibit reduced gait symmetry and higher accelerations at pelvis level than controls. While these accelerations are attenuated by about 40% at sternum level in controls and down syndrom, PWS children display significant smaller attenuations, thus reporting reduced gait stability, most likely due to their typical “Trendelenburg gait”. Significant correlations were found between the estimated parameters and the GMFM-88 scale when considering the whole PWS and down syndrom group and the PWS group alone. |
| Cimolin, et al. Comput. Methods Biomech. Biomed. Engin., 2020 | Prader-Willi Syndrom (PWS) | To quantify gait parameters during level walking in adults with PWS who were compared to unaffected individuals | 20 (11) | 35.6 ± 11.2 | 20 (11) | 35.7 ± 10.6 |  |  | Tri-axial accelerometer, tri-axial gyroscope and tri-axial magnetometer (G-SensorVR , BTS Bioengineering, Italy) | 1 on lower back (L4-L5) | Gait speed, stride length, cadence, stance phase, swing phase, double support phase, harmonic ratio (a measure of step-to-step symmetry based on trunk acceleration processing in the three directions) | Participants were requested to walk along a straight trajectory identified at the center of a 20-m hallway at a self- selected speed in the most natural manner. Four trials were acquired for each participant | While no differences between the two groups were found in terms of spatio-temporal parameters, individuals with PWS exhibited significantly reduced values of harmonico ratio (HR) in the antero-posterior and vertical directions. Such results, which indicate a poorer gait symmetry in PWS, suggest that upper body accelerations, as well as HR, provide novel information on gait in people with PWS that could not be extracted from spatio-temporal parameters only. |
| Castner, et al. Res. Dev. Disabil., 2014 | Prader-Willi Syndrom (PWS) | To characterize physical activity (PA) in youth with PWS and to compare it to PA in children with nonsyndromal obesity | 24 (12) | 11.2 ± 2.3 |  |  | 40 (19) obese children without PWS | 9.8 ± 1.1 | Tri-axial accelerometer (GT3X; Actigraph, LLC, Pensacola, FL, USA) | 1 on the right hip | Sedentary behavior (SED), light (LPA), moderate (MPA), vigorous (VPA) and moderate plus vigorous (MVPA) | Participanst wore accelerometers for eight consecutive days. | Youth with PWS spent 19.4% less time in weekly LPA and 29.8% less time in weekly VPA compared to obese controls. All other intensities were similar between groups. In addition, PWS participated in less LPA and VPA during the weekends compared to controls, and less LPA on weekdays when compared to obese. There was also a trend towards PWS participating in less MVPA during the weekends and less VPA during the weekends than obese controls. There was a trend towards PWS participating in less VPA on weekends compared to weekdays, while obese participated similarly in VPA on weekdays and weekend days. On average, neither PWS nor obese children met minimum MVPA recommendations. |
| Woods, et al. Food Nutr. Res., 2018 | Prader-Willi Syndrom (PWS) | To determine associations between body composition, diet, physical activity (PA), and a timed walk for adults with Prader-Willy syndrome, and to assess adequacy of dietary intake for those individuals aging with Prader-Willy syndrome | 19 (8) | 34.5 ± 4.3 |  |  |  |  | Accelerometer (ActivPal®) | 1 on the middle of the right thigh | Steps per day and position, standing versus sitting/lying | Data collection involved obtaining anthropometric measurements, body composition, a walk test, PA mea- surements, food intake information, and a brief medical history. This study was approved by the University of Oklahoma Health Sciences Center Institutional Review Board. The accelerometer was worn for up to 7 days, with a goal of a minimum of 4 days of usable data. | Patients had healthier body composition, at 26.8% body fat, than previously reported. Mean body mass index (BMI) was in the overweight range at 26.7. Those who consumed higher amounts of fat (as a percent of total kilocalories) had statistically significant lower body fat percentage, but this may simply reflect that individuals with lower body fat percentages felt freer to consume fat. Mean steps taken per day was 7631.7 steps but only 16% of participants met healthy PA recommendations despite participating in daily structured exercise. |
| Duran, et al. J. Pediatr. Endocrinol. Metab., 2016 | Prader-Willi Syndrom (PWS) | To determine if physical activity is associated with bone health in children with Prader-Willi syndrome | 23 (12) | 11 ± 2 |  |  |  |  | Tri-axial accelerometer (GT3X; Actigraph, LLC, Pensacola, FL, USA) | 1 on the right hip | Time spent in light (LPA), moderate (MPA), moderate-to-vigorous physical activity (MVPA)and vigourous (VPA) physical activity | Participants were instructed to wear the accelerometer for 8 consecutive days (Sunday to Sunday) during all waking hours, except for bathing/showering or swimming. Hip, total body minus the head (body), bone mineral content (BMC), bone mineral density (BMD) and BMD z-score (BMDz) were measured by dual X-ray absorptiometry. | Moderate PA and select covariates explained the most variance in hip BMC (84.0%), BMD (61.3%) and BMDz (34.9%; p < 0.05 for all). For each body parameter, moderate PA and select covariates explained the most variance in body BMC (75.8%), BMD (74.4%) and BMDz (31.8%; p < 0.05 for all). |
| Bellicha, et al. J. Appl. Res. Intellect. Disabil., 2020 | Prader-Willi Syndrom (PWS) | To objectively quantify spontaneous physical activity (PA) in adult patients with Prader–Willi and to evaluate the transferability of a home‐based exercise training programme in these patients | 10 (10) | 30 |  |  | 20 (10) adult with non‐syndromic obesity | 28.8 | Tri‐axial accelerometer (GT3x Actigraph, Manufacturing Technology, Inc.) | 1 on the hip | Time spent in light (LPA), moderate (MPA), moderate-to-vigorous physical activity (MVPA)and Vigourous (VPA) physical activity | Participants were asked to wear the accelerometers for seven consecutive days during all waking hours except during water‐based activities. In the PWS group, PA, body composition, walking capacity, quality of life and eating behaviour were then compared before and after a 16‐week supervised exercise programme. | The PWS group displayed lower PA and higher sedentary time compared to the control group. Median attendance to exercise sessions reached 100% sessions. Moderate‐to‐vigorous PA and walking capacity increased after the programme without significant effect on body composition. |
| Rubin, et al. Med. Sci. Sports Exerc., 2019 | Prader-Willi Syndrom (PWS) | To test the effectiveness of a home-based physical activity (PA) intervention led by parents in youth with obesity with and without Prader Willy syndrome to increase moderate-to-vigorous PA (MVPA) and gross motor proficiency | 45 |  |  |  | 66 patient with obesity without Prader Willy syndrome |  | Tri-axial accelerometer (GT3X;Actigraph, Pensacola, FL) | 1 on the hip | Total PA (TPA), time spent in light (LPA), moderate (MPA), moderate-to-vigorous physical activity (MVPA) and Vigourous (VPA) physical activity | Intervention participants completed a PA curriculum 4 days per week for 24 weeks. Pre–post outcomes (baseline to 24 week) included MVPA and motor proficiency including upper limb coordination, bilateral coordination, balance, running speed and agility, and muscle strength (Bruininks–Oseretsky Test of Motor Proficiency). Patients wore an accelerometer at home during 7 days. | The intervention led to no change in MVPA (I group, 39.6 vs 38.9 min/d; C group, 40.6 vs 38.3 min/d). The intervention led to improvements in body coordination (22.3%; p < 0.05), as well as strength and agility (13.7%; p < 0.05). Specifically, the I group showed increases in upper limb coordination (19.1%), bilateral coordination (27.8%), and muscle strength (12.9%; p <0.05 for all) not observed in the C group: -0.2%, 2.5%, and -3.2%, respectively. |
| Hamed, et al.  NPJ Digit. Med., 2019 | Pompe disease | To evaluate the willingness to adopt wearable devices for passive and active health monitoring and to explore the relationship between patient characteristics and disease experience (symptoms and impact) with device-measured mobility | 29 (26) | 43 ± 10 |  |  |  |  | Tri-axial accelerometer (Fitbit OneTM) | 1 on the belt, pocket, or bra | Median step count and peak 1-min activity | Data were captured over 6 to 8 weeks through a wearable activity tracker (daily) and self-assessment (weekly and daily) via a website, with an exit survey at the conclusion of the study. | In the analyses cohort, engagement in data sharing was high (94% of patients uploaded data for more than half the study days). Mean step count differed by age (20–39 years: 4071 vs. 40–69 years: 2394, p < 0.01), diagnostic delay (<10 years: 3584 vs. ≥10 years: 2232, p < 0.05), disease duration (<10 years: 4219 vs. ≥10 years: 2462, p < 0.05), and ambulatory status (aided: 1883 vs. unaided: 3408, p < 0.05). Patient-reported “fatigue and pain” score was inversely correlated with step count (Pearson’s r = −0.42, p < 0.05) and peak 1-min activity (Pearson’s r = −0.49, p < 0.01). |
| Hobson-Webb, et al. Mol. Genet. Metab. Rep., 2021 | Pompe disease | To evaluate quantitative muscle ultrasonography (QMUS) and electrical impedance myography (EIM) as potential biomarkers | 25 (16) | 50.1 ± 26 |  |  |  |  | Electrical impedance myography (ImpediMed, Inc., Carlsbad, CA, USA) and handheld electrical impedance myography (AIM, Skulpt, Inc) |  | Body fat % measurement for each muscle and a Muscle Quality (MQ) score derived from raw EIM data | Patients were evaluated at baseline, 12 months and 24 months. Muscle ultrasonography, standard electrical impedance myography and handheld electrical impedance myography (hEIM) were compared with the clinical data. Five patients were given hEIM devices to perform measurements at home. | QMUS and hEIM had good reliability as measures of muscle structure and conduction properties. Home, patient-performed hEIM measurements did not differ significantly from those performed in the clinic setting. Thirteen patients completed all follow-up measures. Most measures did not change over the study period, however, vastus lateralis echointensity increased 27%, a sign of declining muscle health. Additionally, significant correlations between QMUS, hEIM and measures of muscle strength and function were present. |
| Mumford, et al. J. Neurodev. Disord., 2015 | Mucopolysaccharidosis (MPS) | To study disturbance in circadian rhythm functioning in MPS type III population | 8 (3) | 9.2 ± 4.9 | 8 (4) | 8.6 ± 4.8 |  |  | Cambridge Neurotechnology AW4 or Respironics Actiwatch 2 actigraph | 1 on the non-dominant wrist | 1/ L5 and M10 onset specify the average time of the start of the least active 5-h period (L5) and the most active 10-h period (M10) 2/ Relative amplitude is derived from the normalised difference between the most active 10-h period and least active 5-h period in an average 24-h  3/ Intra-daily variability indicates fragmentation of an individual’s rhythm 4/ Interdaily stability specifies the invariability of 24-h rhythm between days  5/ Periodicity specifies the time of the peak correlation of the ‘best fit’ of any given rhythm within the parameters of the expected circadian cycle of 24 h±15 min. 6/ levels of activity were measured across each of the four quadrants of a 24-h period  7/ Total activity counts were also generated for each day and averaged across the recording period. | Actigraphic data were recorded in children with MPS III over 7–10 days. Parameters of circadian rhythmicity and activity levels across a 24-h period were analysed. | Statistically and clinically significant differences between the two groups were noted. Analysis indicated that children with MPS III showed significantly increased fragmentation of circadian rhythm and reduced stability with external cues (zeitgebers), compared to controls. Average times of activity onset and offset were indicative of a phase delayed sleep-wake cycle for some children in the MPS III group. Children with MPS III had significantly higher activity levels during the early morning hours (midnight–6 am) compared to controls. |
| Vallim, et al. Chronobiol. Int., 2019 | Fabry | To evaluate the rhythmic profile in Fabry’s disease patients using rhythmicity markers | 1/ 11(5) classic  2/ 6 (5) nonclassic variant | 1/ 38.9 ± 3.6  2/ 46.2 ± 4.9 | 14 (10) | 42.4 ± 3.2 |  |  | Condor Instruments ActTrust device |  | Activity evaluation : duration of the rhythms (period) and the stability and rhythmic prominence (percentage of variation), mean value (mesor), the time of maximum value (acrophase), the difference between the mesor and maximum or minimum value (amplitude), the level of daytime (M10) and nighttime (L5) activities. | They recorded activity and body temperature rhythms by an actigraphy during at least 10 days and collected urine to assess 6-sulfatoxymelatonin excretion load during the day (from the second urine in the morning until 7 p.m.) and night (starting from 7 p.m. until the first urine in the morning of the following day). | No significant differences were found on the rhythmic parameters of activity and rest. |
| Davies, et al. Orphanet J. Rare Dis.,2020. | GM2 gangliosidosis (GM2) | To assess the feasibility of using digital health technology to monitor GM2 patients remotely between hospital visits | 8 (5) | 44 ± 11 |  |  |  |  | A wearable device and a smartphone application |  | Average daily maximum (ADM), average daily steps (ADS), and average daily steps per 30-min epoch (ADE) | Laboratory and clinical data including GaitRIte data and 6 minutes walk distance were collected. Pariticipants wre ask to wear the device during 6 months. | Adherence rate for wearing the device and completing the mPROs was 84 and 91%, respectively, resulting in a rich multidimensional dataset. As expected for a six-month proof-of-concept study in a disease that progresses slowly, statistically significant changes were not expected or observed in the clinical, mPROs, or wearable device data. |
| McErlane, et al. Ther. Innov. Regul. Sci., 2021 | Niemann-Pick C (NP-C) and Duchenne Muscular dystrophy (DMD) | To determine the utility of wearable technologies in physical activity assessment in three paediatric diseases, namely, Niemann-Pick C (NP-C), Juvenile Idiopathic Arthritis (JIA) and Duchenne Muscular Dystrophy (DMD). | 10 (4) | 10 | 0 |  | 1/ 8 patients with DMD (0) 2/ 12 patients with JIA (6) | 1/ 11  2/ 13.5 | Tri-axial accelerometer | 1 on the wirst | Average daily maximum (ADM): the mean of daily epochs with the most steps, calculated over a month; average daily steps (ADS): the mean of the total number of daily steps, calculated over a month; and average steps per epoch (ASE): the mean number of steps in each 30-min epoch, calculated over a day. Active days (days with > 4 h of data) were then averaged over a month. | Participants completed the 6-min walk test (6MWT) at enrolment. Patients were provided with disease-specific smartphone apps paired with a wearable device. In the DMD and JIA studies, children were asked to wear the device continuously for 12 weeks, but the NP-C study patients were asked to wear the device with no predefined set duration. | Median 6MWT results were 450 m, 325 m and 434.5 m for the NP-C, DMD and JIA cohorts, respectively. Wearable data capture was feasible in all three disease cohorts, although complete data capture was not sustained. A statistically significant between-cohort difference was identified for ADM, ADS and ASE. Statistically significant differences were found between DMD/JIA for ADM; NP-C/DMD for ADS and DMD/JIA for ASE. |
| El-Masri, et al. Neurol. Sci., 2022 | Niemann-Pick C (NP-C) | To explore the relationship between objective measurements of movement obtained via the use of the Personal KinetiGraph (PKG) with the clinical information obtained via questionnaires and clinical rating tools of patients with Niemann-Pick type C. | 12 (6) | 38 ± 13.2 | 0 |  | 0 |  | Accelerometer (Personal KinetiGraph) | 1 on the most affected wrist | Bradykinesia score (BK), dyskinesia (DK) score (these score were further divided into their interquartile ranges), fluctuation score (FDS), percentage time immobile (PTI), and percent time with tremors (PTT) | Participants wore the PKG for 6 days during regular activities. Clinical assessments included Abnormal Involuntary Movement Scale (AIMS), Epworth Sleepiness Score (ESS), Falls, Neuropsychiatric Unit Assessment Tool (NUCOG), Parkinson’s disease questionnaire (PDQ), and modified Unified Parkinson’s Disease Rating Scale (UPDRS) which were performed over telehealth within 2 weeks of PKG use. | We found bradykinesia to be a feature among this cohort of patients, with a median BKS of 22.0 (7.4). Additionally, PTI scores were elevated at 4.9 (8.2) indicating elevated daytime sleepiness. Significant correlations were demonstrated between BK25, BK50 and BK75. FDS correlated with PDQ, UPDRS IV, UPDRS and AIMS. DK25 in comparison with NUCOG-A and DK75 in comparison with NUCOG and NUCOG-A demonstrated significant correlations. Additionally, duration of illness in comparison with PTI demonstrated significance. |
| Suresha, et al. medRxiv, 2022 | Rett syndrome | To develop machine learning classification models to classify patients with low-severity Rett syndrome rom patients with high-severity Rett syndrome based on the objective measures attained from a wearable biosensor. | 20 |  | 0 |  | 0 |  | Tri-axial accelerometer (BioStamp®,MC10 Inc., Cambridge, MA, USA) | 4 on thorax (2) and abdomen (2) | Activity count, interday stability, intraday variability, least active 5 hours, most active 10 hours, rest activity, mesor, amplitude, and acrophase. | Caretaker and physician surveys were conducted to obtain symptom severity for all 20 patients enrolled in the study (CGI-S scores). For each patient-visi, two consecutive days of signal data was needed for the feature extraction. | Physio-motor features were derived from the recordings that captured heart rate variability, activity metrics, and the interactions between heart rate and activity. A machine learning (ML) models was used to classify high-severity Rett patients from low-severity Rett patients using the derived physio-motor features. For the best-trained model, the results showed a pooled area under the receiver operating curve equal to 0.92 via a leave-one-out-patient cross-validation approach. |
| Downs, et al. Disabil. Rehabil., 2015 | Rett syndrome | To investigate the capacity of three accelerometer-type devices to measure walking activity in Rett syndrome | 26 | 18 ± 8 | 0 |  | 0 |  | 1/ Tri‐axial accelerometer (GT3x Actigraph, Manufacturing Technology, Inc.) 2/ A uni-axial accelerometer and an inclinometer (ActivPAL,PAL Technologies Ltd, Glasgow, UK) 3/ Accelerometer (Stepwatch Activity Monitor (SAM), Modus Health LLC, Washington, DC, USA) | 1/ 1 on the waist 2/ 1 on the thigh 3/ 1 on the ankle | 1/ Measures activity counts, vector magnitude and steps 2/ Steps count and time spent in supine and sitting from the time in standing and walking. 3/ Step count | Participant was fitted with all three devices and encouraged to undertake physical activities for 20 to 30 min with supervision and assistance as necessary. Activities, which included walking at different speeds and on different terrains and inclines, were video-taped by an investigator. For the video-taped observations, a step was counted when the foot cleared the ground and moved in either a forward, sideways or backward direction. Two trained observers counted steps during each video session and mean values were used for analyses. | The mean difference (limit of agreement) for the Actigraph, ActivPAL and SAM were -41 (SD 33),-16 (SD 21) and -1 (SD 16) steps/min, respectively. Agreement was influenced by a device/cadence interaction with greater under-recording at higher cadences. For SAM data, repeatability of step-count pairs was excellent (intraclass correlation coefficient 0.91, 95% CI 0.79–0.96). The standard error of measurement was 6 steps/min and we would be 95% confident that a change more than 17 steps/min would be greater than within-subject measurement error. |
| Stahlhut, et al. J. Child Neurol., 2017 | Rett syndrome | To validate measures of sedentary time in individuals with Rett syndrome | 26 | median age 16 | 0 |  | 0 |  | Uni-axial accelerometer and inclinometer (ActivPAL,PAL Technologies, Glasgow, UK) | 1 on the thigh | Time spent in sitting, standing and walking | Paritcipants wore the device during video-taped activities. Participants were recorded during a 20- to 30-minute session during which they were encouraged to undertake physical activities that mostly included standing and walking. Then, 11 participants fit their child with an activPAL over a 7-day period and to complete a modified Bouchard activity record over 24-hours within this period, on a day that they were with their child all day. | In comparison to observation with video, the activPAL accurately measured duration of sedentary time with a mean difference (limit of agreement) of 1.0 (6.3) minutes. Linear regression was used to determine the relationship between sedentary time recorded on the modified Bouchard activity record diary card and measured using the activPAL. The duration of Bouchard activity record downtime accounted for 73% of the variance of sedentary time measured by the activPAL. |
| Downs, et al.  Dev. Med. Child Neurol., 2017 | Rett syndrome | To quantify, in individuals with Rett syndrome with the capacity to walk, walking-based activity and sedentary time, and to analyse the influences of age, walking ability, scoliosis, and the severity of epilepsy | 64 | 17.6 ± 9 | 0 |  | 0 |  | Accelerometer (Stepwatch Activity Monitor (SAM), Modus Health LLC, Washington, DC, USA) | 1 on the right ankle | Average daily steps and sedentary time | Participant wore a StepWatch Activity Monitor for at least 4 days. Linear regression models were used to assess relationships between daily step count and the proportion of waking hours spent in sedentary time with the covariates of age group, walking ability, presence of scoliosis, and frequency of seizures. | On average, 62% (SD 19%) of waking hours were sedentary and 20% (SD 8%) was at cadences lower than or equal to 20 steps in a minute. The median daily steps count was 5093 (interquartile range 2026–8602). Compared with females younger than 13 years of age and accounting for the effects of covariates, adults took fewer steps, and both adolescents and adults had more sedentary time. |
| Stahlhut, et al.  Phys. Ther., 2020 | Rett syndrome | To evaluate the feasibility and health-related effects of an individualized 12-week uptime participation (U-PART) intervention in girls and women with Rett | 14 (14) | median age 18.7 | 0 |  | 0 |  | 1/ A uni-axial accelerometer and an inclinometer.(ActivPAL,PAL Technologies Ltd, Glasgow, UK) 2/Accelerometer (Stepwatch Activity Monitor (SAM), Modus Health LLC, Washington, DC, USA) | 1/ 1 on the thigh  2/ 1 on the ankle | 1/ time spent in a sitting/lying or standing position 2/ Daily step count | During a 12-week intervention period, individualized programs focused on participation in enjoyable uptime activities in home, school/day center, and community settings. Feasibility was assessed with a study-specific questionnaire. Primary outcome measures were sedentary time and daily step count. Secondary outcomes were gross motor skills, walking capacity, quality of life, and goal attainment scaling. Participants who could walk short distances in training sessions or participants who were not ambulatory but able to stand wore the activPAL for 7 days at each time point (T0–T3). Participants who were functional ambulators wore both the activPAL and the SAM for 7 days at each time point (T0–T3). | The U-PART intervention was perceived as feasible by caregivers. Similar scores were observed at baseline assessments in all outcomes. Positive effects with small to medium effect sizes were seen in sedentary time (− 4%), daily step count (+ 689 steps/d), walking capacity (+ 18.8 m), quality of life (+ 2.75 points), and goal attainment scaling after the intervention. Positive effects were maintained in sedentary time (− 3.2%) and walking capacity (+ 12.1 m) at short-term follow-up. |
| Earnest, et al. Brain Sci., 2020 | Tuberous Sclerosis Complex (TSC) | To objectively measure movement levels using actigraphy in individuals with a complex neurodevelopmental genetic disorder, tuberous sclerosis (TSC) | 30 (20) |  |  |  |  |  | Move 3 actigraphs (movisens GmbH; Karlsruhe, Germany) | 2 on the right hip and on the wrist of their non-dominant hand | Mean movement (average movement acceleration over 10-s intervals, averaged for the entire duration of the tasks or movies), threshold movement (number of accelerations greater than 0.01 G per minute, sampled at 64 Hz, averaged for the entire duration of the tasks or movies) and the coefficient of variation (CoV) (mean/standard deviation * 100) for these measurements | Participants underwent brief (approximately 1 h) daytime actigraph assessment during two settings: movie viewing and cognitive testing. Correlations were used to examine associations between actigraph measures and parent-rated ADHD symptoms and other characteristics of TSC (symptoms of autism spectrum disorder (ASD), intellectual ability (IQ), epilepsy severity, cortical tuber count). | Higher movement levels during movies were associated with higher parent-rated ADHD symptoms. Higher ADHD symptoms and actigraph-measured movement levels during movies were positively associated with ASD symptoms and negatively associated with IQ. Inter-individual variability of movement during movies was not associated with parent-rated hyperactivity or IQ but was negatively associated with ASD symptoms. There were no associations with tuber count or epilepsy. |
| Bornstein, et al. J. Sleep Res., 2021 | Narcolepsy | To compare PA levels and measures of metabolic activity in narcolepsy type 1 with the primary hypersomnolence disorders, narcolepsy type 2 and idiopathic hypersomnia | 1/ 56 (29) patients with narcolepsy type 1  2/ 23 (19) patients narcolepsy type 2 or idiopathic hypersomnia | 1/ 27.6 ± 9.1 2/ 32.6 ± 10.4 |  |  |  |  | Sensewear® armband accelerometer model MF-SW, Bodymedia |  | Metabolic equivalent of task (MET), the average time of physical activity, step count, total energy expenditure, temperature and sleep duration and efficiency | Accelerometry-derived measures of physical activity, total energy expenditure and skin temperature were collected from patients during seven consecutive days without medication. In addition, results from multiple sleep latency tests and the Epworth Sleepiness Scale questionnaire, body weight, height and CSF orexin/hypocretin were acquired. | However, none of these measurements were significantly different between groups Thus, by analysing accelerometric data, we could not find any differences in the amount of physical activity or total energy expenditure explaining overweight in narcolepsy type 1. |
