# Supplementary material for: The use of digital outcome measures in clinical trials in rare neurological diseases: a systematic literature review
Source: Orphanet J Rare Dis. 2023 Aug 2;18:224. doi: 10.1186/s13023-023-02813-3 (PMC10398976; doi:10.1186/s13023-023-02813-3)
Supplement: Supplementary file 3 — Additional file 3. Results of quality assessment. [file 13023_2023_2813_MOESM3_ESM.docx]

**Additional file 3**

| Quantitative non-randomized controlled studies | | | | | |
| --- | --- | --- | --- | --- | --- |
| Reference | Are the participants representative of the target population? | Are measurements appropriate regarding both the outcome and intervention (or exposure)? | Are there complete outcome data? | Are the confounders accounted for in the design and analysis? | During the study period, is the intervention administered (or exposure occurred) as intended? |
| Le Moing, et al.  PLoS One, 2016 | Can't tell | Y | Y | N | Y |
| Fujii, et al. Brain Dev., 2019 | Can't tell | Y | Y | N | Can't tell |
| Ganea, et al.  J. Child Neurol., 2012 | Can't tell | Y | Y | Y | Y |
| Davidson, et al. J. Child Neurol., 2015 | Can't tell | Y | Y | Y | Can't tell |
| Fowler, et al. Muscle Nerve, 2018 | Y | Y | N | N | Can't tell |
| Lott, et al. Muscle Nerve, 2021 | Y | Y | N | Can't tell | Can't tell |
| An, et al. Chaos, 2020 | Y | Y | Y | Y | Y |
| Jeannet, et al.  Eur. J. Paediatr. Neurol., 2011 | Can't tell | Y | Y | N | Can't tell |
| Capelini, et al.  Neuropsychiatr. Dis. Treat., 2017 | Y | Y | Y | Y | Y |
| Kimura, et al.  Pediatr. Int., 2014 | Can't tell | Y | Can't tell | N | Can't tell |
| van der Geest, et al.  Muscle Nerve, 2020 | Can't tell | Y | Can't tell | N | Can't tell |
| Killian, et al. Neuromuscular Disord., 2020 | Can't tell | Y | N | N | Can't tell |
| Siegel, et al.  Muscle Nerve, 2020 | Can't tell | Y | Can't tell | N | Can't tell |
| Arteaga, et al.  J. Neuromuscular Dis., 2020 | Y | Y | N | Can't tell | Can't tell |
| Ferrer-Mallol, et al. Front. Pharmacol., 2022 | Can't tell | N | Can't tell | N | Can't tell |
| Jacques, et al.  Disabil. Rehabil., 2022 | Can't tell | N | Y | N | Y |
| van Eijk, et al. J. Neurol., 2019 | Y | Y | Y | N | Can't tell |
| Garcia-Gencedo, et al. JMIR Mhealth Uhealth, 2019 | Can't tell | N | N | N | Can't tell |
| Kelly, et al. Amyotroph. Lateral Scler. Frontotemporal Degener., 2020 | Can't tell | Y | N | N | Can't tell |
| Liao, et al.  Front. Aging Neuroscience, 2022 | Y | Y | Y | N | Can't tell |
| Geronimo, et al. J. Med. Eng. Technol., 2021 | Y | Y | Y | N | Y |
| Rutkove, et al. Ann. Clin. Transl. Neurol., 2020 | Y | N | N | N | Can't tell |
| Pancani, et al.  PLoS One, 2017 | Can't tell | Y | Y | Y | Y |
| Pancani, et al. Clin. Biomech. (Bristol, Avon), 2018 | Can't tell | Can't tell | Y | N | Y |
| Vieira, et al.  NPJ. Digit. Med., 2022 | Y | Y | Can't tell | N | Can't tell |
| Agurto, et al.  Annu. Int. Conf. IEEE Eng. Med. Biol. Soc., 2019 | Y | Y | Can't tell | N | Can't tell |
| Baxi, et al. Nat. Neurosci., 2022 | Y | Can't tell | Can't tell | Y | Can't tell |
| Londral, et al. Clin. Neurophysiol., 2016 | Can't tell | Y | Y | Can't tell | Y |
| Vogelnik, et al. J. Neurol., 2022 | Can't tell | Can't tell | N | N | Y |
| Crook-Rumsey, et al. Muscle Nerve, 2022 | Can't tell | Y | Y | N | Y |
| Meyer, et al. Amyotroph. Lateral Scler. Frontotemporal Degener., 2022 | Y | Y | N | N | Can't tell |
| Peterson, et al.  Gait Posture, 2021 | Y | Y | Y | N | Y |
| Menotti, et al. Brain Behav., 2014 | Can't tell | Y | Can't tell | Y | Can't tell |
| Ramdharry, et al. Disabil. Rehabil., 2017 | Can't tell | Y | Can't tell | Can't tell | Can't tell |
| Pazzaglia, et al. Neuromuscular Disord., 2019 | Y | Y | Can't tell | N | Can't tell |
| Padua, et al. Eur. J. Neurol., 2016 | Y | Y | Can't tell | N | Can't tell |
| Saifee, et al. Clin. Neurophysiol., 2015 | Can't tell | N | Y | N | Y |
| Knak, et al. J. Neurol., 2020 | Y | Y | Y | Y | Can't tell |
| Jimenez-Moreno, et al. Disabil. Rehabil., 2019 | Y | Y | Y | Can't tell | Y |
| Bachasson, et al. Neuromuscular Disord., 2016 | Can't tell | Y | Y | Y | Y |
| Hamel, et al. Muscle Nerve, 2022 | Y | Y | Y | Y | Can't tell |
| Gidaro, et al. Muscle Nerve, 2022 | N | Y | Can't tell | N | Can't tell |
| Huisinga, et al Muscle Nerve, 2018 | Can't tell | Y | Y | N | y |
| Statland, et al. Muscle Nerve, 2019 | Can't tell | Y | Y | N | Can't tell |
| Maleki, et al. JMIR Form. Res., 2022 | Y | Y | Can't tell | Can't tell | Can't tell |
| O’Connor, et al. J. Neuromuscular Dis., 2019 | Can't tell | Y | Can't tell | N | Can't tell |
| Gordon, et al. Digit. Biomark., 2019 | Can't tell | Y | Y | N | Can't tell |
| Adams, et al. Digit. Biomark., 2017 | Can't tell | Y | Y | Y | Can't tell |
| Gil Polo, et al. Ann. Nutr. Metab., 2015 | Can't tell | Y | Can't tell | Y | Can't tell |
| Dinesh, et al. J. Huntingtons Dis., 2020 | Can't tell | Y | N | Y | Y |
| Andrzejewski, et al. J. Huntingtons Dis., 2016 | Can't tell | Y | Y | Can't tell | Can't tell |
| McLaren, et al.  Neuropsychology, 2021 | Y | Y | Can't tell | Y | Can't tell |
| Waddell, et al.  J. Huntingtons Dis., 2021 | Can't tell | Y | Y | Y | Can't tell |
| Lang, et al. J. Huntingtons Dis., 2021 | Can't tell | N | N | Can't tell | Can't tell |
| Cohen, et al. BMC Med. Inform. Decis. Mak., 2018 | Can't tell | N | Y | N | Can't tell |
| Lipsmeier et al., J. Med. Internet Res., 2022 | Y | Y | Y | Can't tell | Can't tell |
| Gaßner, et al. J. Neurol., 2020 | Y | Y | Y | Y | Y |
| Trojaniello, et al.  Gait posture, 2015 | Can't tell | N | Y | Can't tell | Y |
| Purcell, et al. PLoS One, 2020 | Can't tell | Y | Y | Y | Y |
| Dalton, et al. Gait Posture, 2013 | Can't tell | Y | Y | Can't tell | Y |
| Mannini, et al.  Annu. Int. Conf. IEEE Eng. Med. Biol. Soc. 2015 | Can't tell | N | Y | Can't tell | Y |
| Keren, et al. Front. Neurol., 2021 | Y | Y | Y | Can't tell | Can't tell |
| Porciuncula, et al. Neurorehabil. Neural Repair 2020 | Can't tell | Y | Y | Can't tell | Y |
| Kegelmeyer, et al. J. Neurol. Sci., 2017 | Y | Y | Y | Can't tell | Y |
| Desai et al. Clin. Biomech. (Bristol, Avon), 2022 | Y | Y | Y | Can't tell | Y |
| Muratori, et al. Hum. Mov. Sci., 2021 | Y | Y | Can't tell | Y | Y |
| Desai, et al. Gait Posture, 2021 | Can't tell | Y | Y | Can't tell | Y |
| Bennasar, et al. IEEE Trans. Neural Syst. Rehabil. Eng., 2018 | Can't tell | Y | Y | Can't tell | Y |
| Mannini, et al. Sensors (Basel), 2016 | Can't tell | Y | Y | Can't tell | Y |
| Lauraitis, et al. J. Healthc. Eng., 2018 | Can't tell | N | Y | Can't tell | Y |
| Shih, et al. medRxiv, 2022 | Can't tell | Can't tell | Y | N | Can't tell |
| Raccagni, et al. Brain Behav.,2018 | Can't tell | Y | Y | Can't tell | Y |
| Hatanaka, et al. Eur. Neurol., 2016 | Can't tell | Y | Y | Can't tell | Y |
| Baston, et al. Gait Posture, 2014 | Can't tell | Y | Y | Y | Y |
| Pilotto, et al. Cerebellum, 2021 | Can't tell | Can't tell | Y | N | Y |
| Djuric-Jovicic, et al. J. Clin. Neurosci., 2016 | Can't tell | Y | Y | Can't tell | Y |
| De Vos, et al. Gait Posture, 2020 | Can't tell | Y | Y | Y | Y |
| Sotirakis, et al. Movement Disorders, 2022 | Can't tell | Y | Y | N | Y |
| Panyakaew, et al. J. Neurosci., 2020 | Can't tell | Y | Y | Y | Y |
| Delrobaei, et al. J. Neuroeng. Rehabil., 2016 | Can't tell | N | Y | N | Y |
| Rudzińska, et al. Neurol. Neurochir. Pol., 2013 | Y | Y | Y | Can't tell | Y |
| Nieuwhof, et al. Neuroimage Clin., 2022 | Can't tell | Can't tell | Y | Y | Y |
| Elble, et al. Mov. Disord. Clin. Pract., 2017 | Can't tell | Y | Y | N | Y |
| Celletti, et al. Clin. Biomech. (Bristol, Avon), 2021 | Can't tell | Y | Y | Y | Y |
| Park, et al. J. Medical and Biological Eng., 2019 | Can't tell | Y | Y | N | Y |
| Zhang, et al. Ann. Clin. Transl. Neurol., 2022 | Can't tell | Y | Y | N | Y |
| Mason, et al. Trem. Other Hyperkinet. Mov. (NY), 2022 | N | N | Y | N | Y |
| Hickey, et al. Physiol. Meas., 2016 | Can't tell | N | Y | Can't tell | Y |
| Shah, et al. IEEE Trans. Biomed. Eng., 2020 | Y | Y | Y | Can't tell | Y |
| Kanzler, et al. Ann. Clin. Transl. Neurol., 2022 | Y | Y | N | Y | Can't tell |
| Jin, et al. Artif. Intell. Med., 2020 | Can't tell | Y | Y | Y | Y |
| Shah, et al. Mov. Disord., 2021 | Y | Y | Y | Can't tell | Y |
| Zhou, et al. Neurol. Sci., 2022 | Can't tell | Y | Y | Can't tell | Y |
| Mohammadi-Ghazi, et al. Sensors (Basel), 2022 | Can't tell | Y | Y | Can't tell | Y |
| Velázquez-Pérez, et al. Mov. Disord.,2021 | Y | Y | Y | Y | Y |
| Subramony, et al. J. Neurol. Sci., 2012 | Can't tell | Y | Can't tell | N | Can't tell |
| Martindale, et al. Annu. Int. Conf. IEEE Eng. Med. Biol. Soc., 2018 | Can't tell | N | Y | N | Y |
| Martindale, et al. Annu. Int. Conf. IEEE Eng. Med. Biol. Soc., 2017 | Can't tell | N | Y | N | Y |
| Regensburger, et al. Neurology, 2022 | Y | Y | Y | Y | Y |
| O’Keefe, et al. Cerebellum, 2021 | Y | Y | Y | Y | Y |
| O’Keefe, et al. Gait Posture, 2018 | Can't tell | Y | Y | N | Y |
| Mueller, et al. Ann. Clin. Transl. Neurol., 2021 | Can't tell | Y | Y | Y | Can't tell |
| Corben, et al. Cerebellum, 2021 | Y | Y | N | Can't tell | Y |
| Pilzak, et al. Adv. Exp. Med. Biol., 2018 | Can't tell | Y | Can't tell | N | Can't tell |
| Cho, et al. Lung, 2019 | Can't tell | Y | Y | Can't tell | Can't tell |
| Korenromp, et al. Chest, 2011 | Y | N | N | N | Can't tell |
| Bahmer, et al. Respiration, 2018 | Y | Y | Y | N | Can't tell |
| Chu, et al. JMIR Mhealth Uhealth, 2022 | Y | Y | N | N | Can't tell |
| Berntsen, et al. Rheumatology (Oxford), 2019 | Y | Y | Can't tell | Y | Y |
| Stephens, et al. J. Rheumatol., 2016 | Can't tell | N | Y | N | Can't tell |
| Pinto, et al. Semin. Arthritis Rheum., 2016 | Can't tell | Y | Y | Y | Can't tell |
| Battaglia, et al. Clin. Respir. J., 2017 | Can't tell | Y | Y | Can't tell | Can't tell |
| Kraan, et al. J. Intellect. Disabil. Res., 2022 | Can't tell | Can't tell | N | N | Y |
| Belluscio, et al. Hum. Mov. Sci., 2019 | Can't tell | Y | Y | Y | Y |
| Cimolin, et al. Comput. Methods Biomech. Biomed. Engin., 2020 | Can't tell | Y | Y | Y | Y |
| Castner, et al. Res. Dev. Disabil., 2014 | Can't tell | Y | Can't tell | Y | Can't tell |
| Woods, et al. Food Nutr. Res., 2018 | Can't tell | N | Can't tell | N | Can't tell |
| Duran, et al. J. Pediatr. Endocrinol. Metab., 2016 | Can't tell | N | Can't tell | N | Can't tell |
| Bellicha, et al. J. Appl. Res. Intellect. Disabil., 2020 | Can't tell | Y | Can't tell | Y | Can't tell |
| Rubin, et al. Med. Sci. Sports Exerc., 2019 | Y | Y | N | Can't tell | Can't tell |
| Hamed, et al.  NPJ Digit. Med., 2019 | Can't tell | Y | Y | N | Can't tell |
| Hobson-Webb, et al. Mol. Genet. Metab. Rep., 2021 | Can't tell | Y | Y | N | Can't tell |
| Mumford, et al. J. Neurodev. Disord., 2015 | Can't tell | Y | Can't tell | Y | Can't tell |
| Vallim, et al. Chronobiol. Int., 2019 | Can't tell | Y | Can't tell | Y | Can't tell |
| Davies, et al. Orphanet J. Rare Dis.,2020. | Can't tell | N | Y | N | Can't tell |
| McErlane, et al. Ther. Innov. Regul. Sci., 2021 | Can't tell | Y | Can't tell | N | Can't tell |
| El-Masri, et al. Neurol. Sci., 2022 | Can't tell | Y | Y | N | Can't tell |
| Suresha, et al. medRxiv, 2022 | Can't tell | Y | Y | N | Can't tell |
| Downs, et al. Disabil. Rehabil., 2015 | Can't tell | Y | Y | N | Y |
| Stahlhut, et al. J. Child Neurol., 2017 | Can't tell | N | Y | N | Can't tell |
| Downs, et al.  Dev. Med. Child Neurol., 2017 | Y | N | N | N | Can't tell |
| Stahlhut, et al.  Phys. Ther., 2020 | Can't tell | Y | Can't tell | N | Can't tell |
| Earnest, et al. Brain Sci., 2020 | Y | Y | Y | N | Y |
| Bornstein, et al. J. Sleep Res., 2021 | Y | N | Y | N | Can't tell |
| Quantitative randomized controlled trials | | | | | |
| Reference | Is randomization appropriately performed? | Are group comparable at baseline? | Are there complete outcome data? | Are outcome assessors blind to the intervention provided? | Did the participants adhere to the assigned intervention? |
| Davidson, et al.  Clin. Nutr., 2021 | Y | Y | N | Y | N |
| Shrader, et al. Ann. Clin. Transl. Neurol., 2015 | Y | Y | Y | Y | Y |
| Quantitative randomized controlled trials | | | | | |
| Reference | Is sampling strategy relevant to address the research question? | Is the sample representative of the target population? | Are measurement appropriate? | Is the risk of nonresponse bias low? | Is the statistical analysis appropriate to answer the research question? |
| Pancani, et al.  PLoS One, 2017 | Y | Y | Can't tell | Can't tell | Y |
